# Protocol for a cluster randomized clinical trial of a mastery-climate motor skills intervention, Children’s Health Activity and Motor Program (CHAMP), on self-regulation in preschoolers

**DOI:** 10.1371/journal.pone.0282199

**Published:** 2023-03-09

**Authors:** Leah E. Robinson, Kara K. Palmer, Lu Wang, Katherine Q. Scott-Andrews, Katherine M. Chinn, Indica Sur, Carissa Wengrovius, Emily Meng, Sanne L. C. Veldman, Alison L. Miller

**Affiliations:** 1 School of Kinesiology, University of Michigan, Ann Arbor, Michigan, United States of America; 2 Department of Biostatistics, School of Public Health, University of Michigan, Ann Arbor, Michigan, United States of America; 3 Department of Health Behavior and Health Education, School of Public Health, University of Michigan, Ann Arbor, Michigan, United States of America; University of Study of Bari Aldo Moro, ITALY

## Abstract

**Introduction:**

Self-regulation (SR) is critical to healthy development in children, and intervention approaches (i.e., professional training, classroom-based curricula, parent-focused intervention) have shown to support or enhance SR. However, to our knowledge, none have tested whether changes in children’s SR across an intervention relate to changes in children’s health behavior and outcomes. This study, the Promoting Activity and Trajectories of Health (PATH) for Children-SR Study uses a cluster-randomized control trial to examine the immediate effects of a mastery-climate motor skills intervention on SR. Secondly, this study examines the associations between changes in SR and changes in children’s health behaviors (i.e., motor competence, physical activity, and perceived competence) and outcomes (i.e., body mass index and waist circumference) (ClinicalTrials.gov Identifier, NCT03189862).

**Methods and analysis:**

The PATH—SR study will be a cluster-randomized clinical trial. A total of 120 children between the ages of 3.5 to 5 years of age will be randomized to a mastery-climate motor skills intervention (n = 70) or control (n = 50) condition. SR will be assessed using measures that evaluate cognitive SR (cognitive flexibility and working memory), behavioral SR (behavioral inhibition), and emotional SR (emotional regulation). Health behaviors will be assessed with motor skills, physical activity, and perceived competence (motor and physical) and health outcomes will be waist circumference and body mass index. SR, health behaviors, and health outcomes will be assessed before and after the intervention (pre-test and post-test). Given the randomization design, 70 children in the intervention group and 50 in the control group, we have 80% power to detect an effect size of 0.52, at a Type I error level of 0.05. With the data collected, we will test the intervention effect on SR with a two-sample t-test comparing the intervention group and the control group. We will further evaluate the associations between changes in SR and changes in children’s health behaviors and health outcomes, using mixed effect regression models, with a random effect to account for within-subject correlations. The PATH-SR study addresses gaps in pediatric exercise science and child development research. Findings hold the potential to help shape public health and educational policies and interventions that support healthy development during the early years.

**Ethics and dissemination:**

Ethical approval for this study was obtained through the Health Sciences and Behavioral Sciences Institutional Review Board, University of Michigan (HUM00133319). The PATH-SR study is funded by the National Institutes of Health Common Fund. Findings will be disseminated via print, online media, dissemination events and practitioner and/or research journals.

**Trial registration number:**

ClinicalTrials.gov Identifier, NCT03189862.

## Introduction

Self-regulation (SR) is a central area of research inquiry in child development. SR refers to the voluntary control of cognitive, emotional, and behavioral impulses in accordance with a long-term goal [1, 2], and it is required to sustain concentration and behavioral control while engaging in challenging tasks. SR is comprised of multiple interrelated processes that emerge rapidly across early childhood. These processes include cognitive skills that facilitate working memory, cognitive flexibility, and attention shifting (i.e., elements of executive function), behavioral skills that allow children to inhibit impulsive behaviors in favor of measured responses (i.e., behavioral inhibition), and emotional regulation skills that enable children to calm down from elevated or intense emotions [3]. The benefits of SR for long-term social, behavioral, and academic outcomes (e.g., academic success, school readiness, and classroom behavior) are well established [4, 5]. From a Science of Behavior Change (SOBC) perspective [6], SR is a behavioral mechanism that may underlie many different behaviors relevant for health and well-being; thus, enhancing SR early in life may have a translational, long-term impact on health [7, 8].

Due to the important role that SR has in the growth and development of children, there have been efforts to enhance SR in young children. Curriculum- or classroom-based approaches that use a combination of teachers’ professional training and classroom-based activities based on a defined curriculum, such as Head Start REDI [9] and Diamond’s Tools of the Mind [10] curricula, have shown to support social-emotional skills and problem-solving tactics. Parent-focused interventions that focus on routines and parent-child interactions [11] along with direct training to promote specific aspects of SR such as behavioral inhibition [12, 13] are also successful approaches to enhance SR. Despite some promising findings that SR can be promoted during childhood and adolescence [14] and SR-focused interventions can impact social emotional (REDI) and early academic skills [9], we know less about the effects of SR on other developmental domains.

SR has been studied as a key mechanism of health behavior change in relation to a wide range of health outcomes in adults [6, 15] and has been proposed as a behavioral mechanism that may promote positive health outcomes such as weight status during childhood [8, 16–20]. SR is hypothesized to support health in multiple ways; including shaping an individual’s capacity to focus on long-term goals and aiding in stress reduction [21–26]. For example, SR can help individuals maintain a healthy weight by “tuning-out” external cues in the moment (e.g., advertisements about food), reducing unhealthy emotional coping behaviors (e.g., sedentary behavior or frequent/stress eating), and sustaining engagement in positive health behaviors (e.g., physical activity and exercise). Although most studies linking SR to health outcomes and behaviors have been in adults [27–30], some observational work in adolescents [31, 32] has linked poorer SR to increased sedentary behavior and decreased physical activity [30, 32]. Other cross-sectional [16, 20] and longitudinal [16, 18–20, 33] studies found that better SR among toddler-and preschool-aged children was associated with healthier weight status, and that early-life SR may have long-term health benefits for children [8], even into adulthood [26]. These established associations of SR with health behaviors and health outcomes, in conjunction with research suggesting that SR can be enhanced through intervention, demonstrates a need for more studies that: 1) test novel interventions to improve SR, and 2) test SR as a mechanism of behavior change in children and youth.

Motor skills, goal-directed actions of the muscles that are categorized as gross and fine motor skills, are essential to children’s growth and development [34–37] From a SR perspective, learning motor skills may help individuals evoke, process, and regulate emotions [38–40]. Few studies have explored SR from a motor perspective in children. van der Fels, Te Wierike, Hartman, Elferink-Gemser, Smith and Visscher [38] conducted a systematic review that examined the relationship between cognitive and motor skills in typically developing children ages 4–16 years. van der Fels et al. concluded that there is insufficient evidence ‘for or against’ many correlations between motor and cognitive skills. However, the review found that fine motor skills, bilateral body coordination, and timed motor tasks demonstrated the strongest relationship to cognitive skills while balance, strength, and agility were less related. The authors speculate that the relationship might be because the former skills are more complex motor tasks and have a higher cognitive demand or load while the others require less cognitive demand. van der Fels et al’s review also demonstrated that stronger relationships between motor and cognitive skills are seen in pre-pubertal children compared to their counterparts. From an experimental standpoint, Becker, Miao, Duncan and McClelland [39] found that prekindergarten and kindergarten children’s visuomotor skills measured with the Beery visual-motor integration assessment were related to inhibitory control, working memory, and behavioral SR. There appears to be a link between motor and SR in children [38, 39] but the evidence is quite insufficient. Work from Becker, van der Fels, and Westendorp suggest that movement/motor skills interventions that used complex motor skills, like sequenced movement patterns or movement coordinated to rhythm, support higher order cognitive skills and tasks that could improve SR in young children.

The Children’s Heath Activity Motor Program (CHAMP) is a mastery-climate motor skills intervention grounded in achievement goal theory [41–45]. CHAMP adheres to Epstein’s TARGET structure (*T*ask, *A*uthority, *R*ecognition, *G*rouping, *E*valuation, and *T*ime) while teaching motor skills to young children and requires children to self-select, self-manage, self-evaluate, and self-direct themselves throughout the intervention setting [46]. Table 1 defines the foundational components of CHAMP and links to SR. These self-determined actions have the potential to support multiple aspects of SR by encouraging children to manage their emotions, focus attention, persist, plan and evaluate their actions while promoting motor skills and perceived motor competence [8, 40, 47, 48]. Robinson, Palmer and Bub [40] conducted a CHAMP efficacy trial and found that preschoolers in the CHAMP condition maintained delayed gratification (i.e., measured with the snack delay task) over time while those in the control group experienced a significant decrease in their scores. The efficacy trial also found that CHAMP led to significant improvement in preschoolers’ locomotor and ball skills [40]. This finding supports a connection between mastery climate motor skills interventions and SR and provides a rationale for using motor-based interventions to positively change SR in addition to health behaviors such as physical activity [36, 49–52] and motor skills [48, 53–55].

**Table 1:** Foundations of CHAMP intervention and links to SR.

TARGET Structure	Use of TARGET Structure in CHAMP to Promote SR	Example of Application
**Task:** Provide a variety of tasks/activities that vary in difficulty	Self-select from tasks/activities that vary in difficulty *(create goals and strategies*, *plan and implement actions*, *make decisions*, *self-manage*, *self-monitor*, *and self-correct behavior)*	3–4 motor skill stations included in the intervention each day Each station will have at least three levels of difficulty. For example- catch station will include at least three different catchable items- scarfs (easy), yarn balls (medium), and tennis balls (difficult)
**Authority:** Foster by allowing children to actively participate in the decision making process	Self-manage and self-monitor behaviors *(create goals and strategies*, *plan and implement actions*, *make decisions*, *self-manage*, *self-monitor*, *and self-correct behavior*, *manage emotions*, *understand and appropriately navigate social environments)*	The authority of CHAMP is shared between instructors and children. Instructors shared authority duties include: maintaining a safe learning environment, teaching motor skills, providing individualized feedback to children during the lesson, and encouraging children to engage in the daily stations Child shared authority duties include: self-selecting how, when and where to engage in the motor skill practice, deciding who they want to play with, and creating and managing their own social and emotional environments within CHAMP.
**Recognition:** Instructor and child recognize individual progress. Feedback is provided privately and individually.	Self-monitor and evaluate own performance *(self-monitor behaviors*, *self-reflection of progress*, *manage emotions*, *focus attention*, *persist on a task*, *understand and appropriately navigate social environments*, *collaborative efforts)*	Each child’s individual improvements are privately recognized by the instructor.
**Grouping:** Focuses on grouping patterns Children are not grouped but given opportunity to self-select their engagement with others	Self-select own engagement in task; give child ability to self-govern learning experience *(plan actions and make decisions*, *self-monitor behavior*, *self-correct behaviors*, *manage emotions*, *appropriately navigate social environments*, *collaborative efforts)*	Children decide who they will navigate the intervention with. They can decide if they want to play in groups or individually. No children will be forced into groups of any kind.
**Evaluation:** Determine progress based on self-norms not global norms	Self-evaluate own performance *(self-monitor behaviors*, *self-reflection of progress*, *manage emotions*, *focus attention)*	Gains accomplished in CHAMP are not benchmarked against external performance expectations. CHAMP instructors are not teaching children so that they can gain a higher percentile in a test, they are helping children evaluate their performance based on self-referenced standards.
**Time:** Individualize pace of instruction and learning experience	Self-direct own learning *(plan actions and make decisions*, *self-monitor behaviors*, *self-correct behaviors*, *manage emotions)*	Children can self-pace through the multiple learning stations. Children can decide if they want to engagement on only one station or all the stations as well as can decide how long to stay any station.

It is also well-established in the motor development literature that motor skills and perceived competence (i.e., how one perceived his or her own abilities in varying domains) are critical to multiple aspects of health (i.e., physical activity, cardiorespiratory fitness, muscular strength, muscular endurance, and a healthy weight status) [34]. For example, perceived motor competence (i.e., how one perceives his or her own motor performance) is a strong correlate of physical activity in children and youth [35, 56–61]. Barnett, Morgan, van Beurden and Beard [56] demonstrated the strong mediating role of perceived motor competence between motor skills and physical activity over the childhood years. While there is ample preliminary evidence that CHAMP directly promotes all these outcomes, motor skills [40, 47, 48, 62–67], physical activity [49, 67, 68], and perceived motor competence [47, 48, 63]; less is known regarding the effect of CHAMP on SR outcomes [40]. So, while there is preliminary evidence that CHAMP supports some SR skills in young children [40], additional research is needed. Further, SR is a multi-dimensional construct and additional research is needed to evaluate how CHAMP may impact various aspects of SR including cognitive skills, behavioral skills other than delayed gratification, and emotional regulation in young children.

The body and brain work harmoniously together, and more studies are needed to investigate the role of movement-based interventions on SR and the secondary effect of SR on health outcomes. We propose a cluster randomized controlled trial (RCT) designed to enhance early SR, health behaviors (i.e., motor competence, perceived motor competence, and physical activity), and health outcomes (i.e., waist circumference and body mass index) in preschool-aged children. Specifically, we will examine the immediate (pre- to post-test) effects of CHAMP on SR, and associations between changes in SR and changes in health behaviors and outcomes. The specific aims and hypothesis of this study will be to:

Aim 1. Examine the immediate (pre- to post-test) intervention effects of CHAMP (compared to control participants) on cognitive SR (cognitive flexibility, working memory, attention shifting), behavioral SR (behavioral inhibition) and emotional SR (emotion regulation).

Hypothesis 1. Preschoolers in CHAMP will demonstrate greater improvements from pre-to-posttest in SR (cognitive SR, behavioral SR, and emotional SR) compared with the control condition.

Aim 2. Examine the associations between SR (cognitive SR, behavioral SR, and emotional SR) and changes in health behaviors (motor competence, perceived competence, physical activity) and health outcomes (body mass index, waist circumference).

Hypothesis 2. Preschoolers’ SR (cognitive SR, behavioral SR, and emotional SR) will be positively associated with changes in health behaviors (motor competence, perceived competence, and physical activity) and health outcomes (body mass index, waist circumference) from pre-to-posttest.

## Methods/Design

### Study design

**Fig 1:**
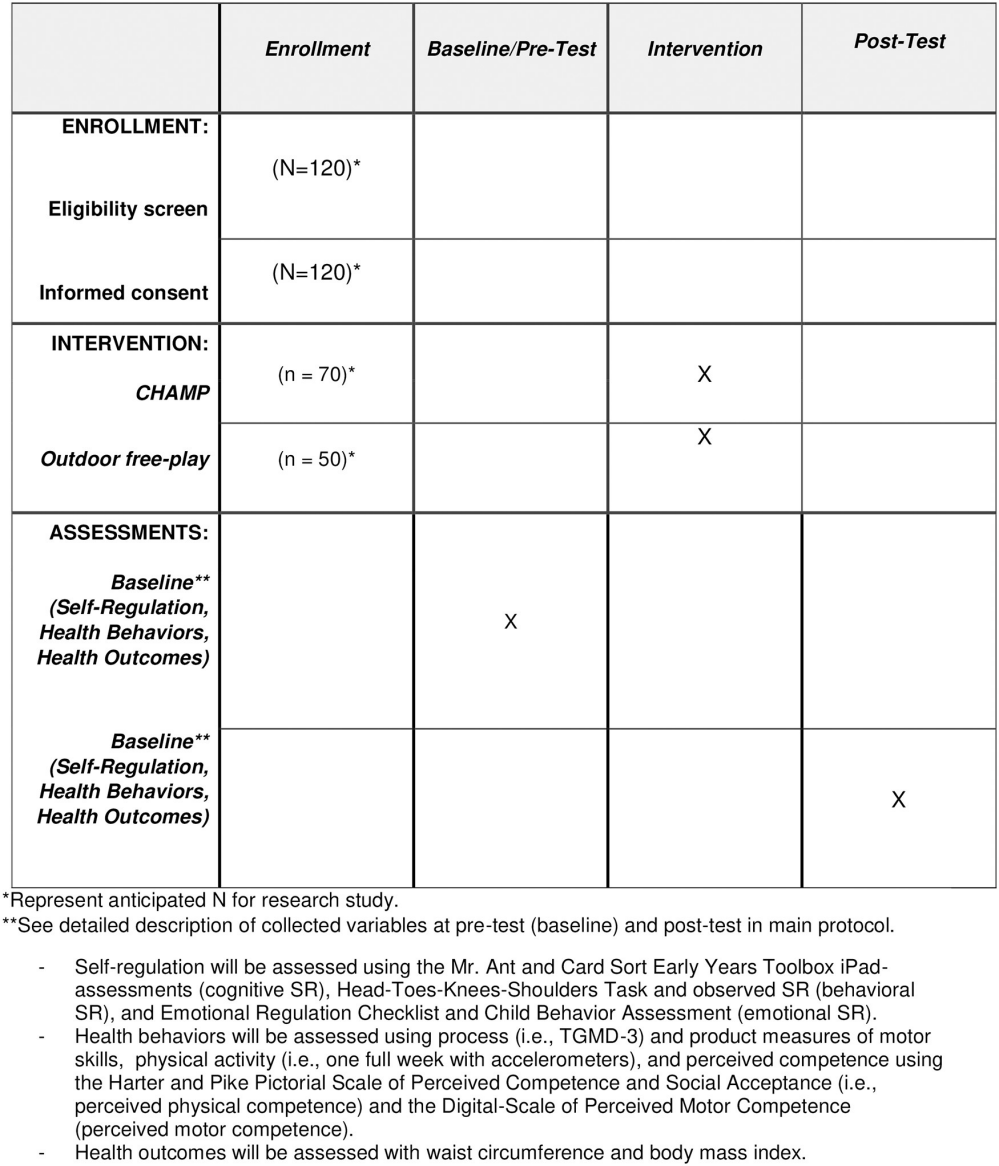
SPIRIT diagram for the schedule of enrollment, interventions, and assessments.

**Fig 2:**
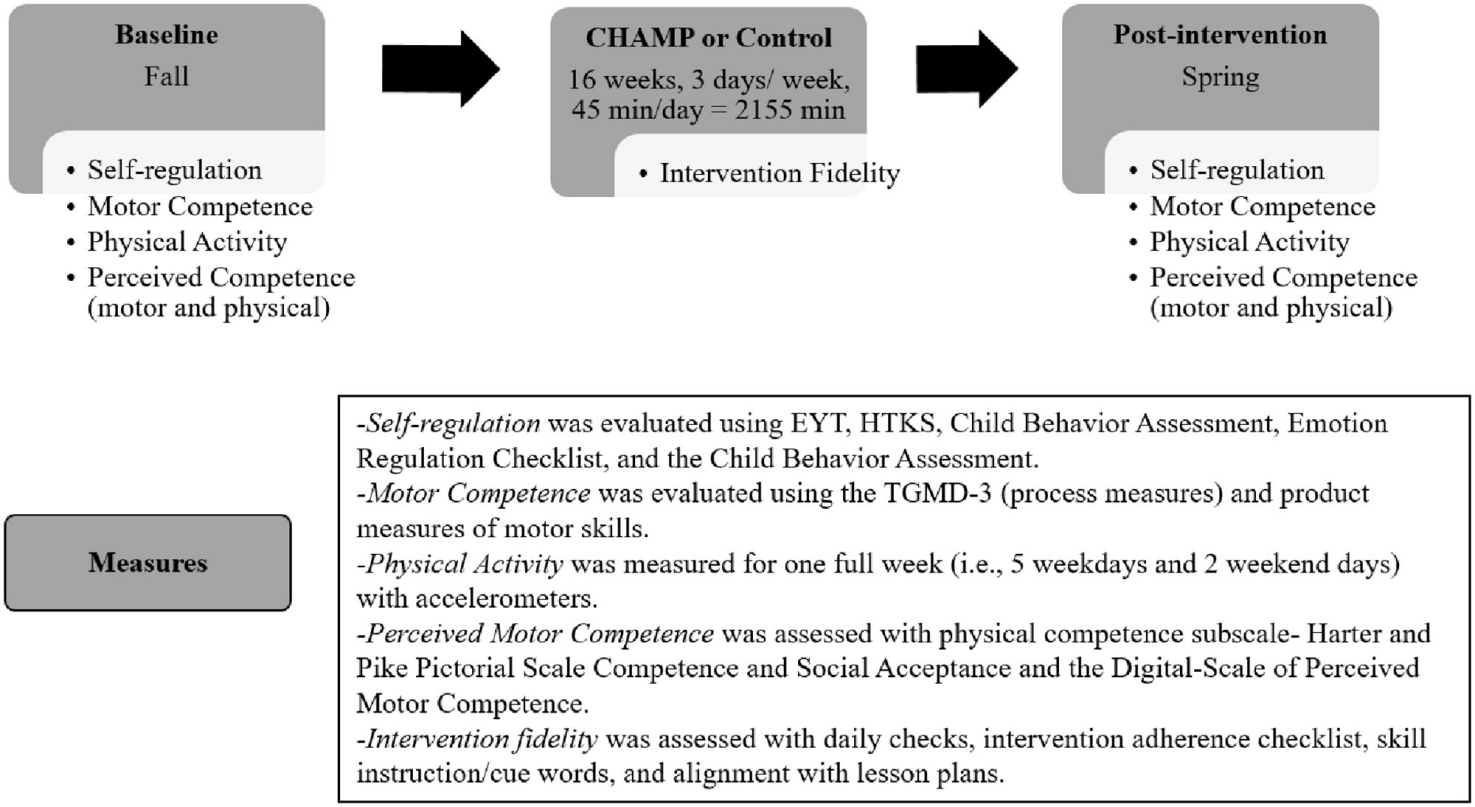
PATH-SR study timeline.

The Promoting Activity and Trajectory of Health (PATH)–Self Regulation (SR) cluster RCT is a federally funded supplemental award from the National Institutes of Health (NIH) Common Fund which expands upon an ongoing RCT, A PATH (Promoting Activity and Trajectories of Health) for Children that is funded by the National Heart, Lung and Blood Institute (NHLBI; R01-HL-132979). The protocol paper for the PATH for Children has been published [69]. The Institutional Review Board at the University of Michigan approved the PATH-SR study (HUM00133319), and the RCT is registered in the Clinical Trials Registry NCT03189862. Informed written consent will be obtained from children’s parent/guardian(s) along with verbal assent from each child. The reporting of this research will follow the recommendations of the Standard Protocol Items: Recommendations for Interventional Trials (SPIRIT) [70]. Fig 1 depicts the SPIRIT diagram for the schedule of enrollment, interventions, and assessments and Fig 2 is the PATH-SR Study timeline for the 16-week intervention study during a preschool school-year.

### Study context

The study will take place in two federally funded early learning centers located in the Midwestern United States. The centers provide free quality preschool programs to children who come from a household with an income that is at least 100% below the federal poverty level. 120 children between the ages of 3.5 to 5 years of age will be randomized to a mastery-climate motor skills intervention (n = 70) or control (n = 50) condition. In center 1, 7 classrooms expressed interest in participating and in center 2, 6 classrooms were interested. Randomization will occur at the level of the classroom. Specifically, classrooms will be cluster-randomized to receive either the mastery-climate motor skills intervention (i.e., CHAMP; treatment) or the control condition (i.e., outdoor recess) using computer-generated random numbers. A total of 7 classes from both centers were assigned to CHAMP and the remaining 6 were assigned to the control. Unfortunately, due to constraints at the Head Start centers (i.e., uneven number of classrooms to recruit and teachers’ interest in participating), there was not an equal number of classes to distribute evenly between treatment and control.

### Participants and protocol

#### Inclusion/Exclusion criteria

Preschoolers ≥ 3.5 to 5.11 years old are eligible to enroll and participate in this study. Children are ineligible if any of the following apply: exhibit characteristics or diagnosed with syndromes or diseases that would affect participation in the motor skills intervention and/or exhibit characteristics or had a previous diagnosis of any major illness, developmental, and/or physical disability since birth. If a child is deemed ineligible due to an above condition, but have parental consent, they will be able to participate in the treatment but no data will be collected on these individuals.

#### Recruitment

After receiving human subjects IRB approval, the following procedures will occur regarding participant recruitment and the informed consent process. Parent(s)/guardian(s) will receive an information letter from the Principal Investigator notifying them of the PATH-SR Study at the beginning of the school year. The letter will provide a brief description of the study along with a statement from the school administrators indicating that parent(s) are not obligated to participate. Members of the research team will be present during morning drop-offs and afternoon pick-ups to answer any questions from parents/guardians. All parents/guardians who return a consent form, regardless of whether they agreed to participate in the PATH-SR Study, will receive a one-time cash incentive of $5.00. In addition to parental consent, verbal assent will be obtained from each preschooler. Parents will receive reminder letters for each upcoming PATH-SR assessment and a developmental report of their child’s findings from each assessment (i.e., motor skills, physical activity, health outcomes, SR outcomes). Each center will be provided with aggregated data of the findings.

### Children’s Health Activity Motor Program (CHAMP)

The intervention that will be used in this study will be the Children’s Health Activity Motor Program (CHAMP). CHAMP is an established motor skill intervention created using Achievement Goal Theory and delivered as a mastery-motivational climate. This approach encourages children to learn and develop new skills, increase their level of competence, and achieve a sense of motor skill mastery-based on their perceptions. Over 14 years of preliminary work supports the effectiveness of CHAMP in improving motor skill performance [40, 47, 48, 62–67], increasing physical activity [49, 67, 68], enhancing perceived physical/motor competence [47, 48, 63], and maintaining delay of gratification [40] in preschool- and/or school-age children. Due to the theoretical underpinnings and implementation of the intervention, CHAMP provides an innovative approach to potentially improve SR in young children. Table 2 provides an overview of the core tenants of CHAMP and details on the theoretical principles and intervention implementation (both instructor training, child engagement, and fidelity) that have the potential to support SR in this population are discussed below.

**Table 2:** Core tenants of CHAMP.

Core theoretical principles	Grounded in Achievement Goal Theory Encourage children to adopt a mastery-orientation through creating a mastery-motivational climate (e.g., implementing TARGET structure)
Core constructs	High-autonomy learning climate (multiple stations and multiple levels of difficulty within stations) Shared decision making between instructors and students Motor skill instruction using proper cue words, modeling, and developmentally appropriate pedagogy and activities
Core instructional principles	Shared decision making between instructors and students Provides motor skill instruction using proper cue words, modeling, and developmentally appropriate pedagogy Recognize individual levels of abilities and progress Evaluate student performance based on self-referenced not norm-referenced standards
Core instructional practices/pedagogies of CHAMP instructors	Set up CHAMP session with various level of difficulty within each of the 3–4 stations Introduce and teach each motor skill to whole group using proper cue words and modeling Provide individual recognition and evaluation during the autonomy-motor skills station Instruction during autonomy-motor skill stations can range from verbal correction, modeling, to physical manipulation. All praise is delivered privately
Expectations of child in navigating CHAMP	To participate in large group activities during start and end of class To self-navigate and engage in motor skill practice during autonomy-motor skill stations To create and curate self-selected peer groups

#### Theoretical principles

CHAMP is grounded in Achievement Goal Theory. This theory originates from educational psychology and focuses on the learners’ motivation to learn [41, 43, 44], since goals for learning influence intrinsic motivation. Achievement Goal Theory refers to the beliefs, attributions, and affect that contribute to one’s behaviors and represents how an individual approaches, engages, and responds to various activities [42, 43]. Individuals can take either a mastery- (task-) or performance- (ego-) orientation [42, 71]. Performance- (ego-) individuals focus on ensuring that their performance is successful and superior to others while mastery- (task-) individuals engage in learning for the sake of learning and are less threatened by failure. Mastery- (task-) oriented individuals often have higher intrinsic motivation [42, 43] exhibit an intrinsic interest in learning [44, 72] and have positive attitudes towards learning [72, 73].

Learning environments can be intentionally and purposefully structured to encourage learners to adopt a mastery- (task-) orientation to learning. These environment are called mastery-motivational climates and are created using Epstein’s TARGET structures [46] (see Table 1). Applying the TARGET structures to the learning environment redistributes the ownership to the learner and allows them to autonomy to navigate the intervention climate, make their own choices on how, where, and in what level of difficulty they engage in and practice new skills. In the case of CHAMP, allowing the child the autonomy to navigate the mastery-motivational climate fosters self-navigated engagement, self-selections of learning groups, and self-paced learning. These activities and expectations require a child to demonstrate and continually practice the cognitive, emotional and behavioral regulation skills that are hallmarks of effective SR (see Table 1). Therefore, even while the primary learning outcomes of CHAMP are motor skills and health behaviors, the theoretical principles (i.e., achievement-goal theory) and implementation (i.e., TARGET structures) applied during the program are likely to promote SR in children.

#### Intervention session design

The CHAMP motor skills intervention will be delivered across a dose of 2,155 minutes. The intervention will be completed 3 days per week across 19 weeks (i.e., 16 weeks of the CHAMP intervention plus 3 weeks in between for winter and spring breaks) in an academic school year. Each 45-minute CHAMP session will consist of three parts: (a) 3–5 min of introductory activity, (b) 35–38 min of motor skill instruction and practice delivered as a mastery-motivational climate, and (c) 3–5 min motor skill closure activity and review. Each CHAMP session will include 3–4 motor skill stations that will rotate across 15 different motor skills. The stations will all be designed using a “slanted rope effect” to allow a range of difficulty in practice and engagement in the stations that ranges from easy to difficult. All stations and the ability to manipulate difficulty of engagement within each stations will be taught to the children during the introductory activity, and then during 35–38 minutes of motor skill instructions and practice children will self-navigate through the activities. During this time, instructors move about the intervention and encourage children to continually engage in the intervention as well as will provide individualized feedback and instruction in accordance with the TARGET structures. Instructors provide feedback on motor skill performance using best pedagogical practices including cue words, modeling, and physical manipulation. For example, instructors may notice a child is not stepping contralaterally during throwing. They will recognize this individual performance and encourage the child to engage in better throwing performances by verbal prompts (e.g., “step with the other foot”), modeling (e.g., “watch me! Step like *this*”), external cues (e.g., sticker on the foot they should step forward with), or physical manipulation (e.g., picking up and moving the correct foot). Once the child changes their movement pattern and steps forward with the contralater foot, the instructor will evaluate this change in performance based on self-referenced norms and praise the child for their individual change in performance.

While children and instructors share responsibility for the authority of the CHAMP sessions, children should engage in the session. During the introductory activity and wrap-up and review, children are asked to sit in a circle in the middle of the classroom and listen to the instructor explain the activities/stations of the day (introductory activity) or recap the daily activitys (wrap up and review). During the 35–38 min of motor skill instruction and practice delivered as a mastery-motivational climate children are granted autonomy to move around the space as they desire. Ultimately, where they engage, how long they engage, what level of difficulty they engage with, and their peer structures during practice are decided by the children with continued encouragement and re-direction from instructors. For example, a child could elect to spend the entire 35–38 minutes practicing their throws at a moderate level of difficulty by themselves. Alternatively, a child could elect to only spend 3 minutes at throwing at an easy level of difficulty then move to running with a group of three to four peers. Both examples constitute “successfully” engaging in CHAMP and require children to manage their emotions, focus attention, persist, plan, and evaluate their actions in order to improve their motor competence and perceived motor competence and enhance their engagement in PA [41, 42, 46]. All these behaviors are linked with SR which support the potential of CHAMP to improve SR in this population.

#### Intervention instructors

CHAMP will be implemented by two motor development researchers who are Ph.D. students. The lead instructor has 6 years of experience implementing the CHAMP intervention and was involved in the development of the program. The second instructor has a degree in physical education and a background in motor intervention implementation. Additional research personnel (n = 1–2) will be present to assist with other managerial tasks for the intervention (e.g., ensure that the cameras are recording, record attendance, equipment set-up and breakdown, collecting and returning of children to classroom, fidelity checks, etc.).

All research personnel will undergo training before the start of the intervention. The training takes approximately 40 hours to complete. The training will include readings and discussion on (a) Achievement Goal Theory and mastery climates in general and in regards to movement interventions, (b) cue words best practices in motor skill instruction and feedback, and (c) best pedagogical practices for preschool-aged children. All lead instructors must undergo additional training whereby they will watch three previously recorded instructional sessions of the CHAMP intervention and discuss how achievement goal theory and the TARGET structures were implemented in the intervention. Lastly, each instructor will complete a mock CHAMP session and practice station set up, skill and CHAMP instruction, individual feedback and recognition on motor performance, and CHAMP closing. Both the reviewed videos and the mock CHAMP session will be completed under the direction of the lead author and creator of CHAMP (LER), and instructors have to demonstrate 100% fidelity with CHAMP and TARGET structures prior to the start of the intervention.

#### Intervention fidelity

Fidelity checks on the TARGET structures and instruction will also be completed at every session to ensure the intervention adheres to the TARGET protocol. For the past 14 years, the following fidelity checks have been used to ensure the extent to which the CHAMP intervention is implemented as intended [40, 47–49, 62–68]. Daily checks will be completed to ensure the dose, adherence, quality of delivery, intervention alignment with core constructs and intervention protocol, and etc. All intervention sessions will be digitally recorded to enable future reviews of each session if needed. For each session, a research staff member (i.e., not the CHAMP instructors) will complete the following fidelity checks that address dose, instruction, and TARGET stuctures. For dose, the checks will record the start and finish of each session (i.e., to calculate the total minutes of the intervention session), the amount of time devoted to skill instruction and demonstration, and the amount of time children engaged in the practice of motor skills. Research staff members will evaluate the instruction provided during each CHAMP session to ensure that it aligns with the pre-determined CHAMP lesson plan. Specifically, the checks will ensure that clear instruction for each motor activity station are provided, the use of the provided critical cue words and that an accurate demonstration, ensure that instructors check for student understanding, compliance with the CHAMP lesson plan, and if modified a description of the deviation will be recorded. These instructional checks will also record the type of feedback (specific, corrective, and/or evaluative) provided to the child, along with the use of manual manipulation to aid motor skill learning. Finally, the TARGET structures will be used to ensure that there are three motor skills activity with 3–4 levels of task difficulty present (Task), children have the opportunity to independently choose their engagement in the session (Authority), feedback focuses on progress, effort, and improvement (Recognition and Evaluation), children have the option/choice to work in small group, with peers, or individually (Grouping), and lastly the 35–38 minutes of motor skill instruction and practice was self-paced based on the individual child’s level of engagement (Time). As noted in the intervention training section, two, PhD instructors will serve as the interventionist for this study and will be trained by the PI of the project. In addition to each session being digitally recorded, instructors will wear wireless microphones to aid in assessing the intervention fidelity. Instructors will receive feedback regarding their instruction weekly. Fidelity checks on the TARGET structures and instruction will also be completed at every session to ensure the intervention adheres to the TARGET protocol.

#### Control condition

The outdoor recess/free play program is the early learning centers’ current motor program for accreditation and will serve as the control condition for this study. Outdoor recess/free play will be implemented according to the existing procedures within the centers. Each class will receive two, 30–45 min outdoor recess (free play) periods each day. For this study, the control group will receive two, 30–45 min per day outdoor sessions, whereas the treatment (CHAMP) group will receive one, 30–45 min outdoor session each day after their nap, as the morning recess session was replaced with the CHAMP intervention on days the intervention will be implemented. The centers’ outdoor programs consist of outdoor free-play activities on a large playground area with a variety of play structures (e.g., swings, slides, ladders) that promote physical activity, gross motor skills, balance/stability, and movement skills. No planned instruction or activities will be provided to the preschoolers during outdoor recess. Classroom teachers and the research personnel will be asked to confirm that the daily outdoor recess sessions were completed with a check-off sheet.

### Measures

Data will be collected by a trained research team. Outcome measures will be collected for all participants in both the treatment and control groups at pre-test (i.e., before the start of the intervention) and post-test (i.e., at the conclusion of the intervention). Pre-test measures will occur in September/October and post-tests will occur in late April/May. On average, we anticipate it will take three, 25–30-minute sessions across three days to complete all the assessments. This time was based on the allotted time provided by the preschools and the average time preschoolers’ tends to stay focused on these assessments from previous studies. All perceived motor competence data will be collected before children complete motor skill assessments. The order of completion will be as follows: Session 1: anthropometrics (e.g., height, weight, waist circumference; less than 5 minutes), perceived motor competence (5–8 minutes), and SR (17–20 minutes); Session 2 and 3: motor skills (30 minutes). Session 4: any measures that were not completed in Session 1–3, mainly motor competence and SR, was completed during Session 4 (30 minutes). If additional time was needed to complete the preschoolers’ assessment, the research team reached out to the classroom teachers directly to arrange for a time to complete the assessment(s).

### SR Measures

SR will be assessed using a series of computer-based and interactional behavioral tasks, teacher reports, and observer reports. SR constructs measured will include cognitive SR (cognitive flexibility, working memory, attention shifting), behavioral SR (behavioral inhibition), and emotional SR (emotion regulation).

*Cognitive SR* will be measured with cognitive flexibility and working memory using the Early Years Toolbox (EYT), a normed collection of iPad-based assessments for preschool-aged children [74]. All assessments will be administered on an iPad and data will be automatically stored in a secure online repository. The EYT is a developmentally sensitive measure of executive function in young children [74]. Cognitive flexibility will be assessed using the EYT “Boats & Rabbits” game. In this game, children will be presented with stimuli at a boat bifurcation and instructed to sort the stimuli into the correct castle at the end of each moat. Stimuli are sorted based on shape (rabbits vs. boats) or color (red vs. blue). The child is first asked to sort along one dimension (e.g., shape), then to switch and sort along the other dimension (e.g., color), then to short by either dimension depending on whether the stimulus is presented inside a black border. Thus, the game includes a total of three series: “pre-switch”, “post-switch”, and “border task”. Each series includes two practice trials and six test trials. Children will receive a point for each correctly sorted stimulus. Children must receive at least 5 points in both the pre- and post-switch series to progress into the border task series. The total number of correctly sorted stimuli after the switch (i.e., across the “switch” and “border task” series; range: 0–12) represents the ability of a child to flexibly shift attention and will be collected as the primary outcome variable indicating cognitive flexibility.

Visual-spatial working memory will be assessed using the “Mr. Ant” game from the EYT. In this game, children are presented with a cartoon ant figure who “puts on” stickers on different parts of his body. Children will be required to remember the location of the stickers and put them back on Mr. Ant. In each trial, children will see Mr. Ant with stickers for 5 seconds, followed by a blank screen for 4 seconds. Then, they will see an image of Mr. Ant without stickers and will be verbally prompted to recall and place the stickers back on Mr. Ant. The game includes eight levels each with three trials. The task is progressive and ranges from one sticker presented in level 1 to nine stickers presented in level 8. Children must correctly place the stickers in at least one of the three trials to progress to the next level, and children advance through the game until they fail to get all three trials correct on the level. Both overall points and trial accuracy are recorded. Children receive 1 point for each level they complete with at least two correct trials and receive 1/3 of a point when they only complete one correct trial. Total number of earned points will be summed across the assessment and trial accuracy will measured as the total number of correct trials completed across the assessment. Both the total number of earned points as well as accuracy will be recorded as primary outcome variables.

*Behavioral SR* will be measured with the Head-Toes-Knees-Shoulders Task (HTKS) and observed SR. The HTKS is a developmentally appropriate measure to assess behavioral inhibition in young children [75–77]. The HTKS taps into three underlying and simultaneous mechanisms: (1) memory, (2) cognitive flexibility and (3) inhibition within a behavioral setting/outcome [76]. In the task, children will be instructed to touch the body part opposite to what the administrator says. For example, children are given the instruction “Touch your head” and must touch their toes. Children will have to remember the rules of the game, inhibit their initial reaction, and change their response to the opposite of the verbal instruction to successfully complete the tasks. The assessment includes three parts. Each part includes verbal instructions and between 4–6 practice trials preceding the 10 test trials. All practice and test trials will be scored from 0–2 points. Children receive a 2 if they successfully complete the trial, a 1 if they correct themselves during a trial, or a 0 if they fail to complete the trial correctly. Children only advance to the subsequent part if they receive at least 4 points during the test trials. The total number of points earned in test trials will serve as the primary outcome variable. All HTKS trials will be coded live. All coders will undergo a 5-hour training and establish inter-rater reliability of 90% prior to the start of data collection.

Observed SR will be assessed with the Child Behavior Assessment. This assessment will be completed by a member of the research team immediately following each data collection session and will be used to measure child engagement and compliance during SR testing. The goal of this overall behavior rating is to capture children’s global behavioral responses across a series of SR tasks. The Child Behavior Assessment included 10-items selected from the 28-item Preschool Self-Regulation Assessment Assessor Report [78]. Example questions include: “Lets examiner finish before starting task; does not interrupt” or “Child has difficulty waiting between tasks.” Scale items reflect child responses across tasks and are each rated on a scale from 0 indicating a low degree of SR with regard to the item (e.g., child impulsive throughout assessment, needed lots of boundary-setting) to 3 indicating a high degree of observed SR with regard to that item (e.g., child waits before pointing to materials, reaching for blocks), resulting in a score that reflects the degree to which the child consistently demonstrated self-regulation, across tasks. A single average scale score is recorded (Cronbach’s α = 0.71).

*Emotional SR* will be assessed using both the Emotion Regulation Checklist [79]. The Emotion Regulation Checklist will be used to score children’s emotional regulation overall. This checklist is a valid and reliable 24-item questionnaire used to assess young children’s emotional regulation (Cronbach’s α = .83) and negative lability (α = .96; total scale score α = 0.89). Negative lability questions are scored so that a higher score reflects greater negative affect. Emotion regulation questions are scored so that a higher score reflects better emotional regulation. Negative lability and emotional regulation subscales are calculated to reflect average scores and will be the primary outcome variables. The Emotion Regulation Checklist will be completed by the classroom teachers. Classroom teachers will be paid for their services/role on this project as study reporters on child outcomes and will be trained in the administration of the Emotion Regulation Checklist.

### Health behaviors

*Motor Competence* will be evaluated using process measures of motor skills at pre-test and post-test. The Test of Gross Motor Development-3^rd^ edition (TGMD-3) assesses process measures of motor skills [80]. The TGMD-3 is a valid and reliable criterion-based assessment used to measure fundamental motor skills in children ages 3 to 10 years. It consists of six locomotor (run, jump, gallop, slide, hop, and skip; Cronbach’s α = .88) and seven ball skills (throw, catch, dribble, underhand throw, kick, one-handed forearm strike, and two-handed strike off a tee; Cronbach’s α = .93). Each motor skills is divided into 3–5 specific performance criteria and a child will receive a 1 if they perform the skill correct and a 0 if they fail to perform the criteria. Children will complete three trials for each skill; one practice trial and two scored trials. The TGMD-3 will be completed according to the test manual and procedures and children will receive a digital demonstration of the skills before the practice trial [81]. If the child did not understand the motor skill during the practice trial a second demonstration will be provided. The child wil then completed the two test trials. The identical verbal instructions will be provided in both the digital and live demonstration. The TGMD-3 assessments will be digitally recorded and coded by a motor development expert, who will serve as an external consultant to the project and is blind to the randomization. Raw scores for the two TGMD-3 subscales, locomotor (0–46) and ball skills (0–54), will be summed to derive the total score (0–100) that will be used for data analyses. Inter-rater reliability will be established between consultant/coder and two members of the research team on a random selection of 30% of the assessments and will be completed every year.

*Physical Activity* will be measured with ActiGraph accelerometers (model wGT3X-BT; Actigraph, Pensacola, FL, USA) secured by a hospital band on participants’ non-dominant wrist for one full week (i.e., 5 weekdays and 2 weekend days) at pre-test and post-test. The devices will be placed on the child during the school day and will be set to start recording at midnight. The devices will be removed after seven full days of recording. The devices will be set to collect data at 30 hz, the standard frequency used with accelerometers. Time spent in intensity categories will be based on vector magnitude minus the value of gravity (g) (i.e., (x^2^ + y^2^ + z^2^)^1/2^–1) referred to as ENMO (Euclidean norm minus one). The primary outcome will be minutes in MVPA per day but additional measures of physical activity will be analyzed based on the current physical activity recommendations [82, 83]. Hildebrand cut points will be applied to activity data [84, 85]. with MVPA defined as activity over 201 mg. To be considered valid wear, participants need to have at least 12 hours of valid accelerometry data per day for at least 4 days (3 weekdays and 1 weekend day) [86, 87]. Non-wear time is defined when either the standard deviation (SD) is less than 13 mg for two of the three axes or when the value range of each accelerometer axis is less than 150 mg, calculated for moving windows of 60 minutes with 15-minute increments [88]. The following steps will be taken to aid with device compliance 1) a letter to the parents which explains placement and provides a simple diagram, 2) physically show the parent and teachers how to place the accelerometer on the child if needed (both teachers and parents will be provided with spare bands), 3) text messages, phone calls, and flyers as prompts and reminders, 4) research staff will check the placement of accelerometers each day of data collection and 5) an incentive gift card ($10) upon the return of the device.

*Perceived competence* will be assessed with the Harter and Pike Pictorial Scale of Perceived Competence and Social Acceptance—physical competence subscale [89, 90] and the Digital-Scale of Perceived Motor Competence [91, 92] at pre-test and post-test. The physical competence subscale of the Pictorial Scale of Perceived Competence and Social Acceptance measures children’s global perceived physical competence and consists of six items (swinging, climbing, tying shoes, running, hopping, skipping) that are presented in static pictures [89, 90]. Mean reliability coefficients (α) range from 0.66–0.89 and the reliability for the physical competence subscale is 0.66 [89, 90]. The appropriate scale (i.e., gender and ethnicity) will be used for each child.

The Digital-Scale of Perceived Motor Competence is a digital-based assessment that measures perceived motor competence and allows individuals to see the entire motor skill executed as a video rather than a static picture [91]. The scale uses a similarly two part bifurcation selection process as the Pictorial Scale of Perceived Competence and Social Acceptance; however, the DSPMC uses an adult model and includes the fundamental motor skills of the TGMD-2. Face validity of the DSPMC has been established, and research supports the DSPMC has acceptable validity and reliability both preschool (α = 0.78; ICC = 0.84; 95% CI = 0.76–0.89) [92] and elementary-aged children (α = 0.78; ICC 0.80; 95% CI = 0.76–0.894) [91].

For both assessments, children (1) select the picture/video that is most like him or her (a competent/skilled or not competent/skilled) and (2) focus on the picture/video and indicate whether the picture/video was just a “little bit” or “a lot” like them. The range of scores for each item on the subscale is 1 (low competence) to 4 (high competence). Both assessments are established tools and standardized test protocols will be used [89, 91]. For analysis, these two measures will be examined separately since one is a measure of perceived physical competence and the other is a measure of perceived motor competence.

### Health outcomes

Waist circumference and body mass index will be measured according to standard procedures [93, 94] at pre-test and post-test. Waist circumference will be measured with a non-elastic tape (Seca 201; Seca North America. Chino, CA. United States) at the umbilicus [95]. The measurement will be taken as the children completes a breath (i.e., exhales). Height will be measured to the nearest unit in bare feet with the child standing upright against a portable stadiometer (Charder HM200P PortStad; Taiwan R.O.C). Weight will be measured to the nearest unit with heavy clothes removed (i.e., wearing pants and shirt) using a portable electric weight scale (Seca 813; Seca North America. Chino, CA. United States). All scales will be calibrated before testing. BMI will be calculated based on age- and sex-specific CDC growth charts. BMI will be transformed into BMI z-scores for analyses. Inter- and intra-rater reliability of data collection staff will be assessed at baseline data collection and monitored throughout data collection.

### Statistical analysis plans, power considerations, and data management

#### Overall analysis plans

We will apply transformations to assure normality, run descriptive statistics, and assess potential covariates to include. While maximum effort will be made to retain all participants and minimize the amount of missing data, we anticipate there will be some data lost-to-follow-up and incomplete measures. Therefore, we will address missing data in the analysis plan by applying advanced statistical techniques, such as “multiple imputations” using PROC MI in SAS and IVEWARE SAS macro. Our overall approach will be to employ multivariate analysis to assess associations among key variables using the appropriate models based on the distribution of the data (i.e., normal, categorical data, count data, reaction time data). Since this is a cluster-randomized trial, adjusting the covariates (e.g., child sex, age, race/ethnicity) will aid in controlling additional unbalancesess due to the limited sample size. All analysis will be done using SAS 9.3 or R 4.1.0 [96–98]. Additionally, we will perform “intent to treat” analysis and assume all subjects comply to the assigned group. Findings/results from the study will also following the CONSORT guidelines.

#### Power considerations

When we calculate the sample size and power, we assumed the intra-cluster correlation is 0.3. The achievable power is calculated for a detectable effect size, which is the detectable mean difference standardized by the square root of sample variance. Given the randomization design with 70 children in the intervention group and 50 children in the control group, we have 80% power to detect a difference in cognitive SR, behavioral SR, or emotional SR with an effect size of 0.52 and a Type I error level of 0.05.

#### Specific analysis plan for Aim 1

Examine the immediate (pre- to post-test) intervention effects of CHAMP (compared to control participants) on cognitive SR (cognitive flexibility, working memory, attention shifting), behavioral SR (behavioral inhibition), and emotional SR (emotion regulation). The immediate post-intervention effect of CHAMP (compared to control participants) on each SR outcome variable will be evaluated at post-intervention. We will examine descriptive statistics for both pre-and post-intervention SR outcome variables for each group. The change in cognitive SR, behavioral SR, and emotional SR scores will be compared between the intervention (CHAMP) and control groups using regression models, adjusting for other confounding factors. We assume randomization will be successful and we will monitor throughout the trial. It is always good practice to monitor along the way and we will do so, but randomization may be imperfect as this is a randomized cluster trial taking place in the real word (i.e., Head Start setting). If randomization does not work this will lead to biased results and methodology, our planned analyses to adjust other variables will be considered. Random effects will be included in the model to accommodate for the potential within-cluster correlations due to nested classroom data. We anticipate that some children will have only partial adherence to the intervention (i.e., attend a subset of sessions), thus we will also conduct a dose-response analysis where dose corresponds to number of sessions. We will investigate the amount of attrition from pre-to post-intervention, and attempt to identify baseline (or pre-intervention) predictors of dropping out. The information will indicate possible bias in the change estimates.

#### Specific analysis plan for Aim 2

Examine the associations between SR (cognitive SR, behavioral SR, and emotional SR) and changes in health behaviors (motor competence, perceived competence, physical activity) and health outcomes (body mass index, waist circumference). We will further evaluate the associations between changes in SR and changes in children’s health behaviors and outcomes, using regression models. More specifically, we will examine the strength of association between each SR variable and our outcomes of interest using bivariate analyses to compare change in SR to change in motor competence, perceived competence, and PA (using an alpha value of p < .05). To test whether the strength of association varies by intervention status, we will use multivariate regression models (controlling for covariates as needed) to examine the association of SR and outcomes in each group (CHAMP and control). Similarly, random effects will be included in the model to accommodate for the potential within-cluster correlations from nested classroom data. We will further apply Structural Equation Models (SEM) to evaluate whether the CHAMP intervention has a causal effect on children’s health outcomes mediated through SR.

#### Data management

Extreme care to ensure high-quality and secure data will be exercised. All data will be stored securely at the University of Michigan. All data will have only a numerical identifier so that individual respondents, except for video data, cannot be identified. All data will be reported as aggregate statistics and no individuals will be recognizable from the data reported. All data will be scanned for consistency, errors of omission, and appropriateness of the response, and 30% of data will be checked by a blinded member of the research team. Once a coded and cleaned data file has been prepared, frequency distributions and descriptive statistics (means, standard deviations, and ranges) for each of the measured variables will be used for consistency checks and to verify the comparability of the groups. Logic check programs will be run to ensure that each data point falls within the expected range or corresponds to possible values in the codebook. These tracking system files will be maintained on a secure server at the University of Michigan. Data will be analyzed using SAS 9.3 or R 4.1.0 [96–99]. All members of the study team will be required to complete the web-based National Institutes of Health University of Michigan Responsible Conduct of Research Training Program. The investigative team will engage in ongoing data management training, data monitoring, and measurement training over the course of the investigation. Rewards and incentives will be incorporated after each assessment time point to aid in participant engagement.

## Discussion

SR is an important domain of early child development that plays a foundational role in promoting well-being across the lifespan [4, 5], including emotional adjustment, social functioning, and educational achievement [9, 10]. Recently, motor skills have been linked to SR. Becker, Miao, Duncan and McClelland [39] found that fine motor skills were related to working memory and behavioral SR while Robinson, Palmer and Bub [40] later found that a mastery-based motor skills intervention helped maintained preschoolers’ delayed gratification, but the work in this area is limited. This study seeks to examine the immediate (pre- to post-test) intervention effects of a mastery-based motor skills intervention, CHAMP on child SR (cognitive SR, behavioral SR, and emotional SR). This study also seeks to examine associations between SR and changes in health behaviors (motor competence, perceived competence, physical activity) and health outcomes (waist circumference, body mass index).

The research that explores SR from a movement perspective is relatively sparse. To our knowledge, no studies have tested the effects of a mastery climate, motor-based intervention on child SR measures. Findings from the study could potentially provide new knowledge as it relates to mastery-climate, motor-based intervention in early childhood settings and their contribution to the social and emotional development of young children along with the physical development. This proposed work is innovative in two ways. Specifically, this study will explore if a mastery-based motor skills intervention could enhance child SR. This intervention approach is not commonly used to promote SR in the child development literature. Secondly, this study will also study SR in the context of health behaviors and health outcomes. Interventions have shown to improve SR, but studies have not examined whether improving SR in children affects their health behaviors and/or health outcomes. This proposed study will expand the literature since little prior work has explored connections between SR and health constructs from a movement perspective.

Additionally, most studies have established associations between motor interventions and outcomes to SR, but few have been experimental. Suppose this intervention proves to be effective in enhancing child SR. In that case, critical elements related to implementing a mastery-climate school-based motor skills intervention to promote SR will be identified. The new knowledge from this study could be used from an educational standpoint by classroom and/or physical education teachers as an intervention approach to promote motor skills and SR in young children. This study could also offer important insights into potential avenues for preventive interventions across a range of health behaviors. Eventually, the feasibility of disseminating and implementing the CHAMP intervention to support SR, health behaviors, and health outcomes could be scaled up to impact more children.

## Supporting information

S1 Checklist (DOC)

S1 File (DOCX)

S2 File (PDF)

S3 File (PDF)

## Abbreviations

CHAMP: Children’s Health Activity Motor Program
PATH: Promoting Activity and Trajectories of Health
