# Supplementary material for: Protocol for a cluster randomized clinical trial of a mastery-climate motor skills intervention, Children’s Health Activity and Motor Program (CHAMP), on self-regulation in preschoolers
Source: PLoS One. 2023 Mar 9;18(3):e0282199. doi: 10.1371/journal.pone.0282199 (PMC9997967; doi:10.1371/journal.pone.0282199)
Supplement: S1 File — (DOCX) [file pone.0282199.s002.docx]

**IRB Research Plan - HUM00133319**

A PATH (Promoting Activity and Trajectories of Health) for Children (Phase I, Cohort 2)

Science for Behavior Change

(Based on PROTOCOL HUM0011789)

**Ame00094488**

The Amendment to HUM00133319 are as follows:

1. Update of Dr. Elizabeth Webster affiliation

Objective

- 1. Objective 1

Physical inactivity in children is a major public health risk factor and national health objective. This National Institutes of Health application 1 R01 HL132979-01 entitled “A PATH (Promoting Activity and Trajectories of Health) for Children” (scored at the 13^th^ percentile) will investigate the immediate and long-term effects of a motor skills - the Children’s Health Activity Motor Program (CHAMP) on motor competence, perceived motor competence, and physical activity in preschool-age children. The specific aims are to: 1) examine the immediate post-intervention effect of CHAMP (compared to control participants) on motor competence, perceived motor competence, and physical activity in preschool-age children; 2) assess the sustainable effect of CHAMP (compared to control participants) on motor competence, perceived motor competence, and physical activity across middle childhood; and 3) examine and compare the immediate and long-term mediating effects of perceived motor competence on the relationship between motor competence and physical activity in preschool-age children. We hypothesize that children in CHAMP, compared to control, will demonstrate higher levels of motor competence, perceived motor competence, and physical activity at post-intervention and across the 3-year follow-up. This study will consist of a cluster randomized control trial and CHAMP will be implemented in a predominantly minority and low income population, Head Start preschoolers (N = 300; 3.5-5 years old). Thirty classes of preschool children will be randomly assigned to either the treatment (CHAMP, n = 15) or control (normal preschool free-play/recess, n = 15) condition. The CHAMP intervention will be implemented for (4, 30-minute classes a week or 3, 40-minute classes a week, based on the needs of the school schedule)~~. 30 minutes/day 4 days per week for 30 weeks (≈3600 min dose)~~. Data will be collected on: a) 7-day physical activity levels using accelerometry, b) process- and product-oriented motor skill competence c) perceived motor competence.

- 1. **Specific Aim/Hypothesis**

**Aim 1: Examine the immediate post-intervention effect of CHAMP (compared to control participants) on MC, PMC, and PA in preschool-age children.**

**Hypothesis 1:** Children in CHAMP, compared to control, will demonstrate higher levels of MC, PMC and PA at post-intervention.

**Aim 2: Assess the sustainable effect of CHAMP (compared to control participants) on MC, PMC, and PA across middle childhood.**

**Hypothesis 2:** Children in CHAMP, compared to control, will demonstrate higher levels of MC, PMC and daily PA across the 3 yr follow-up (middle childhood) period (sustainable).

**Aim 3: Examine and compare the immediate and long-term mediating effect of PMC on the relationship between MC and PA in preschool-age children in the CHAMP and control.**

**Hypothesis 3:** PMC will not mediate the relationship between MC and PA at baseline. PMC will mediate the relationship between MC and PA immediately post intervention and across the 3-yr follow-up period.

**Impact.** Positive findings will support the development of early childhood motor competence and physical activity programs that promote positive and sustainable physical activity behaviors that contribute to healthy growth and development.

1. **Objective 2.** Cohort 2 participants will not partake in Objective 2.

A secondary aim of the project (i.e., funded NIH supplement - FOA PA-16-334, *Science of Behavior Change)* is to examine the immediate (pre- to post-test) effects of the CHAMP intervention on SR, and associations between SR and changes in motor competence, perceived motor competence, and PA. SR will be assayed using a multi-method approach, including direct assessment of child behavioral and attentional regulation, computer-based assessment of cognitive control strategies, and teacher-reported emotional regulation.

The specific aims for this Supplement will be tested in 120 preschool-aged children (3.5-5 years) who are attending Head Start and receiving CHAMP (n = 10 classrooms) or an active free-play/recess control condition (n = 10 classrooms).

b. **Specific Aim/Hypothesis**

**Aim 1**. Test the hypothesis that CHAMP improves child SR (*cognitive flexibility, working memory, behavioral inhibition, emotion regulation*) from pre- to post-test, compared to control.

**Aim 2.** Test the hypothesis that improved SR (*cognitive flexibility, working memory, behavioral inhibition, emotion regulation*) is associated with improved motor competence, perceived motor competence, and objectively-measured PA from pre- to post-test.

**Impact.** From an educational perspectives, positive findings will provide initial support for CHAMP as an effective intervention to enhance self-regulation and contribute to healthy growth and development in children at risk for obesity.

**Clinical Trial Statement.** Exploratory study to determine effect (immediate and sustainable) of a 3600 min motor skill intervention on motor competence, physical activity, and perceived competence in preschoolers.

1. **Background**

**Developmental Perspective to Promote Sustainable Physical Activity.** Ethnic-minorities and low income populations have an increased risk of cardiovascular disease and engage in less physical activity (PA).^2^ Increasing levels of PA could help prevent cardiovascular disease.^3^ Thus, there is a need to identity effective approaches for promoting positive and sustainable PA in children that contribute to long-term cardiovascular health. Previous interventions aimed at promoting positive and sustainable PA levels in children have had limited success.^1-4^ A gap in prior PA intervention research is that long-term sustainability has not been addressed from a developmental perspective, nor has it addressed the impact of developing motor competence (MC) and perceived motor competence (PMC) on PA.^14,15^ The proposed project is significant in that it will address this gap in intervention research by examining the immediate (pre-post) and long-term (3 yr follow-up) influences of establishing a strong foundation of MC and PMC (≈3600 min dose) on PA levels from early through middle childhood.

It takes time for a young children to develop MC, which does not occur “naturally” or even when provided opportunities to be active,^10,12,16,17^ and PMC (i.e., perception of their movement capabilities).^18^ These processes are cumulative and are modified by growth and maturation processes that are constantly developing during the transition from early to middle childhood.^19^ Thus, the link between the development of MC and PMC is a critical aspect of the Stodden et al.^5^ model (see Figure 1), which hypothesizes that building a strong foundation of MC in early childhood is a critical precursor for PA via the development of accurate PMC.

**Cumulative Role of Motor Competence and Perceived Motor Competence on Physical Activity.** Stodden et al.^5^ developed a heuristic model (Developmental Trajectories Model for Physical Activity) that describes the role that the development of MC has on promoting either positive or negative trajectories of PA. In early childhood (2-5 years), children’s exploratory activity behaviors promote the development of upright posture and locomotor competence that establishes children’s physical independence and the ability to effectively participate in physical activities with other children.^20^ These motor behaviors in early childhood are associated with inflated PMC,^11,21,22^ where effort is equated to success and continued participation in PA is promoted with positive reinforcement. In essence, the development of motor skills and inflated PMC positively promotes effort and persistence in PA in early childhood. The transition from early childhood to middle childhood (6-9 years) is a critical timeframe in the model where it is hypothesized that a divergence in PA levels begin to emerge based on continued development of children’s MC and more accurately aligned PMC levels.

In middle childhood, children possess the cognitive capability to compare their movement behaviors and outcomes to their peers.^5^ The accurate realization of one’s MC (or lack thereof) develops into a more salient motivational factor for their participation in a variety of physical activities. Longitudinal evidence demonstrates that if children do not develop an adequate level of MC by middle childhood, they are at risk for long-term physical inactivity, which is an important factor that contributes to an increase risk of cardiovascular disease.^8, 23,24, 25^ Thus, during middle and late childhood, demonstration of higher levels of MC and continued high levels of PMC are associated with higher PA levels.^6,26-29^ Alternatively, children who have lower MC in middle and late childhood will more accurately perceive their low competence and opt out of PA because (a) they understand they are not as competent as peers and become less motivated to participate in physical activities,^10,11, 21, 30^ and (b) they will find activity less enjoyable than their more competent peers who continue to participate in various types of PA.^7,8,25, 31^ Thus, early childhood is an optimal time to promote positive MC and PMC to decrease the risk of developing unhealthy PA habits across middle and late childhood.

In middle childhood, children possess the cognitive capability to compare their movement behaviors and outcomes to their peers.^5^ The accurate realization of one’s MC (or lack thereof) develops into a more salient motivational factor for their participation in a variety of physical activities. Longitudinal evidence demonstrates that if children do not develop an adequate level of MC by middle childhood, they are at risk for long-term physical inactivity, which is an important factor that contributes to an increase risk of cardiovascular disease.^8, 23,24, 25^ Thus, during middle and late childhood, demonstration of higher levels of MC and continued high levels of PMC are associated with higher PA levels.^6,26-29^ Alternatively, children who have lower MC in middle and late childhood will more accurately perceive their low competence and opt out of PA because (a) they understand they are not as competent as peers and become less motivated to participate in physical activities,^10,11, 21, 30^ and (b) they will find activity less enjoyable than their more competent peers who continue to participate in various types of PA.^7,8,25, 31^ Thus, early childhood is an optimal time to promote positive MC and PMC to decrease the risk of developing unhealthy PA habits across middle and late childhood.

**Preliminary Data was deleted due to need and length.**

**Knowledge gaps**

1. **Intervention Efficacy**. There have been some successful PA interventions in school-age children (i.e., SPARK, Hip Hop for Health, CATCH, Healthy Hearts 4 Kids, GEMS). However, these studies did not examine the impact of MC or PMC to ascertain whether these factors played a role in PA.^14,15^ There is a need to understand the potential long-term impact that MC has on PA, however, there have only been two large-scale US-based interventions that have attempted to promote MC to influence PA in preschool populations.^14,15^ These studies demonstrated either a lack of change in in MC^14^ or a minimal change (.06 eta^2^) in MC.^15^ Not surprisingly, there was no change in PA in either study. One possible cause for the lack of a substantial change in MC, even with a 3600 minute dose,^14^ was the use of untrained teachers for implementation. In addition, previous MC-focused interventions that have attempted to influence PA have not used an evidence-based intervention strategy and/or an adequate dose (<500 minutes)^33,34^ which may be the most critical factors for promoting MC. Many studies have created interventions with the intent on aligning with general childhood movement guidelines (e.g., promoting gross motor activities), but with little to no theoretically-based framework.^14,15^ These issues reiterate that MC will not occur simply because opportunities (i.e., free play) for skill development are provided.^10,16,32^ In fact, recent data demonstrate that many children exhibit no change or even show decreased MC across childhood.^10,16,35^ Lastly, even though PMC is a consistent correlate of PA, no previous intervention strategies have focused on enhancing PMC in parallel with developing MC. Overall, using an evidence-based intervention that focuses on developmentally appropriate, context specific physical activities that promote MC and PMC with trained specialists will allow us to understand the potentially strong impact of MC and PMC on long-term PA. The intent of this study is to deliver CHAMP with trained, movement specialists. The next step is to examine the feasibility of trained, classroom teachers to implement the curriculum.
2. **Motor Competence Measurement Issues.** Variations and limitations in types of MC assessments, qualitative (i.e., movement patterns or process) and quantitative (i.e., product scores like speed and distance) outcomes have limited the ability to compare results across studies and also significantly impact the predictive utility of MC on PA.^5,6,9^ Associations between quantitative (i.e., outcome) and qualitative (i.e., movement patterns) MC suggest different assessments provide unique information (i.e., explained variance) related to motor coordination/control and performance. Thus, measurement of both qualitative and quantitative MC, which has not been attempted in the same study to assess the relationship between MC and PA, will provide a unique picture and clearer understanding of the role MC has on PA.^6^

As children’s activity behaviors are developed in early childhood, our theoretically grounded framework addresses the significance of developing a strong foundation of MC and PMC, which are fundamental to the physical and psychological growth and development of all children.

***CHAMP Theoretical Foundation and Links to Self-Regulation (SR)*.** The Children’s Health Activity Motor Program (CHAMP) intervention is an evidence-based intervention that is grounded in Achievement Goal Theory[^25-27^](#_ENREF_25)^,^[^41^](#_ENREF_41) and uses constructs that support SR in young learners. Specifically, CHAMP is delivered in a mastery climate that emphasizes goal selection and adheres to Epstein’s TARGET structures[^42^](#_ENREF_42) (*T*ask, *A*uthority, *R*ecognition, *G*rouping, *E*valuation, and *T*ime) that motivate learning by promoting choice, autonomy, self-monitoring, and self-evaluation (See Table 1 for how TARGET structures as used in CHAMP relate to SR). **CHAMP engages children in motor skill-building activities and is characterized by the application of effective learning and problem-solving strategies that are grounded in SR constructs.** Specifically, the CHAMP intervention is designed to promote SR by encouraging children to manage their emotions, focus attention, persist, plan and evaluate their actions in order to improve their motor competence and perceived motor competence and enhance their engagement in PA.[^25^](#_ENREF_25)^,^[^26^](#_ENREF_26)^,^[^42^](#_ENREF_42)

**Self-Regulation as a Mechanism of Health Behavior Change in Children.** SR refers to the voluntary control of attentional, emotional, and behavioral impulses in accordance with a long-term goal[^43^](#_ENREF_43)^,^[^44^](#_ENREF_44), and is required in order to sustain concentration and behavioral control while engaging in challenging tasks. SR is comprised of multiple interrelated processes that emerge rapidly across early childhood, including cognitive skills that facilitate working memory, cognitive flexibility, and attention shifting (i.e., executive functions), behavioral skills that allow children to inhibit impulsive behaviors in favor of measured responses (i.e., behavioral inhibition), and emotional regulation skills that enable children to calm down when upset.[^2^](#_ENREF_2) The benefits of SR for long-term social, behavioral, and academic outcomes are well established[^45^](#_ENREF_45)^,^[^46^](#_ENREF_46), but our understanding of the role SR may play in child health outcomes is not well-articulated. SR can operate as a mechanism of health behavior change by shaping the ability to focus on long-term goals (e.g., maintaining a healthy weight), “tune-out” external cues (e.g., advertisements about food), reduce unhealthy behaviors (e.g., sedentary behavior or frequent eating), and engage in positive health behaviors (e.g., PA and exercise) as a means to achieve that goal. Recent evidence from our team suggests has emerged to support the link between SR and health in children, findings that early SR appears to have long-term benefits for children’s weight outcomes[^47^](#_ENREF_47) and associates with concurrent weight.[^13^](#_ENREF_13)^,^[^14^](#_ENREF_14) These findings suggest that targeting childhood SR skills for improvement may help reduce poor health-related outcomes later in life and will likely offer important insight into potential avenues for intervention across a range of health behaviors.


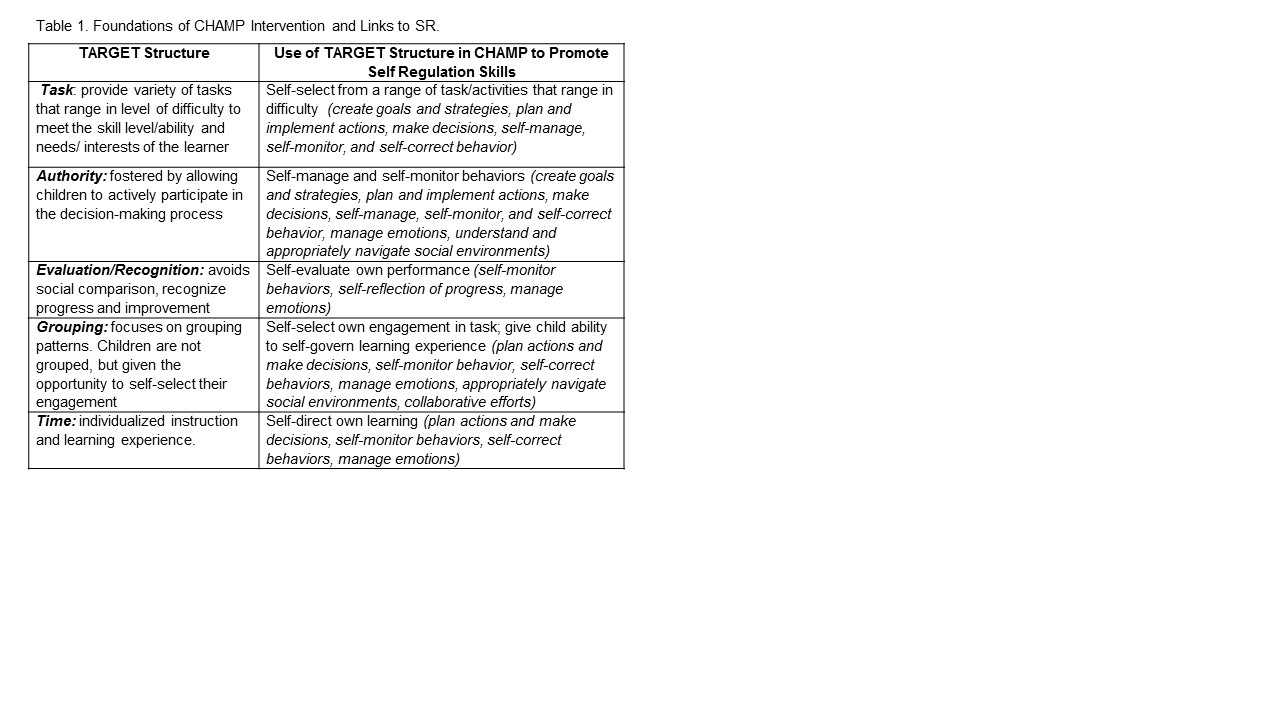


**Innovation**

This project is innovated in multiple ways. First, this study addresses underlying mechanisms of PA based on predictions of the Stodden et al. model^5^, which approaches the problem from a developmental perspective. Specifically, the developmental perspective underscores the importance of understanding that 1) children’s motor development (i.e., MC) is cumulative (not transient like PA) and 2) developing a strong foundation of MC and PMC will influence children’s PA based on their cumulative successes, intrinsic motivation development, and enjoyment in activities. Secondly, the intervention is an intervention strategy that uniquely addresses differences in children’s developmental levels by promoting the development of both MC and PMC on an individualized basis. CHAMP does not equate to a “one size fits all” approach (i.e., high skilled and low skilled children will be in an environment that promotes opportunities to develop that are based on their specific individual needs and choices). The CHAMP intervention promotes a mastery climate that allows each individual child to be successful and learn while promoting intrinsic motivation and autonomy. Thirdly, the measurement of MC is innovative as the concurrent assessment of multiple process- and product-oriented aspects of MC will provide greater predictive utility for assessing the impact of MC on PA. No research has examined MC, as it relates to PA, this comprehensively. Finally, no studies have investigated the long-term effect of developing MC and PMC on PA. As it relates to self-regulation, we will test SR as a mechanism of behavior change in the context of a health behavior intervention (CHAMP) designed to enhance child motor skills and PA. Although interventions have been shown to improve SR, studies have not examined whether improving SR in children has effects on health behavior. No studies have tested SR as a mechanism of behavior change in the context of a health behavior intervention in children. We will examine SR in relation to health behaviors (e.g., low PA) known to associate with obesity risk in young children. Poor SR is proposed to underlie poor health behaviors in adults, yet few studies have examined SR in relation to health behavior during early childhood when SR emerges. Little prior work has examined connections between SR and PA from a movement perspective. The body and brain work harmoniously together to understand and interpret the world around us, and the preschool years represent a period of rapid
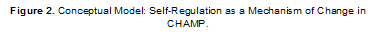
growth and development in cognitive, behavioral, and motor skills. Demonstrating that enhancing SR during this period has positive effects on PA would be a novel, and important contribution.**
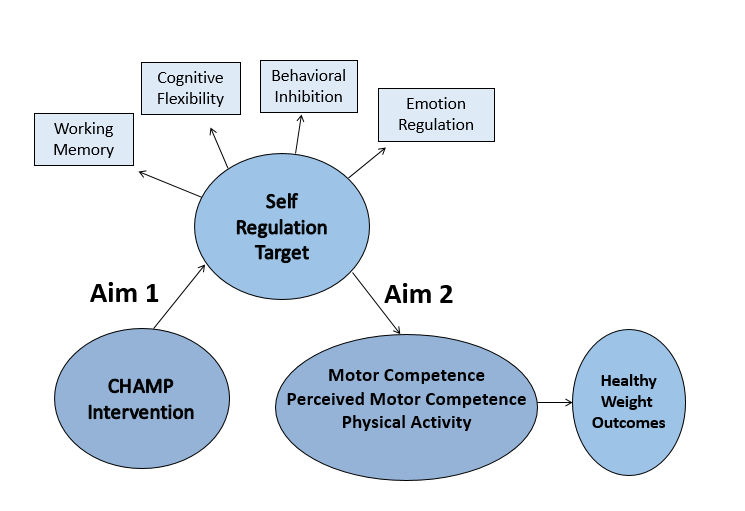
**

1. **Methods**

**Research Team.** **Dr. Robinson is a relatively new faculty member to the University of Michigan and is the PI on the two NIH funded projects.** Dr. Robinson has developed an extensive, evidence-based intervention (CHAMP) that has strong preliminary data. Robinson is the PI on the Parent Award R01 (R01HL132979-01) and a motor developmentalist and pediatric exercise scientist who developed CHAMP and has extensive experience in the implementation of interventions in early childhood and school-based settings in racial/ethnic minority groups. She served as the PI/Co-I on funded projects (federal and private) projects (R03-NICHD, R01-NHLBI, Robert Wood Johnson Foundation). She has also received additional research and hands-on didactic training in developing a sustainable independent research program for career advancement through the NHLBI-PRIDE program. Dr. Robinson has assembled a strong research team (Co-investigators are Drs. David Stodden, Natalie Colabianchi, Lu Wang, and Dale Ulrich). These individuals will provide seniority, experience, and leadership support relative to methodological and logistical issues and suggestions for data analysis and interpretation of findings (see biosketches and letters of support). Co-I Miller (University of Michigan) is a Developmental Psychologist and expert in self-regulation and obesity risk. She is an experienced NIH investigator and has examined self-regulation as a mechanism of behavior change in children in two SOBC-funded projects, one on toddler self-regulation and eating behavior (1R01HD061356) and one a currently-funded intervention study to test the impact of SR interventions on eating behavior in school-aged children (UH2HD087979). Both PI Robinson and Co-I Miller also have extensive experience with the proposed measures, running intervention studies, and working extensively with Head Start populations. PI Robinson and Co-I Miller are collaborators and Co-PIs on a Pilot and Feasibility grant funded by the University of Michigan Momentum Center for Childhood Obesity entitled *Self-Regulation and Health Outcomes in Young Children*. Collectively, Robinson has assembled a team that has unique strengths and expertise to carry out the proposed work. The entire team has demonstrated experience in implementing federally-funded projects (NIH R01s, R21, R15, R03; Robert Wood Johnson Foundation; and Department of Education) which have included large-scale projects in schools, implementing evidence-based interventions, and measuring MC and PMC and PA. Consultant Dr. Kip Webster (Augusta University) has expertise in the TGMD and motor skill analysis and will assist with coding the video-recorded TGMD motor skill assessments. Dr. Cathy Antonakos (University of Michigan) is a research area specialist for the UM School of Kinesiology and will be assisting with physical activity data analysis.

Additional staff and support for the project includes: Kara K. Palmer, MEd, and Katherine Andrews are PhD students in the School of Kinesiology, and Katherine Chinn, is a Research Technician. Bryan Terlizzi is a PhD student in the University of South Carolina Department of Physical Education and will be assisting with data collection for the project. Bryan has a Master’s of Science in Health, Exercise, and Sport Science from The Citadel (2017). He has served as part of a research team on an NIH funded study analyzing qualitative and quantitative movement data in underserved children, as well as several other short-term practical experiences with young children in the classroom setting. Matthew Stewart is currently a Kinesiology Ph.D. student whose research is focused on environment and policy. Matthew earned his Masters from Iowa State University where he worked in a lab that focused on accelerometry. He has specialized experience with ActiLife and Actigraph data analysis. Indica Sur and Marcia Wallin provide administrative support. These individuals will assist with various aspects on this project that includes recruitment, consenting, data collection and data processing/entry. Additionally, Andrews and Palmer have training in motor skill development in pediatric populations. Palmer and Andrews will implement the CHAMP intervention.

**Theoretical Foundation and Intervention Approach.** **The Children’s Health Activity and Motor Program** **(CHAMP)** is grounded in Achievement Goal Theory ^37-38^ and adheres to the TARGET structures – *t*ask, *a*uthority, *r*ecognition, *g*rouping, *e*valuation, and *t*ime; ^40, 41; Table 1^. Achievement Goal Theory refers to our beliefs, attributions, and affect that contribute to our behaviors and represents the way individuals approach, engage, and respond to educational and classroom-based activities ^37-38^. This evidence-based intervention that the PI developed as part of her dissertation in 2007. Data supports that the theoretically-grounded approach enhances both motivation and movement behaviors of the child and promotes MC, PMC, PA, and movement knowledge (see PI Robinson work^10, 12-13, 16, 42-43^ and Preliminary Data section C.3). CHAMP focuses on the process of learning and skill mastery.^37,38^ CHAMP provides children the opportunity to navigate the movement environment they deem appropriate for their own level of development.^10,16,38,38^ For example, CHAMP provides a range of movement activities with varying levels of difficulties to accommodate a range of developmental levels (see provided CHAMP example). Additionally, CHAMP focuses on the process of learning motor skills rather than the product (outcome) of learning motor skills, which aligns with early childhood principles of development and learning^19^ and with the best practices of motor development.^39^ The focus on developmental processes targets children’s intrinsic motivation and persistence reinforces learning; thus, this intervention promotes children’s PMC and continued development of MC and PMC, beyond the specific intervention process.^37-38^ Three theoretical tenets are crucial to CHAMP^37,38,40,41^:

1. **The relationship between effort and personal progress.** CHAMP promotes personal effort, progress, and improvement of skills. Effort and progress help young children build patterns of achievement behavior that have positive long-term implications for learning and persistence in activities across the lifespan.
2. **Learners’ self-selection of tasks.** Learners select tasks that effectively challenge their ability and enhance their competence, which leads to confidence in their capability to succeed on other skills.
3. **The environmental climate.** Learners are intrinsically driven to be engaged in environments that give them the authority and the opportunity to be a decision-maker. These tenets are implemented with CHAMP through six TARGET (*t*ask, *a*uthority, *r*ecognition, *g*rouping, *e*valuation, and *t*ime) structures established by Epstein.^40,41^ Table 1 provides an overview of the TARGET structures.

For the motor skill intervention, preschoolers will participate in CHAMP for (4, 30-minute classes a week or 3, 40-minute classes a week, based on the needs of the school schedule)~~.~~ **~~30 minutes/day 4 days per week for 30 weeks (≈3600 min dose)~~** under the direction of a trained instructor and assistant. CHAMP creates a learning environment were children have the opportunity to establish behaviors that reinforces decision-making regarding their participation in a variety of challenging and diverse movement and physical activity tasks. Each 30-min session consists of three parts:

(a) 2 -3 min of motor skill introductory activity that includes a group motor activity, the teaching of the lesson that includes a demonstration and understanding of developmentally appropriate learning clues;

(b) 25 min of motor skill instruction and practice (i.e., ‘active motor engagement’), participants will be encouraged to move through 3-4 motor activity stations that align with the TARGET structure (Table 1 for TARGET structure); and

(c) 2 -3 min motor skill closure activity that involves a review of the lesson and critical elements.

The climate provides participants with the opportunity: a) to engage in a variety of activities, b) to independently choose activities, c) to use established rules, d) to share in the decision making process, e) to work with a variety of their peers, and f) to participate in personal evaluation of progress. The *Task* aspect of TARGET (Task, Authority, Recognition, Grouping, Evaluation, and Time, Table 1) involves the content and sequencing of the motor curriculum, as well as the difficulty of the task with a variety of developmentally appropriate motor tasks. For each day of instruction, the instructor provides 3 – 4 active motor stations and 3 – 4 levels of task difficulty for each active-motor activity. However, if a learner chooses to independently modify or extend a task to meet their needs, it is appropriate due to the high autonomy nature of CHAMP. Students are challenged with tasks that reflect the array of learners’ abilities. This enables students to move up and/or down task continuum/ progressions to meet their interests and developmental needs. Regarding the *Authority* dimension, CHAMP participants independently choose activities and actively participate in the process of establishing rules and decision-making. Children chose where and when they engage, how long they engage, and with whom they engage. In relation to the *Recognition* structure, emphasis is placed on student progress, effort, and improvement and occurs through both intrinsic and teacher feedback. The instructor will provide recognition and feedback to each student based on their individual progress and evaluates children in reference to their continued skill progression and individual rate of improvement. Intrinsic visual feedback will occur independently at each active motor stations (e.g., when throwing at a target a successful throw will result in intrinsic motivation from hitting the target). For *Grouping,* children have the option and freedom to work in heterogeneous small groups, with peers, or individually. Children can change their grouping structure as often as they like. An *Evaluation* will be provided to each participant by the teacher that will focus on each participant’s accomplishments, efforts, and performance. Finally, Time pertains to the amount of self-paced time allocated in the active motor engagement session (i.e., 25-min). Children have the authority to decide how much time they spend practicing in each station; thus, the amount of time spent on each skill varies for each child. In CHAMP, children are given opportunities to make choices on specific task engagement, participate in a variety of learning experiences and peer interactions (e.g., cooperative and independent), involve themselves in leadership roles, and are allowed to choose the length of practice time necessary to develop a specific aspect of a skill (i.e., based on personal capabilities).

The CHAMP session is designed to take place in a school gymnasium or large indoor open space (i.e., multipurpose room) that has been designated at each preschool center. The space needed will accommodate 25 – 28 preschool-children and two classroom teachers (i.e., 1 instructor and 1 assistant). For this study, the instructors will be graduate trained movement science specialists. Equipment for the implementation of CHAMP consists of items typically found in preschool movement or early childhood physical education programs or can be obtained for minimal costs (e.g., balls of various sizes, bean bags, cones of various height, striking objects, targets, etc).

**Methodology**

Research Design. The proposed study is a two arm cluster randomized control trial with follow-up. Classes of children will be randomly assigned to treatment (i.e., movement intervention – CHAMP or the normal movement program provide by their educational center (Control; outdoor free-play activity in a large playground area with a variety of play structures that promote movement and physical activity but no planned instruction nor activities).

Setting and Participants (Recruitment). This study will take place in three Head Start centers (i.e., Perry, Ford, and Beatty) located in Ypsilanti Michigan (Midwest). The racial make-up of the centers are 65.9% Black or African American, 19.14% White (non-Hispanic), 8.82% Hispanic/Latino, 3.26% Asian-Pacific Islander, 2.82 other races (two or more), and 0.06% Native American. Participants will be 300 preschool-age children age 3.5 years and older that encompass diverse settings (30 classes; 15 CHAMP Treatment and 15 Control; 150 preschoolers per condition). Thus, participants will primarily be from urban settings and include low and middle SES black/African American. The three Head Start centers are on the same campus for the three elementary schools (Grades K – 5) where the students will enroll for their formative schooling (i.e., Erickson, Adams Stem, and Holmes Elementary School), which will aid with retention over 3-years of follow-up.

Inclusion/Exclusion Criteria. Preschoolers who are ≥ 3.5 years are eligible to participate. Any preschooler with a specified developmental and/or physical disability (noted from school records) will receive the intervention if part of a CHAMP class, but data will not be used for research purposes.

PLEASE NOTE - CHANGE TO THE RECRUITING N: MODIFICATION WAS MADE TO THE TARGETED N FOR RECRUITMENT EACH YEAR. SPECIFICALLY, 150 PARTICIPANTS (75 TREATMENT AND 75 CONTROL) WILL BE RECRUITED IN YEAR 1 AND 150 WILL BE RECRUITED IN YEAR 2. THE OVERALL, N FOR THE PROJECT REMAINS AT 300. THIS CHANGE WAS APPROVED BY THE FUNDING AGENCY.

Informed Consent. Study enrollment will begin after receiving human subjects approval from the Institutional Review Board for Research Involving Human Subjects at the PI’s University (University of Michigan) and obtaining parental consent/child assent (Fall of year 1). All children enrolled the preschools who meet the inclusion/exclusion criteria will have the opportunity to participate in this study. We are confident in our ability to obtain and maintain our targeted N. Avenues that we will take to achieve this include this following:

1. the PI and members of the investigative team presenting at “Welcome Night” to provide an overview of the project and run a family movement activity.
2. be present for pick-ups and drop-off to address any questions from parents and guardians about the project.
3. discuss ways with the teachers, staff, and administrators within the schools to keep open communication with parents/guardian.

Upon receiving IRB approval, the consenting process will occur through multiple methods (see below Informed Consenting Methods for specific details). Upon returning the IRB form and to compensate the parent/guardian for their time, parent/guardian will receive a one-time $5.00 cash incentive. Additionally, fully completed Family Questionnaires will be entered in a drawing to win one (1) of eight (8) $50.00 Gift Cards to Wal-Mart or Kroger Shopping Center. A random drawing will be used to select four (4) completed surveys from each preschool. The child ID number for each completed survey will be placed in a Choice Picker, random selection software, and selected. Completed Family Questionnaires will be entered in a drawing to win one (1) of eight (8) $50.00 gift cards to Wal-Mart or Kroger Shopping Center (i.e., 4 for each school). Two (2), $50 gift card drawings will occur each day (i.e., one (1) for each preschool) and will occur over four (4) consecutive days. ID numbers of completed surveys will enter four discrete pools on each day of the drawing (e.g. Subjects 1-37 on Day One for each site, Subjects 38 - 75 on Day Two for each site,), and Subjects 76-113 on Day Three for each site, Subjects 114-150 on Day 4 for each site). Parents will receive a yearly report regarding their child’s assessment findings. Early withdrawal from this study will forfeit future compensation. The decision to use Wal-Mart and Kroger gift card over a Visa/Mastercard gift card was due to cost, location, and variety. These stores do not have a surcharge of $4.95 – $6.95 to load funds, there are several locations in the area, and they provide a variety of options for purchase.

Informed Consenting Methods

1. **Preschool Registration/Sign Up Day.**The PI and members of the investigative team will be present at the Registration/Sign-Up Day to address any questions from the parent(s) and to discuss the project. Informed consents and Family Questionnaire packets will be available to parents.

The four (4) other steps will be completed to achieve our targeted N.

a.      **Home visits.**The Family Support Staff Personnel will contact families and request for a research staff that has completed the PEERRS and GCP training (PI-Robinson, Research Staff – Palmer and/or Chinn) to attend the required Home Visit with the Classroom Teacher or Center Family Support Staff  Personnel beginning Sept 5^th^. The Research Staff will explain the study 1:1 with the parent/guardian, go over the inform consent letter, answer any questions, and assist the parent/guardian with the Family Questionnaire.

b.      **Welcome Night.** The PI and member of the investigative team will present an overview of the program, go over the assessment, and IRB Consent Form with the parents on the Welcome Night (2 days before the 1^st^ day of School). The team with also have some fun games and movement activities set up for Family Fun Night.

c.      **Mass Distribution.**Each parent/guardian will receive a packet that contain the informed consent letter and the Family Questionnaire. Letters will be distributed by classroom teachers in a sealed enveloped  on the first day of school and returned in a sealed envelope that is provided.

d.      **Drop-off and Pick-up times.** The PI and/or members of the investigative team will be present give an overview of the study and go over the IRB Consent Form  during drop-off and pick-up times in the morning and afternoon at the center.

Parents/guardians who do not consent to participant in the project after the mass distribution and/or Welcome Night methods will be contacted through three more consenting methods. Then no more attempts will be made.

Parents who consent to participate in the project will receive a copy of the consent form for their personal records. Parents will receive a copy of their child’s results an overview of the study’s findings and how their child’s participation contributed to advancing research that focuses on health-related concerns in pediatric populations. A parent and/or child has the right to withdraw from the study at any time. Participation is voluntary and will in no way influence their relationship with the preschool centers, School of Kinesiology, nor the University of Michigan. All children who have received parental consent to participate in the study must also provide verbal assent. The script for the verbal assent is:

***For Child Assent, the script is verbally delivered to the preschoolers and then they are asked to assent by saying Yes/No and showing a Happy/Sad face.***

*“Good Morning (Student Name). Your Mommy/Daddy told me it is okay for you to be in this project. We will play together during school in some games. I will also see how big you are, how well you move, and show you some pictures/videos and ask you to tell me “which one moves most like you.” We will see how much you play by wearing this small device on your wrist (show the child the device). We will also play some games with each~~either~~ other and on a computer screen.*

*Do you want to play with me? Yes or No.*

*Point to the smiling face to indicate* ***YES*** *and the frowning ace to indicate* ***NO****.*

No, parental reconsent, only child reassent. Only, will re-consent with parents if changes are made to the study.

Children are reassessed during grades K, 1, and 2 following the intervention to examine the long-term effects of the CHAMP program. Re-consenting process will not occur with parents but they will receive reminders and a letter about upcoming assessments for the PATH study. Parents will also receive their child’s assessment reports.

We will re-assent children to participant in follow-up assessment. Since this is a 2 year cohort study. We will be in the site on a regular basis and quite familiar with the families and children. Re-consent with parents will occur there are changes made to the study.

Assessment reminders will be distributed to parents as a reminder for the follow-up assessments. This reminders will be given to the students in their weekly folder materials that go home to parents. Also, each center has a call system that center a message to the parents registered phone number. The Principal or the School Administrative Assistant stated that will send a reminder on the call system leading up to follow-up assessments for the PATH study, state that there is a reminder in their child folder for those who are participating in the project, and to contact the CMAH Lab.

**Subject Participation**

Upon receiving IRB approval, recruitment will begin. Informed consent will be obtained from parent. After consent has been obtained, each preschoolers will be assigned an identification number. This number will be used in the data collection and to ensure that the data remain de-identifiable (see data storage and confidentiality section). Participants will be then assigned to a treatment groups (CHAMP or Control).

Once consent has been provided data collection will commence. Demographical measures include: child race/ethnicity, child sex, child age/birthdate will be obtained from parents during the consenting process.

All assessments used in this study are well-validated and tested instruments in preschool populations (noted below). Once pre-test measures have been completed the treatment will be implemented that will be following by post-test measures. From previous experience, we estimate that pre-and post-test measures will be measured over multiple school days (i.e., 5 days) across a 2 – 3 week period. Each session will take about 20 – 25 minutes to complete and we will work with the teachers and center director to ensure that the testing sessions align with the centers’ existing schedule. We will follow the children and complete the measure at the start and end of Kindergarten, during 1^st^ and 2^nd^ grade as part of the follow-up assessments.

**The Children’s Health Activity Motor Program (CHAMP) Implementation**. CHAMP will be implemented during the scheduled movement sessions at each participating preschool (see preschool letters of support, Appendix A). CHAMP consists of (4, 30-minute classes a week or 3, 40-minute classes a week, based on the needs of the school schedule)~~. ≈ 30 weeks per year that will equate to ≈3600 minutes of intervention time (30 min sessions X 4 days per week X 30 weeks per year).~~ Each session will consist of three parts: (a) 2 -3 min of motor skill introductory activity, (b) 25 min of motor skill instruction and practice, and (c) 2 -3 min motor skill closure activity. The control condition will be the preschools typical activity programs (i.e., outdoor/freeplay recess) and will be implemented according to the existing procedures within the preschool center. The centers outdoor program consist of outdoor free-play activity on a large playground area with a variety of play structures (swings, slides, ladders) that promote gross movement and activity in preschoolers. For the control condition, there will be no planned instruction nor activities provided by the classroom teachers. CHAMP will be implemented by two trained movement science specialists (1 instructor and 1 assistant). Instructors will attend a 3-day (4 hr/day) workshop/training session prior to the start of the intervention. The training workshops will be conducted by Dr. Robinson, an expert in movement/physical activity programming in early childhood settings and the developer of CHAMP. During the training workshops, instructors will learn the theoretical approach of CHAMP, see and actively participate in a CHAMP session and demonstrate effective implementation of the CHAMP intervention. During the training session, instructors must be able to provide a range of cues and prompts to promote skill learning, set up and demonstrate basic task progressions for motor skills, modify tasks based upon the child’s present level of performance and rates of improvement, and implement CHAMP with fidelity, appropriate transitions and pacing, while ensuring safety. All training sessions will be digitally recorded and available through a private YouTube channel. A printed manual will also be provided to promote fidelity of the intervention. During the intervention, instructors will wear wireless microphones and digitally record each CHAMP session in order to determine fidelity. Ensuring fidelity demonstrates a measure for the internal validity of CHAMP.^44^ Fidelity will be calculated from the digital recordings using a systematic observation tool established for mastery interventions.^45^ CHAMP fidelity checks will be conducted during week 1, 3, 7, 15, and 25. Dr. Robinson will deliver fidelity support (additional training) to instructors by providing adjustments to teacher behaviors if CHAMP specialists are not implementing CHAMP with fidelity. Concurrently, during weeks 1, 3, 7, 15 and 25, systematic observations tools at the control site will also occur for additional internal validity.^46^

**Data Collection.** Trained graduate students external to the project (i.e., not involved in intervention delivery) will collect data using standardized instruments. In August (first month of the academic year), informed consent will be obtained and will be followed by the collection of pretest measures. Based on past experience with large sample data collections ^10,11,12,13,16,47,48^, we estimate that four, 30 – 35 minute sessions are needed to complete the assessments (e.g., Height, Weight, MC, and PMC, Self -Regulation) for each class and a fifth day will be used for any absences (i.e., make-ups). Physical activity will be measured using accelerometry (ActiGraph) for 7 days to capture the children’s engagement during the weekday and weekend. A minimum of 3 weekday and 1 weekend day will be required for data analyses. Accelerometers will be calibrated with individualized measures (i.e., height, weight, sex, and age) collected at pretest. Data collection for both testing periods in year 1 (see project timeline, Table 2) will take a maximum of 6 weeks, which will allow for the 30 weeks of intervention to be implemented. Following initial data collection, physical activity participation and CHAMP will commence. Follow-up assessments will occur at the conclusion of the CHAMP intervention (post-test) end of Year 1 to examine the immediate effect of CHAMP on MC, PMC, and PA. Additional follow-up assessments will be completed to examine the sustainability effect of CHAMP during Year 2 – 4 (Spring of Year 2, 3, and 4).

Data collection in the elementary schools following the preschool intervention year will be facilitated in the same manner as preschool sites. The preschool centers are affiliated with specific elementary schools and are on the campus of the elementary schools. Permission for the project has been provided by the Superintendent, Center Directors, and Principals (see letters of support) of the elementary schools as well. Based on previous longitudinal work^32^ expected attrition due to relocation issues and/or disinterest in continuing the study will be 30% over 3 years. Thus, our initial oversampling will be 30%. To promote children’s participation over the course of the study, we will provide $10 gift cards to each child for each successful data collection and accelerometer return in year 1 (2 times) and during the follow-up assessments. Additional incentives will also be provided to children for their participation (e.g., pencils, stickers, wristbands, etc.). To further maintain the necessary sample size, we will contact children (via parental contact information) that have relocated within the immediate area and conduct a home visit or University visit to continue testing. The same participant incentives will be provided for relocated children who continue testing.

**Variables and data sources.** This section describes the variables of interest and their data source. All data will be collected by trained individuals that are blind to the purpose of the study. The preschool personnel/staff have no role in data collection.

1. **Demographics. Date of birth, sex, and race/ethnicity** will be collected as supporting demographical information and collected through self-report through the IRB consenting process by the parent/guardian. Upon return and to compensate the parent/guardian for their time, parent/guardian will receive a one-time $5.00 cash incentive.

Time Frame: Measured at baseline (Month 0).

1. **Body Composition.** Body composition will be calculated using standing height will be recorded on a SECA 769 to the nearest 0.1 cm. Weight (mass) will be rounded to the nearest 0.1 kg using a digital scale. Researchers will assist preschoolers in removing their shoes and socks. Students would stand still on the appropriate scale to complete the assessment. Waist circumference will be assessed with a non-elastic plastic tape measure. The tape measure will be placed around your child’s waist in a standing position. Two measures will be taken and the average of the two will be used for data analyses. Preschoolers will complete the body composition assessments in small groups of 4 – 6 and should take 12 – 15 minutes to remove/put on shoes/socks and take the assessment for each group. The assessment will be completed in a designated classroom/area at the child care center. This measure will be collected first as height and weight information along with birthdate and sex are needed for the calibration of the physical activity monitoring devices (Previously approved in PROTOCOL HUM00108917, HUM0011789).

Time Frame: Measured at baseline (Month 0), post-intervention (month 9), and follow-up assessments at the end of years 2, 3, and 4.

1. **Motor Competence (MC).** All MC measures will be evaluated in an indoor space (i.e., gymnasium or designated indoor activity space) during the time allocated for movement and physical activity. As recommended to increase the predictive validity of MC for physical activity, we will assess MC using multiple measures of MC that include both process and product measures.^6^ All measures will be assessed concurrently.

Time Frame: Measured at baseline (Month 0), post-intervention (month 9), and follow-up assessments at the end of years 2, 3, and 4.

1. **Process measure.** The Test of Gross Motor Development (TGMD)^50^ will be used to assess motor skill development and learning. The TGMD is a measure of fundamental motor skill competence in children ages 3 to 10 years and consists of locomotor (run, jump, gallop, slide, hop, leap, and skip) and object control skills (throw, catch, dribble, underhand throw, kick, one-handed forearm strike, roll, and two-handed strike off a tee). Scores for the two subscales (locomotor and object-control) of the TGMD will be reported as raw scores for each skill. Mean test-retest reliability coefficients were reported as .88 for locomotor items and .93 for object-control items.^50^ Interrater reliability coefficients for both subscales were reported as .98.^50^ The TGMD will be videotaped with digital video cameras by trained researchers. All TGMD videos will be coded by in the CMAH Lab under the direction of PI Robinson. Specifically, PI Robinson and an approved research staff member will complete the video analyses. Inter-rater reliability will be established by another approved research staff member and Co-I Dale Ulrich, the developer of the TGMD will be available to consult if there are any issues on concerns. An overall score for total MC of 0 – 100 will be used for data analyses. (Previously approved in PROTOCOL HUM00108917, HUM0011789).
2. **Product measures.** Product scores (kick and throw velocity, catching %; hop and jump distance and running speed) are developmentally valid and sensitive discriminators of MC^47,48,50-59^ and have been used in previous studies by the Co-I (Stodden).^47,48,51,57,58,59^ Ball velocities will be measured with a radar gun.^47,51,57^ A tennis ball 5cm and a playground ball (18cm diameter) will be thrown and kicked, respectively, with maximum effort from 20 feet for 5 trials.^51^ Jump distance (cm) will be measured for the standing long jump.^51^ The average speed (throwing, kicking) and distance (jumping) of the best 3 out of 5 trials will be used for data collection.^51^ Children will attempt to catch a tennis ball 5 times with a research staff tossing the ball with a standardized procedure according to age.^50^ The number of catches out of 5 will be used. The average hop stride length (from heel to heel) for 4 hop cycles of each leg will be calculated by digitizing stride length using motion capture (Dartfish, Inc.). Running speed will be calculated from four consecutive stride cycles using motion capture (Dartfish, Inc.). Hopping, running and jumping data will be normalized to a percent of standing height.^51.^ Product scores will be analyzed from videotape through video motion capture processing with Dartfish software. (Previously approved in PROTOCOL HUM0011789).
3. **Perceived Motor Competence (PMC).** Perceived motor competence will be assessed with the **Harter and Pike Pictorial Scale of PMC and Social Acceptance (PSPCSA**)^60^ and the **Perceived Fundamental Motor Skill Competence Scale.**^61^

Time Frame: Measured at baseline (Month 0), post-intervention (month 9), and follow-up assessments at the end of years 2, 3, and 4.

1. The **PSPCSA** will be used to measure the child’s global perceived physical competence. The PSPCSA physical competence subscale consists of six items that are presented in pictures and each child will select a picture that is more like them. The six items included are swinging, climbing, tying shoe laces, skipping, running, and hopping.^62,63^ Reliability for internal consistency of the individual items ranged from .65 to .89, with a reliability of .86 for the combined subscale measure. The reliability of the total scale is .89.^60^
2. The **Perceived Fundamental Motor Skill Competence Scale (PFMSCS)** is a video-based assessment that allows the child to see the entire motor skill in action rather than a static picture.^61^ The scale is the identical layout and item structure to the PSPCSA but align with the 12 fundamental motor skills of the TGMD.^50,64^ Reliability for internal consistency ranged from .69 to .84, with a reliability of .84 for the combined subscale measure.^61^

For both assessments, children will (1) select the picture/video that is most like him or herself. One picture/video depicts a child who is competent and the other shows a child who is not skilled; (2) focus on the designated pictures/videos and indicate whether he or she is just a “little bit” or “a lot” like that child. Separate pictures/videos for girls and boys will be used in accordance with the manual procedures. The range of scores for each item on the subscale is 1 (low competence) to 4 (high competence). Both assessments are established tools within the preschool population and standardized test protocols will be used.^60,61^ Additionally, PI-Robinson has a vast amount of experience with the implementation of the tool in preschool populations.^10-11,^ (Previously approved in PROTOCOL HUM00108917, HUM0011789). Also the Perceived Fundamental Motor Skill Competence Scale (PFMSCS is a digital video/media clip in order to be viewed review need to be presentation view of a powerpoint program).

**Physical Activity.** Children will be asked to wear an ActiGraph accelerometer (Pensacola, FL). This tri-axial accelerometer is small (3.5cm x 3.5cm x 1.0cm), lightweight (14g), and can be comfortably worn on an elasticized belt under or over outer garments. Accelerometry is a valid and reliable measure of the frequency, intensity and duration of PA among children at school and in free-living settings. Participants will be instructed to wear an accelerometer and a uniform location (i.e., non-dominant wrist) and attached by an elastic band. While the ActiGraph is water-resistant and able to be worn during bathing and swimming, participants will be given the option to remove the belt and device while engaged in water activities as some people report that wearing a wet belt can be uncomfortable. Participants will be asked to wear the same accelerometer for 7 full days (5 week and 2 weekend days). Data will be collected at 80 hz. Cut points from a study in preschoolers by Butte et al will be applied to activity counts.^65^ This study utilized information from all three axes (versus just the vertical axis) thus time spent in sedentary, light, moderate, and vigorous categories will be defined as vector magnitudes of up to 819 (sedentary), 3907 (light), 6111 (moderate) and above 6112 for vigorous activity. Collecting data at 80 hz will allow for use of emerging methods, such as pattern recognition or cut points based on newer validation studies, available at the time of study commencement. The primary outcome will be minutes of moderate-to-vigorous PA (MVPA) per day although additional analyses will examine minutes of light/moderate/vigorous activity per day since PA recommendations do not require that all activity be of a moderate or vigorous intensity. In order to be considered valid wear, participants must obtain at least 9 hours of valid accelerometry data per day for at least 4 days (3 week day and one weekend day).^66^ Non-wear time will be defined based on the work of Choi (i.e., 90 minute window of consecutive zeros with allowance of up to 2 minutes of non-zero counts).^67^ While conflicting studies exist that suggest shorter time intervals may be appropriate for estimating non-wear time in young populations,^68^ the choice of the non-wear time interval has a much greater effect on estimates of sedentary behavior compared to estimates of MVPA (the primary outcome). Prior to the onset of each PA data collection, a research staff member will explain the purpose of the accelerometer and demonstrate its use and placement. Accelerometers will be attached using a neoprene elastic belt and worn by the child for a 7-day period both at home and school. Compliance with wearing accelerometers will be facilitated by techniques described by Trost: 1) a letter to the parents explaining placement and providing a simple diagram, 2) use of parent-teacher conference nights to physically show the parent how to place the accelerometer, 3) smiley face on accelerometer 4) training in the classroom to get the children use to wearing the devices prior to formal data collection, 5) periodic phone calls and/or text messages will occur 2 days after the devices has been distributed to prompt, 6) checking device placement of children in school first thing in the morning (call home and/or sending a note if the device is not worn), and 7) a sticker reinforcement chart to promote daily wearing of the device to school, and 8) an incentive will be provided to each participant upon the return of the device, the incentive is a $10.00 gift card or $10.00 worth of incentives (i.e., gifts/prizes). (Previously approved in PROTOCOL HUM00108917, HUM0011789).

Time Frame: Measured at baseline (Month 0), post-intervention (month 9), and follow-up assessments at the end of years 2, 3, and 4.

Standardized behavioral and computer-based tasks that have been used in prior research and normed in children this age will be used to assess cognitive flexibility, working memory, and behavioral inhibition. Child performance on each task generates a score. Both investigators have used these tasks and approaches in prior research. Each participant will receive an incentive at baseline and post intervention for completing these task, the incentive is a $10.00 gift card or $10.00 worth of incentives (i.e., gifts/prizes).

1. **The Dimensional Change Card Sort (DCCS) Task**. This is an iPad based assessment and will measure of cognitive flexibility, requiring children to learn and remember a rule and apply it one way, and then apply a new rule as instructions change. Children match pictures that vary by two dimensions, shape (rabbit vs. boat) and color (brown vs. white). Children complete trials with one set of instructions, then another, and are encouraged to go as fast as they can without making mistakes. 0 = did not pass color sort (Less than 5/6 items correct on color sort), 1 = pass of color sort, fail on shape sort (At least 5/6 items correct on color sort but less than 5/6 items correct on shape sort), 2 = pass shape sort, fail advance trials (At least 5/6 items correct on both color and shape sort but less than 9/12 items correct on advance/border sort), 3 = pass advance trails (At least 5/6 items correct on both color and shape sort and at least 9/12 items correct on advance/border sort.)

Time Frame: Measured at baseline (Month 0), post-intervention (month 9).

Cohort 2 participants will not do this measure..

1. **Working Memory. Working Memory (i.e., Mr. Ant)** is an iPad based assessment and will be assessed using a visual-spatial working memory task, which requires children to accurately recall information they have seen before; this type of task is appropriate for children this age. Specifically, children are shown visual information (stickers on the body of a cartoon character) and next shown the character without the stickers, and asked identify these location after a brief retention interval. Test trials increase in difficulty (i.e., working memory demand) as the task progresses. Test continues until a max of 8 levels or failure on all 3 trials at the same level of difficulty. For working memory, faster more accurate responses reflect better self-regulation. Working memory capacity was indexed by a point score: one point for each consecutive level in which at least 2 of the 3 trials were performed accurately, plue 1/3 of a point for all corrected trials thereafter.

Time Frame: Measured at baseline (Month 0), post-intervention (month 9).

Cohort 2 participants will not do this measure.

1. **Head-Toes-Knees-Shoulder Task (HTKS Task; Behavioral Inhibition).** The HTKS Task is a direct observational assessment of behavioral regulation and takes 10 minutes to complete (Ponitz et al., 200, Cameron et al., 2007). It reflects a child's ability to remember commands and to behaviorally inhibit a pre-potent response in favor of a less-dominant response. Children are asked to play a game in which they were instructed to do the opposite of what the experimenter states. For example, the experimenter instruct them to touch their head (or their toes), and instead of following the command, children will be directed to do the opposite and touch their toes (or their head). Children are given four practice tests and the instructions are repeated up to three times during the practice tests. After the practice tests are administered, the testing portion of the task are given. There are a total of 10 items in random order, with possible item scores of 0, 1, or 2 for each item. Higher scores indicated higher levels of behavioral regulation. A 0 is incorrect, 1 is a self-correct (defined as any motion toward the incorrect response but where the child then stopped and responded correctly), and 2 points are given if a child gave the correct response without hesitation or a prior movement to the incorrect response. Scores across trials are summed to reflect self-regulation. The task has begins with 6 practice items and between the first and second set of items there are 5 more practice trials. The score range is 0-40; higher score equal better behavioral inhibition. (Procedural manual)

Time Frame: Measured at baseline (Month 0), post-intervention (month 9), is attached)

(Previously approved in PROTOCOL HUM00108917).

Cohort 2 participants will not do this measure.

1. **Observed SR.** We will also evaluate child ability to stay on-task during HTKS as an aspect of SR. Incorrect responses and false starts/self-corrections are recorded in the HTKS task, with fewer such responses indicating greater SR. Child compliance and engagement will also be rated using the Child Assessor Report which has been used in prior SR trials. Co-I Miller has decades of experience coding such behaviors from video analyses and will train independent observers to code these behaviors (criterion of Cohen’s Kappa>.70 or ICC>.80). Observed self-regulation will also evaluate the child ability to stay on-task during HTKS as an aspect of self-regulation. Child compliance and engagement will also be rated using the Child Assessor Report (i.e., Child Behavior Observation) which has been used in prior SR trials. This 10-item questionnaire is scored on a 0-3 scale. The mean is taken and higher score indicates better observed self-regulation behaviors and coded my trained research. Co-I Miller will code and analyze the HTKS and Child Assessor Report (i.e., Child Behavior Observation) data. (Previously approved in PROTOCOL from Co-I Miller).

Time Frame: Measured at baseline (Month 0), post-intervention (month 9).

Cohort 2 participants will not do this measure.

1. **Teacher-Reported.** Teachers will report on child emotion regulation, another key aspect of SR at this age, using the 24-item Emotion Regulation Checklist (ERC), which generates Emotion Regulation and Negative Liability subscales. Co-I Miller uses this measure frequently in her work with Head Start preschoolers and finds good reliability in similar samples (α=.76-87).

Teacher-Reported. Teachers will report on child emotion regulation, another key aspect of SR at this age, using the 24-item Emotion Regulation Checklist (ERC), which generates Emotion Regulation and Negative Liability subscales. It is a 24 item, four-point Likert scale 1 = Never to 4 = Almost Always). The mean is taken and higher score indicates better emotional regulation (i.e., self-regulation behaviors). Lead (head) teachers from each classroom will receive $50.00 in the form of a gift card at bassline and post-intervention for completing this assessment on their children. (Previously approved in PROTOCOL from Co-I Miller).
Time Frame: Baseline (Month 0), post-intervention (month 9).

Cohort 2 participants will not do this measure.

**Other Variables**

The following demographical variables will be collected during the project to provide additional data to help information the findings.

1. **Play Space Assessment and POEMS**

**-The Head Start Play Space Assessment (Casey, 2007) is designed to assess Health Start in assessing the quality of outdoor play spaces for children ages 3 – 5. The tool help to identify the strengths and needs of an existing play space.** This tool is best completed during a walk-through of a play space. For each of the eleven (11) categories in the left-hand column, consider the extent to which a play space meets the given criteria. Score the play space using the rating scale below found in the center column. Circle the number that best reflects the present state of the play space. Use the right hand column to make additional comments, such as strengths, areas for improvement, high or low priority, ideas, etc. This descriptive scale examines a series of yes/no questions and 5-point Likert question for each subscale. The assessment will be measured by trained, research assistants from the Child Movement, Activity, and Developmental Health Lab.

This will be completed at the beginning and end of the academic school year.

**-POEMS Assessment Tool (DeBord, 2007)** scale that contains 56 items grouped in five domains: the “Physical Environment” takes everything from air quality to ground surface drainage into account. “Interactions” looks at how well the outdoor space facilitates a child’s interactions with peers, adults, objects, and natural materials. “Play and Learning Settings” are specifically designed play settings (much like indoor centers) with storytelling circles, elevated work spaces, moveable objects, and other suggested items. The “Program” section views the outdoor space as an extension of the classroom in all curriculum areas while “Teacher/Caregiver Role” focuses on how teachers can enhance outdoor experiences for children. This descriptive scale examine a series of yes/no questions and 5-point Likert question for each subscale. The assessment will be measured by trained, research assistants from the Child Movement, Activity, and Developmental Health Lab.

This will be completed at the beginning and end of the academic school year.

**Movement Environment Rating Scale** (MOVERS; Archer and Siraj, 2017). MOVERS is a method of measuring the quality of environment and pedagogy in which young children are encouraged to move and be physically active. It applies the methodology used in the ECERS-E and SSTEW rating scales, making it easy for educators already familiar with these well-established scales to adopt. MOVERS has four sub-scales: Curriculum, environment and resources for physical development, Pedagogy for physical development, Supporting physical activity and critical thinking, and Parents/carergivers and staff. This descriptive scale examine a series of yes/no questions and 7-point Likert question for each subscale. The assessment will be measured by trained, research assistants from the Child Movement, Activity, and Developmental Health Lab.

Time Frame: Baseline (Month 0) and follow-up assessment at the end of years 2 (e.g., beginning and end of the academic school year)

1. **Family Questionnaire.** Parents will be ask the complete a family questionnaire to provide some descriptive information about their child and family. Questions are regarding the family structure, early learning center/preschool school services, physical activity habits, sleeping habits, and screen time habits. This questionnaire will be completed during IRB consenting process by the parent/guardian. Upon return and to compensate the parent/guardian for their time, parent/guardian will receive a one-time $5.00 cash incentive (stated previous in the Demographic section). Additionally, fully completed Family Questionnaires will be entered in a drawing to win one (1) of eight (8) $50.00 Gift Cards to Wal Mart or Kroger Shopping Center.

Time Frame: Measured at baseline (Month 0).

**Photograph Protocol for Children**

During the of the study children there is an optional consent requesting that parent/guardian consent to allow their child to be photographed and understand that the photographs maybe use for educational, research, and/or publicity purposes (brochures, CMAH website). Members of the research team (Palmer, Andrews, ~~Veldman~~ & Chinn) will know which children are cleared to be photograph and be the only individuals taking the photographs of the children when engaging in the CHAMP program and when collecting some of the assessment measurement. For past research studies (i.e., at the PI’s previous university), a picture of the child engaging the in movement program was included in the Developmental Assessment Report given to parents at the end of the program for those who consented to photo release.

1. **Statistical Analysis.**

**Objective 1 (CHAMP effect on Motor Competence, Physical Activity, and Perceived Motor Competence)**

*Analysis of Primary Outcomes:* Prior to conducting outcome analysis we will examine the range and frequency distributions for all variables, and will transform variables when appropriate. Preliminary analysis will assess each outcome cross-sectionally at baseline and at all follow-up time points. We will compare study groups (i.e., CHAMP vs. Control) for comparability and change in variables over time. To study both the short-term (immediate) and long-term (sustainable) effects of CHAMP, we will assess all longitudinal outcomes by both summary statistics and descriptive data figures over the baseline, post-intervention (month 9) and follow-up at May of Year 2, 3, and 4. Since the samples are collected in different classrooms and different preschools, our data have the clustered structure, which means the within-cluster correlations likely exist. For such clustered longitudinal data, we will use primarily random effects in the following models to account for multi-level correlations among the measurements: Mixed Model Regression (MMR), Growth Curve Modeling (GCM), and Structural Equation Modeling (SEM).^70-73^ Random effects are appealing here to account for some latent mediating factors that may exist in the study. We will use MMR to determine the population-average effects of the intervention on primary and secondary outcomes (Aim 1 and 2) and SEM for mediation analysis (Aims 3). Dr. Wang (Co-Investigator) has a good deal of expertise on the relevant methodologies and extensive experience on the analysis of multi-level correlated data.

**Aim 1: Examine the immediate post-intervention effect of CHAMP (compared to control participants) on MC, PMC, and PA in preschool-age children.**

The immediate post-intervention effect of CHAMP (compared to control participants) on each outcome variable will be evaluated at post-intervention (month 9). We will examine descriptive statistics on both pre- and post-intervention measures of the outcome variables for each group. The change of MC, PMC, and PA (i.e., PA refers to time spent in MVPA) will be compared between two groups using regression models, adjusting for other confounding factors. Random effects will be included in the model to accommodate for the potential within-cluster correlation coming from the nature of how the data are collected. We will also investigate the amount of attrition from pre-intervention to post-intervention, and attempt to identify baseline (or pre-intervention) predictors of the likelihood of dropping out (as an indication of possible bias in the change estimates).

**Aim 2: Assess the sustainable effect of CHAMP (compared to control) on MC, PMC, and PA across middle childhood.**

We will use MMR to analyze the clustered longitudinal data from our RCT to assess the long-term effects of the CHAMP intervention on improving the three (3) outcome measures, MC, PMC, and PA. In this analysis, we can obtain the estimates of the long-term longitudinal intervention effect, by adjusting for confounding factors (i.e., sex). For PA, we will test for a possible delay before any significant change because of the difficulty of achieving health behavior change by adding time-lagged covariates related to behavior changes in the regression analysis. We will also conduct exploratory analyses to test interaction effects to understand potential modified intervention effects by different levels of behavior changes. The intervention is designed to promote a positive trajectory of children’s MC, PMC, and PA, and we will specifically test interactions between intervention and time, and between baseline motor skill and time, to see which baseline measure is a stronger driver of increasing physical activity.

**Aim 3: Examine and compare the immediate and long-term mediating effect of PMC on the relationship between MC and PA in preschool-age children in the CHAMP and control.**

Growth Curve Models (GCM) will enable us to understand if, and how, time-course changes in MC, PMC, and PA differ between the intervention and control groups. This model is also used to determine a time window over which the intervention effect appears stronger. We will use Structural Equation Models (SEMs) to determine which hypothesized constructs may be responsible for intervention effect on longitudinal PA. Based on the results of our previous work, for example, we hypothesize that PMC will not mediate the relationship between MC and PA at baseline, but PMC will mediate the relationship between MC and PA immediately post intervention and across the 3-yr follow-up period. We will use Mplus software to fit SEMs, and the goodness of fit of SEM models will be assessed using multiple criteria such as Chi-square to degrees of freedom (df) ratio (<2) and the Root Mean Squared Error of Approximation (RMSEA <0.05).

***Sample Size, Power Analysis and Sample Attrition.*** Sample size was calculated based on our primary endpoint, the change in PA between baseline and immediate follow-up. According to previous literature^32^, we anticipate a change score of 12.1 minutes of MVPA/day for the intervention group after 1 year, with a conservative estimate of the standard deviation (SD) as 27 MVPA/day. Such a change is accepted as a clinically meaningful change in improving PA among preschool children. To detect an adjusted 12.1 (SD=27) difference in PA after 1 year between intervention and control groups with 90% power at an alpha level of 0.05, we will need a total of 212 subjects (106 subjects per arm). To account for a total of a 30% loss to follow-up, we will recruit 298 subjects at baseline (149 subjects per arm). To maintain the statistical power as 90%, the needed sample size and the detectable differences in change of PA scores are summarized in Figure 6.^69^ Cohort feasibility data supports no difficulties identifying subjects who meet initial inclusion criteria for this study (See Approach Section C.4.4.). With the proposed sample size, the relationship between the power we can achieve for analyzing the mean change of MC score and PMC score and their corresponding expected mean difference and SD appears in Figure 7.^69^ Given expected larger effects on MC and PMC, power to detect differences across groups is larger for MC and PMC than that seen with PA.

| 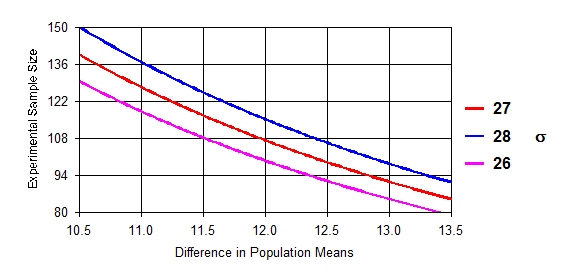  Fig 6: Sample size to achieve 90 for PA. | 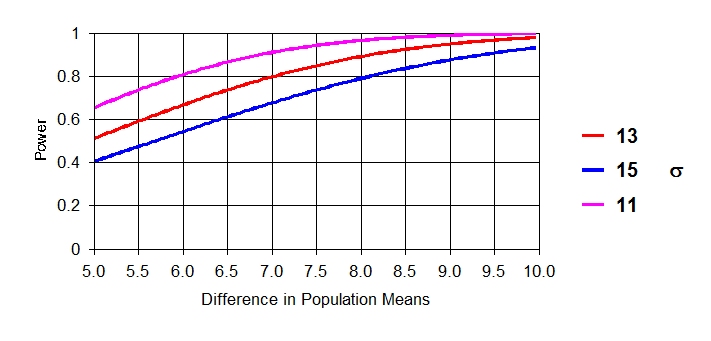  Fig 7: Power to detect changes in MC and PMC. |
| --- | --- |

**Objective 2 (CHAMP effect on Self-Regulation)**

*Data Analysis Plan.* Data analyses for this SOBC supplement will follow the funded Parent Award R01HL132979-01 strategy. We will transform skewed outcome variables as needed, run descriptive statistics, apply transformations to assure normality, and assess potential covariates to include. While maximum effort will be made to retain all subjects and minimize the amount of missing data, we anticipate there will be some data lost-to-follow-up and incomplete measures. Thus as is our practice we will address missing data by applying advanced statistical techniques, such as “multiple imputations” using PROC MI in SAS and IVEWARE SAS macro. Our overall approach is to employ multivariate analysis using a general linear modeling approach to assess associations among key variables using the appropriate models based on the distribution of the data (i.e., normal, categorical data, count data, reaction time data) and including covariates as warranted (gathered in Parent Award; e.g., child sex, race/ethnicity).

Data analyses for this SOBC supplement will follow the funded Parent Award R01HL132979-01 strategy. We will transform skewed outcome variables as needed, run descriptive statistics, apply transformations to assure normality, and assess potential covariates to include. While maximum effort will be made to retain all subjects and minimize the amount of missing data, we anticipate there will be some data lost-to-follow-up and incomplete measures. Thus as is our practice we will address missing data by applying advanced statistical techniques, such as “multiple imputations” using PROC MI in SAS and IVEWARE SAS macro. Our overall approach is to employ multivariate analysis using a general linear modeling approach to assess associations among key variables using the appropriate models based on the distribution of the data (i.e., normal, categorical data, count data, reaction time data) and including covariates as warranted (gathered in Parent Award; e.g., child sex, race/ethnicity).

**Aim 1. Test the hypothesis that CHAMP improves child SR indicators (*cognitive flexibility, working memory, behavioral inhibition, and emotion regulation*) from pre- to post-test, compared to control.**

The immediate post-intervention effect of CHAMP (compared to control participants) on each SR outcome variable will be evaluated at post-intervention (month 9). We will examine descriptive statistics for both pre- and post-intervention SR measures of the outcome variables for each group. The change in *cognitive flexibility, working memory, behavioral inhibition,* and *emotion regulation* scores will be compared between the intervention (CHAMP) and control groups using regression models, adjusting for other confounding factors. Random effects will be included in the model to accommodate for the potential within-cluster correlations due to nested classroom data. We anticipate that some children will have only partial adherence to the intervention (i.e. attend a subset of sessions), thus we will also conduct a dose-response analysis where dose corresponds to number of sessions. We will also investigate the amount of attrition from pre- to post-intervention, and attempt to identify baseline (or pre-intervention) predictors of the likelihood of dropping out (as an indication of possible bias in the change estimates).

**Aim 2. Test the hypothesis that improvement in child SR indicators (*cognitive flexibility, working memory, behavioral inhibition, and emotion regulation*) is associated with improved motor competence, perceived motor competence, and objectively-measured PA from pre- to post-test.**

We will examine strength of association between each SR variable and our outcomes of interest using bivariate analyses to compare change in SR to change in motor competence, perceived motor competence, and PA (using an alpha value of p<.05). We will also use multivariate regression models (controlling for covariates as needed) to examine the association of SR and outcomes in each group (CHAMP and control) in order to test whether the strength of association varies by intervention status.

***Sample Size, Power Analysis and Sample Attrition.*** The sample size was calculated based on our findings from preliminary data. After 9 months, we will be able to detect effects between intervention and control groups with 85% power at an alpha level of 0.05, with a total of 98 subjects (49 subjects per arm). To account for a total of a 16.5% loss at post-intervention, we will recruit 120 subjects (60 subjects per arm) at baseline.

*Missing Data*: Assuming data are Missing at Random (MAR), multiple imputation techniques will be used to replace missing data.^76^ Missing data will be assumed to be MAR if no participant demographics or primary outcomes are correlated with missingness. Mixed effects models allow for partial information to be included for individuals who may dropout prior to any post-intervention data collections. Sensitivity analysis over a range of missing data mechanisms will be performed as necessary. We expect limited missing data because the Michigan research staff will be present to supervise data collection and identify potential problems with missed data. Missing values will be multiply imputed using available covariates by sequential imputation. This approach allows optimal use of available data in analysis involving change measures.

*Early Withdrawal/Dropouts:* Measures will be taken to retain all participants overtime, however we do expect to loss participants in both the intervention and control groups overtime. A conservative estimate of this attrition rate is 30% during the study period. We plan to implement the intent-to-treat (ITT) analysis.^77^ We will make every effort to collect follow-up data at each time point from individuals who have not withdrawn from the study. Our approach has been to sponsor booster sessions as “classroom reunion/birthday parties” along with rewards and incentives to gather follow-up data. This approach has worked well for bringing kids together to reduce potential dropout.

**Data Management.** Extreme care to ensure high-quality and secure data will be exercised. All data will be stored securely at the University of Michigan. Data will have only a numerical identifier so that individual respondents cannot be identified from the data. However, it is possible to enter the ID number into the electronic subject database and thereby identify study participants. Thus, we have devised a specific procedure for limiting access to the linking information. All data will be reported as aggregate statistics and no individuals will be recognizable from the data reported. All data will be perused for consistency, errors of omission, and appropriateness of response. Unacceptable responses will be investigated with follow-up questioning, until acceptable answers are obtained. Once a coded and cleaned data file has been prepared, frequency distributions and descriptive statistics (means, standard deviations, and ranges) for each of the measured variables will be used for consistency checks and to verify the comparability of the groups. Logic check programs will be run to ensure that each data point falls within the expected range or corresponds to possible values in the codebook. These tracking system files will be maintained on a secure server at the University of Michigan. Data will be analyzed using SAS 9.3.^74,76^ All members of the study team will be required to complete the web-based University of Michigan Responsible Conduct of Research Training Program and to sign a confidentiality document stating that they understand the procedures to be followed to ensure the integrity and confidentiality of the data and the consequences of disregarding them. Careful training and ongoing monitoring of data collectors will be provided by the Principal Investigator and will follow a set of procedures used successfully in our previous studies.
