# Supplementary material for: Protocol for a cluster randomized clinical trial of a mastery-climate motor skills intervention, Children’s Health Activity and Motor Program (CHAMP), on self-regulation in preschoolers
Source: PLoS One. 2023 Mar 9;18(3):e0282199. doi: 10.1371/journal.pone.0282199 (PMC9997967; doi:10.1371/journal.pone.0282199)
Supplement: S2 File — (PDF) [file pone.0282199.s003.pdf]

ClinicalTrials.gov PRS DRAFT Receipt (Working Version)
Last Update: 11/16/2020 18:38

ClinicalTrials.gov ID: NCT03189862

Study Identification

Unique Protocol ID: HUM00133319

Brief Title: A PATH (Promoting Activity and Trajectories of Health) for Children

Official Title: A PATH (Promoting Activity and Trajectories of Health) for Children

Secondary IDs: 1R01HL132979-01 [U.S. NIH Grant/Contract Award Number]

Study Status

Record Verification: November 2020

Overall Status: Active, not recruiting

Study Start: September 11, 2017 [Actual]

Primary Completion: June 30, 2022 [Anticipated]

Study Completion: June 30, 2022 [Anticipated]

Sponsor/Collaborators

Sponsor: University of Michigan

Responsible Party: Principal Investigator
Investigator: Leah Robinson [lerobin]
Official Title: Associate Professor
Affiliation: University of Michigan

Collaborators: National Heart, Lung, and Blood Institute (NHLBI)

Oversight

U.S. FDA-regulated Drug: No

U.S. FDA-regulated Device: No

U.S. FDA IND/IDE: No

Human Subjects Review: Board Status: Approved
Approval Number: HUM00117489
Board Name: IRB-HSBS
Board Affiliation: University of MIchigan
Phone: 7349360933
Email: IRBHSBS@umich.edu
Address:

2800 Plymouth Road
Bldg 520, Room 1169

Ann Arbor, MI 48109-2800

Data Monitoring:

Study Description

Brief Summary: Physical inactivity in children is a major public health risk factor and a health objective for the nation. This study aims to investigate the short- and long-term effects of a movement and physical activity program – the Children Health and Motor Programs (CHAMP) on motor competence, perceived motor competence, and physical activity. A secondary aim of this project (i.e., Science of Behavior Change Administrative Supplement) is to examine the immediate (pre- to post-test) effects of the CHAMP intervention on self-regulation and associations between self-regulation and changes in motor competence, perceived motor competence, and physical activity. The long term goal is to provide evidence-based movement experiences during the early childhood years that promote and contribute to overall healthy growth and development.

Detailed Description: Ethnic-minorities and low income children engage in less physical activity (PA)1 and, as a result, have an increased risk of cardiovascular disease. Promoting health-enhancing and sustainable PA levels across childhood and adolescence in low income and minority populations provides important health benefits. However, most PA interventions in children have not led to long-term, sustainable PA behavior. We propose a potential limitation in PA interventions has been the lack of focus on critical developmental processes (i.e., motor development and psychological constructs including perceived competence) that are established correlates of PA and may strongly impact the long-term sustainability of children's PA behaviors.

Co-investigator, Stodden et al. proposed a developmental model hypothesizing mechanisms that promote positive longitudinal change in PA from early (≈3 yrs) to late childhood (≈12 yrs). In this model, a causal pathway that impacts PA is the development of motor competence (MC, i.e., coordination and control of human movement) and perceived motor competence (PMC; i.e., perceptions of movement capabilities). PMC is directly linked to MC and influences PA as it mediates the relationship between MC and PA across childhood. Empirical evidence supports the model's hypotheses showing that MC, PMC, and PA are positively related across childhood. Principal Investigator, Robinson found that children as early as 3 yrs old demonstrate positive associations among MC, PMC, and PA, which suggests early childhood is an optimal time to promote positive MC and PMC in order to decrease the risk of developing unhealthy PA habits.

Robinson has conceptualized and tested a theoretically grounded intervention (the Children's Health Activity Motor Program; CHAMP), which aligns with tenets of the Stodden et al. model by focusing on improving MC and PMC. Studies by the PI show highly impactful results on MC and PMC. CHAMP produced strong increases in MC (improvement to the 70th%tile, up from the 15th%tile) and PMC (30% improvement) over a 9-week intervention and results were sustained following a 12-week retention. During the 30-min intervention, CHAMP participants engaged in more PA (i.e., 50% more time) compared to children in the control.13 While these intervention results are very encouraging, there is a need to examine the long-term effects of the intervention on MC and PMC and whether improvements lead to sustained PA. To date, there have been no large-scale treatment studies that have examined the long-term effects of a MC and PA-based intervention on MC, PMC, and PA in young children.

This proposed study will address these gaps utilizing a cluster randomized control trial. The CHAMP intervention will be implemented in a high minority and low-income population, namely Head Start preschoolers (N = 300; 3.5-5

yrs old), with a 3-year follow-up to examine the immediate (pre- to post-test) and sustained (across middle childhood) impact on MC, PMC, and daily PA. 30 classes of preschool children will be randomly assigned to either the treatment (CHAMP, n = 15) or control (normal preschool free-play/recess, n = 15) conditions. The CHAMP intervention will be implemented for 30 minutes/day, 4 days/week, for 30 weeks (dose of 3000 minutes). Measurements of MC (product and process), PMC (via self-perceptions of MC), and PA (via accelerometry), will occur at baseline (month 0), post-intervention (month 9), and follow-up at May of Year 2, 3, and 4. The specific aims of this study are to:

Aim 1: Examine the immediate post-intervention effect of CHAMP (compared to control participants) on MC, PMC, and PA in preschool-age children.

Aim 2: Assess the sustainable effect of CHAMP (compared to control participants) on MC, PMC, and PA across middle childhood.

Aim 3: Examine and compare the immediate and long-term mediating effect of PMC on the relationship between MC and PA in preschool-age children in the CHAMP and control.

IMPACT: Positive findings will support the development of early childhood education MC and PA programs that promote positive and sustainable PA behaviors that contribute to healthy growth and development.

Conditions

Conditions: Motor Performance
Physical Activity

Keywords:

Study Design

Study Type: Interventional

Primary Purpose: Other

Study Phase: N/A

Interventional Study Model: Parallel Assignment

Number of Arms: 2

Masking: None (Open Label)

Allocation: Randomized

Enrollment: 300 [Actual]

Arms and Interventions

Arms	Assigned Interventions
Experimental: CHAMP<br>CHAMP, is a mastery climate motor skills interventions, that provides children the opportunity to establish behaviors that reinforces decision-making while participating in a variety of challenging movement & physical activity tasks. Duration of CHAMP is 30 min/day 4 days/week for 30 weeks. CHAMP consist of a) a 2-3 min of motor skill introductory activity that includes a group	Behavioral: Motor Skills Intervention<br>The behavioral motor skills intervention (CHAMP) is an intervention strategy that uniquely addresses differences in children's developmental levels on an individualized basis. CHAMP does not equate to a "one size fits all" approach. Children will be in an environment that promotes opportunities for them to develop improvements in motor skills based on their specific individual needs and choices. The CHAMP

Arms	Assigned Interventions
motor activity, the teaches the lesson, includes a demonstration, and understanding of developmentally appropriate learning clues b) 25 min of motor skill instruction & practice where preschoolers engage in 3-4 motor activity stations that align with the TARGET structures c) & 2-3 min motor skill closure activity that involves a review of the lesson & critical elements.	intervention promotes a mastery climate that allows each individual child to be successful and learn while promoting intrinsic motivation and autonomy. CHAMP will be implemented 4x/week for 30 minutes across ≈30 weeks for ≈3000 minutes of intervention time.
No Intervention: Control - Free Play<br>The control/free play condition will be the preschools typical activity programs (i.e., outdoor/indoor recess) and will be implemented according to the existing procedures within the preschool centers. The centers outdoor program consist of outdoor free-play activity on a large playground area with a variety of play structures (swings, slides, ladders) that promote gross movement and activity in preschoolers. For the control condition, there will be no planned instruction nor activities provided by the classroom teachers.	

Outcome Measures

Primary Outcome Measure:

1. Change in Motor Competence
   Process & product measures will concurrently assess MC. Process scores will used the Test of Gross Motor Development-3rd Edition; scores for the two subscales - locomotor & object-control will be reported as raw scores for each skill & an overall score total MC score 0 (low motor competence) – 100 (high motor competence) will also be used for data analyses when appropriate. Product scores will use kick & throw velocity, catching %; hop & jump distance & running speed. Product scores will vary for each participant (i.e., kick and throw velocity - faster score is a indicator of MC, number of successful catches out of 5 attempts - more catches indicator of MC, hop and jump distance and running speed; greater distance & speed is a better indicator of MC)

   [Time Frame: Baseline (Month 0), post-intervention (month 9)]
2. Change in Physical Activity
   ActiGraph GTX3+ tri-axial accelerometer will be used to measure the frequency, intensity and duration of PA among children at school and in free-living settings. Participants will be asked to wear the same accelerometer for 7 full days (5 week and 2 weekend days). Data will be collected at 80 hz. Cut points from a study in preschoolers by Butte et al will be applied to activity counts. This study utilized information from all three axes (versus just the vertical axis) thus time spent in sedentary, light, moderate, and vigorous categories will be defined as vector magnitudes of up to 819 (sedentary), 3907 (light), 6111 (moderate) and above 6112 for vigorous activity. The outcome will be minutes of moderate-to-vigorous PA per day, greater than 6111 activity counts.

   [Time Frame: Baseline (Month 0), post-intervention (month 9)]

Secondary Outcome Measure:

3. Duration of Changes in Motor Competence
   Process & product measures will concurrently assess MC. Process scores will used the Test of Gross Motor Development-3rd Edition; scores for the two subscales - locomotor & object-control will be reported as raw scores for each skill & an overall score total MC score 0 (low motor competence) – 100 (high motor competence) will also be used for data analyses when appropriate. Product scores will use kick & throw velocity, catching %; hop & jump distance & running speed. Product scores will vary for each participant (i.e., kick and throw velocity - faster score is a indicator of MC, number of successful catches out of 5 attempts - more catches indicator of MC, hop and jump distance and running speed; greater distance & speed is a better indicator of MC)

   [Time Frame: Follow-up measurements will be taken at the end of years 2, 3, and 4]
4. Duration of Change in Physical Activity

ActiGraph GTX3+ tri-axial accelerometer will be used to measure the frequency, intensity and duration of PA among children at school and in free-living settings. Participants will be asked to wear the same accelerometer for 7 full days (5 week and 2 weekend days). Data will be collected at 80 hz. Cut points from a study in preschoolers by Butte et al will be applied to activity counts. This study utilized information from all three axes (versus just the vertical axis) thus time spent in sedentary, light, moderate, and vigorous categories will be defined as vector magnitudes of up to 819 (sedentary), 3907 (light), 6111 (moderate) and above 6112 for vigorous activity. The outcome will be minutes of moderate-to-vigorous PA per day, greater than 6111 activity counts.

[Time Frame: Follow-up measurements will be taken at the end of years 2, 3, and 4]

5. Change in Perceived Motor Competence (Global)
   Will be assessed with the Harter & Pike Pictorial Scale of PMC and Social Acceptance (PSPCSA). The PSPCSA will measure the child's global perceived physical competence. The PSPCSA physical competence subscale consists of six items that are presented in pictures and each child will select a picture that is more like them. The six items included are swinging, climbing, tying shoe laces, skipping, running, & hopping. For both assessments, children will (1) select the picture that is most like them. One picture depicts a child who is skilled competent and the other shows a child who is not skilled; (2) focus on the designated picture and indicate whether they are just a "little bit" or "a lot" like that picture. Separate pictures for girls and boys will be used in accordance with the manual procedures. The range of scores for each item on the subscale is 1 (low competence) to 4 (high competence).

   [Time Frame: Baseline (Month 0), post-intervention (month 9)]

6. Change in Perceived Motor Competence (Fundamental)
   Will be assessed with the Perceived Fundamental Motor Skill Competence Scale (PFMSCS) is a video-based assessment that allows the child to see the entire motor skill in action rather than a static picture.The scale is the identical layout and item structure to the PSPCSA but align with the 12 fundamental motor skills of the TGMD. For the assessment, the child will select the video that is most like them. One video depicts a child who is skilled and the other shows a child who is not skilled; (2) focus on the designated video and indicate whether they are "little bit" or "a lot" like that child. Separate videos for girls and boys will be used in accordance with the manual procedures. The range of scores for each item on the subscale is 1 (low competence) to 4 (high competence).

   [Time Frame: Baseline (Month 0), post-intervention (month 9)]

7. Duration of Change in Perceived Motor Competence (Global)
   Will be assessed with the Harter & Pike Pictorial Scale of PMC and Social Acceptance (PSPCSA). The PSPCSA will measure the child's global perceived physical competence. The PSPCSA physical competence subscale consists of six items that are presented in pictures and each child will select a picture that is more like them. The six items included are swinging, climbing, tying shoe laces, skipping, running, & hopping. For both assessments, children will (1) select the picture that is most like them. One picture depicts a child who is skilled competent and the other shows a child who is not skilled; (2) focus on the designated picture and indicate whether they are just a "little bit" or "a lot" like that picture. Separate pictures for girls and boys will be used in accordance with the manual procedures. The range of scores for each item on the subscale is 1 (low competence) to 4 (high competence).

   [Time Frame: Follow-up measurements will be taken at the end of years 2, 3, and 4]

8. Duration of Change in Perceived Motor Competence (Fundamental)
   Will be assessed with the Perceived Fundamental Motor Skill Competence Scale (PFMSCS) is a video-based assessment that allows the child to see the entire motor skill in action rather than a static picture.The scale is the identical layout and item structure to the PSPCSA but align with the 12 fundamental motor skills of the TGMD. For the assessment, the child will select the video that is most like them. One video depicts a child who is skilled and the other shows a child who is not skilled; (2) focus on the designated video and indicate whether they are "little bit" or "a lot" like that child. Separate videos for girls and boys will be used in accordance with the manual procedures. The range of scores for each item on the subscale is 1 (low competence) to 4 (high competence).

   [Time Frame: Follow-up measurements will be taken at the end of years 2, 3, and 4]

9. Change in Cognitive Flexibility
   The Dimensional Change Cart Sort (DCCS) Task is a measure of cognitive flexibility. The task requires children to learn and remember a rule and apply it one way, and then apply a new rule as instructions change. Children match pictures that vary by two dimensions, shape (rabbit vs. boat) and color (brown vs. white). Children complete trials with one set of instructions, then another, and are encouraged to go as fast as they can without making mistakes. 0 = did not pass color sort (Less than 5/6 items correct on color sort)

   1 = pass of color sort, fail on shape sort)

[Time Frame: Baseline (Month 0), post-intervention (month 9)]

10. Change in Working Memory

    Working Memory will be assessed using a visual-spatial working memory task which requires children to accurately recall information they have seen before. Children are shown visual information (stickers on the body of a cartoon character) and next shown the character without the stickers, and asked to identify these locations after a brief retention interval. Test trials increase in difficulty (i.e., WM demand) as the task progresses. Test continues until a max. of 8 levels or failure on all 3 trials at the same level of difficulty. For working memory, faster, more accurate responses reflect better self-regulation.WM capacity was indexed by a point score calculated as follows: beginning from Level

    1, one point for each consecutive level in which at least two of the three trials were performed accurately, plus 1/3 of a point for all correct trials thereafter.

    [Time Frame: Baseline (Month 0), post-intervention (month 9)]

11. Change in Behavioral Inhibition

    The Head-Toes-Knees-Shoulders Task (HTKS; 10 min.) will be used to measure behavioral inhibition. It reflects a child's ability to remember commands and to behaviorally inhibit a pre-potent response in favor of a less-dominant response. A child is trained to perform actions in response to an examiner's commands (e.g., "touch your toes") then asked to do the opposite (e.g., touch toes when instruction is "touch your head"). Scores across trials are summed to reflect self-regulation. The task has begins with 6 practice items and between the first and second set of items there are 5 more practice trials. The score range is 0-40; higher score equal better behavioral inhibition.

    [Time Frame: Baseline (Month 0), post-intervention (month 9)]

12. Change in Observed Self-Regulation

    Observed self-regulation will also evaluate the child ability to stay on-task during HTKS as an aspect of self-regulation. Child compliance and engagement will also be rated using the Child Assessor Report which has been used in prior SR trials. This 10 item questionnaire is scored on a 0-3 scale. The mean is taken and higher score indicates better observed self-regulation behaviors.

    [Time Frame: Baseline (Month 0), post-intervention (month 9)]

13. Change in Teacher Reported Self-Regulation

    Teacher-Reported. Teachers will report on child emotion regulation, another key aspect of SR at this age, using the 24-item Emotion Regulation Checklist (ERC), which generates Emotion Regulation and Negative Lability subscales. It is a 24 item, four-point Likert scale 1 = Never to 4 = Almost Always). The mean is taken and higher score indicates better emotional regulation (i.e., self-regulation behaviors).

    [Time Frame: Baseline (Month 0), post-intervention (month 9)]

Other Pre-specified Outcome Measures:

14. Body mass index

    Body mass index (BMI) is a weight outcome that will be collected to calibrate actigraphs and as a confounding variable. The measure will be collected with the standard procedures and all scales will be calibrated before testing. Height and weight will be used to calculate BMI. Height will be measured to the nearest unit (in centimeters) in bare feet with the child standing upright against a portable stadiometer (Charder HM200P PortStad, Taiwan ROC). Weight will be measured to the nearest unit (in kilograms) with heavy clothes removed (ie, wearing pants and shirt) using a portable electric weight scale (Seca 813; Seca North America). Body mass index (BMI) will be calculated based on age-specific and sex-specific CDC (Centers for Disease Control and Prevention) growth charts and transformed into BMI z-scores for analyses using the following formula ~ weight (kg) / [height (m)]2.

    [Time Frame: Baseline (Month 0), post-intervention (month 9), Follow-up measurements will be taken at the end of years 2, 3, and 4]

15. Waist circumference

    Waist circumference is a weight outcome that will be collected as a confounding variable. The measure will be collected with the standard procedures - a non-elastic tape measure (Seca 201; Seca North America, Chino, California, USA) at the umbilicus.87 The measurement will be taken as the children complete a breath (ie, exhaled) to the nearest unit (in centimeters).

    [Time Frame: Baseline (Month 0), post-intervention (month 9), Follow-up measurements will be taken at the end of years 2, 3, and 4]

16. Body fat percentage

Body fat percentage is a weight outcome that will be collected as a confounding variable. A Tanita SC-331S foot-to-foot body composition analyzer (Tanita Cooperation, Tokyo, Japan) will be used to assess bioelectrical impedance. Measurements were collected using the standard setting after manually imputing the measured height, gender, and age of the subject. The children were bare foot and wore minimal clothing and were instructed to standstill with their feet touching all four metal plates. BF% was then estimated using the in-built Tanita equations. FM (kg) was calculated as: BF% divided by 100 and then multiplied by body weight and FFM (kg) was subsequently calculated as the difference of body weight and FM.

[Time Frame: Baseline (Month 0), post-intervention (month 9), Follow-up measurements will be taken at the end of years 2, 3, and 4]

Eligibility

Minimum Age: 42 Months

Maximum Age: 60 Months

Sex: All

Gender Based: No

Accepts Healthy Volunteers: Yes

Criteria: Inclusion Criteria:

1. Recruitment is limited to two specific schools located in the Detroit Metro area.
2. Participants must be in the last year of preschool entering Kindergarten the next academic year is eligible to to participate in this study.

Exclusion Criteria:

1. Any preschooler with a severe developmental, cognitive, and/or physical disability as noted by school records is eligible to participate in this study but data will not be collected.

Contacts/Locations

Central Contact Person: Elizabeth Tropiano
Telephone: 734-615-5373
Email: etropian@umich.edu

Central Contact Backup:

Study Officials: Leah E Robinson, PhD
Study Principal Investigator
University of Michigan

Locations: **United States, Michigan**
University of Michigan
Ann Arbor, Michigan, United States, 48109
Contact: Leah E Robinson, PhD lerobin@umich.edu
Principal Investigator: Leah E Robinson, PhD

IPDSharing

Plan to Share IPD: No

References

Citations: Robinson LE. Effect of a mastery climate motor program on object control skills and perceived physical competence in preschoolers. Res Q Exerc Sport. 2011 Jun;82(2):355-9. PubMed 21699116

Robinson LE, Rudisill ME, Goodway JD. Instructional climates in preschool children who are at-risk. Part II: perceived physical competence. Res Q Exerc Sport. 2009 Sep;80(3):543-51. PubMed 19791640

Robinson LE. The relationship between perceived physical competence and fundamental motor skills in preschool children. Child Care Health Dev. 2011 Jul;37(4):589-96. doi: 10.1111/j.1365-2214.2010.01187.x. Epub 2010 Dec 9. PubMed 21143273

Robinson LE, Goodway JD. Instructional climates in preschool children who are at-risk. Part I: object-control skill development. Res Q Exerc Sport. 2009 Sep;80(3):533-42. PubMed 19791639

Ulrich DA. Test of gross motor development-3. Austin, TX: Prod-Ed; 2015.

Stodden DF, Langendorfer SJ, Fleisig GS, Andrews JR. Kinematic constraints associated with the acquisition of overarm throwing part I: step and trunk actions. Res Q Exerc Sport. 2006 Dec;77(4):417-27. PubMed 17243217

Stodden DF, Langendorfer SJ, Fleisig GS, Andrews JR. Kinematic constraints associated with the acquisition of overarm throwing part II: upper extremity actions. Res Q Exerc Sport. 2006 Dec;77(4):428-36. PubMed 17243218

Harter S, Pike R. The pictorial scale of perceived competence and social acceptance for young children. Child Dev. 1984 Dec;55(6):1969-82. PubMed 6525886

Harter S. Manual for the Self-Perception Profile for Children. Denver, CO: University of Denver; 1985.

Trost SG, McIver KL, Pate RR. Conducting accelerometer-based activity assessments in field-based research. Med Sci Sports Exerc. 2005 Nov;37(11 Suppl):S531-43. Review. PubMed 16294116

Choi L, Liu Z, Matthews CE, Buchowski MS. Validation of accelerometer wear and nonwear time classification algorithm. Med Sci Sports Exerc. 2011 Feb;43(2):357-64. doi: 10.1249/MSS.0b013e3181ed61a3. PubMed 20581716

Willoughby M, Blair C. Test-retest reliability of a new executive function battery for use in early childhood. Child Neuropsychol. 2011;17(6):564-79. doi: 10.1080/09297049.2011.554390. Epub 2011 Jun 30. PubMed 21714751

Raver CC, Jones SM, Li-Grining C, Zhai F, Bub K, Pressler E. CSRP's Impact on low-income preschoolers' preacademic skills: self-regulation as a mediating mechanism. Child Dev. 2011 Jan-Feb;82(1):362-78. doi: 10.1111/j.1467-8624.2010.01561.x. PubMed 21291447

Raver CC, Jones SM, Li-Grining C, Zhai F, Bub K, Pressler E. CSRP's Impact on low-income preschoolers' preacademic skills: self-regulation as a mediating mechanism. Child Dev. 2011 Jan-Feb;82(1):362-78. doi: 10.1111/j.1467-8624.2010.01561.x. PubMed 21291447

Links:

Available IPD/Information:

U.S. National Library of Medicine | U.S. National Institutes of Health | U.S. Department of Health & Human Services
