# Supplementary material for: Protocol for a cluster randomized clinical trial of a mastery-climate motor skills intervention, Children’s Health Activity and Motor Program (CHAMP), on self-regulation in preschoolers
Source: PLoS One. 2023 Mar 9;18(3):e0282199. doi: 10.1371/journal.pone.0282199 (PMC9997967; doi:10.1371/journal.pone.0282199)
Supplement: S3 File — (PDF) [file pone.0282199.s004.pdf]

eRESEARCH | REGULATORY MANAGEMENT

Date: Tuesday, October 26, 2021 11:11:27 AM

Print Close

View: 01. General Study Information
Section: 01. General Study Information

01. General Study Information

The forms menu on the left displays all sections and pages of the application. Pages in **bold** are required. Pages in *italics* may not apply to your project. Use the "Continue" button to advance through the smartform, as it will only display the sections that must be completed.

All questions marked with a red asterisk (*) are required. Questions without a red asterisk may or may not be required, depending on their relevance to the study.

1.1* Study Title:

A PATH (Promoting Activity and Trajectories of Health) for Children (Phase 1)

1.1.1 Full Study Title:

A PATH (Promoting Activity and Trajectories of Health) for Children (Phase 1) / SOBC

1.1.2 If there are other U-M studies related to this project, enter the eResearch ID number (HUM#) or IRBMED Legacy study number. Examples of related projects include, but are not limited to:

- Projects funded under the same grant
- IRBMED Legacy study being migrated into eResearch
- Previously approved Umbrella applications (such as Center Grants or approvals for release of funding)
- Previously approved projects for which this is a follow up study

HUM00117489

1.1.3* Does this application include the study of COVID-19?

For example:

- **testing or studying the COVID-19 virus,**
- **exploring treatment options,**
- **studying the impact of the COVID-19 pandemic (This could included epidemiological, social, behavioral, or educational research).**

Note: Answer "Yes" only if this project includes the study of COVID-19. Inclusion of study procedures solely intended to allow the research to be conducted under pandemic constraints, such as remote interactions with subjects, remote consenting, or at-home drug delivery are not considered the study of COVID-19.

○ Yes ○ No

1.2* Principal Investigator:

Leah Robinson
Note: If the user is not in the system, you may Create A New User Account...

1.3 Study Team Members:

Study Team Member	Study Team Role	Appointment Dept	Appointment Selection Complete?	Student	Friend Account	COI Review Required	Edit Rights	Accepted Role?	PEERRS Human Subjects?
Leah Robinson	PI	School of Kinesiology	Yes	no	No	no	yes	N/A	yes
Natalie Colabianchi	Co-Investigator	School of Kinesiology	Yes	no	No	no	yes	Yes	yes
Alison Miller	Co-Investigator	Hlth Behavior & Hlth Ed Dept	Yes	no	No	no	yes	Yes	yes
Dale Ulrich	Co-Investigator	School of Kinesiology	Yes	no	No	no	yes	Yes	yes
David Stodden	Other		N/A	no	Yes	no	no	Yes	no

Study Team Member	Study Team Role	Appointment Dept	Appointment Selection Complete?	Student	Friend Account	COI Review Required	Edit Rights	Accepted Role?	PEERRS Human Subjects?
Bryan Terlizzi	Other		N/A	no	Yes	no	no	Yes	yes
Elizabeth Webster	Other		N/A	no	Yes	no	no	Yes	yes
Lu Wang	Biostatistician	Biostatistics Department	Yes	no	No	no	yes	Yes	yes
Cathy Antonakos	Research Staff	School of Kinesiology	Yes	no	No	no	no	Yes	yes
Katherine Chinn	Research Staff	School of Kinesiology	Yes	no	No	no	no	Yes	yes
Kara Palmer	Research Staff	School of Kinesiology	Yes	no	No	no	no	Yes	yes
Katherine Scott-Andrews	Research Staff	School of Kinesiology	Yes	no	No	no	no	Yes	yes
Matthew Stewart	Research Staff		No	yes	No	no	no	Yes	yes
Carissa Wengrovius	Research Staff		N/A	yes	No	no	no	Yes	yes
Indica Sur	Administrative Staff	School of Kinesiology	Yes	no	No		yes	N/A	yes

1.8* Project Summary:

Physical inactivity in children is a major public health risk factor and national health objective. This National Institutes of Health application 1 R01 HL132979-01 entitled “A PATH (Promoting Activity and Trajectories of Health) for Children” will investigate the immediate and long-term effects of a motor skills - the Children’s Health Activity Motor Program (CHAMP) on motor competence, perceived motor competence, and physical activity in preschool-age children and the Science of Behavior Change Supplement will examine the immediate (pre- to post-test) effects of the CHAMP intervention on self-regulation (SR). The specific aims of the study are:
1) examine the immediate post-intervention effect of CHAMP (compared to control participants) on motor competence, perceived motor competence, physical activity, self-regulation in preschool-age children;
2) assess the sustainable effect of CHAMP (compared to control participants) on motor competence, perceived motor competence, and physical activity across middle childhood; and
3) examine and compare the immediate and long-term mediating effects of perceived motor competence on the relationship between motor competence and physical activity in preschool-age children.
This study is a cluster randomized control trial in preschoolers (N = 300; 3.5-5 years old). Thirty classes of preschool children will be randomly assigned to either the treatment (CHAMP, n = 15) or control (normal preschool free-play/recess, n = 15) condition. The CHAMP intervention will be implemented for 4, 30 minutes sessions per week or 3, 40 minute session per week for 30 weeks, depending on the needs of the school schedule. Data will be collected on: a) 7-day physical activity levels using accelerometry, b) process- and product-oriented motor skill competence, c) perceived motor competence, and d) self-regulation

IMPACT. Positive findings will provide initial support for CHAMP as an effective intervention that contributes to healthy growth and development in children and early childhood education.

1.9* Select the appropriate IRB:

Health Sciences and Behavioral Sciences

1.10* *Estimated* Study Start Date (Not required for IRBMED): (mm/dd/yyyy)

7/22/2018

1.11* *Estimated* Duration of Study:

7/22/2023

View: VIEW000072_customAttributes._attribute186_Study Team Detail
Section: 01. General Study Information

Study Team Detail

1.4 Team Member:

Leah Robinson

Preferred email: lerobin@umich.edu
Business phone 734-647-7645
Business address: School of Kinesiology School of Kinesiology Bldg #1054 48109-1048

1.5 Function with respect to project:

PI

1.6 Allow this person to EDIT the application, including any supporting documents/stipulations requested during the review process:

Yes

Credentials: Required for PI, Co-Is and Faculty Advisors

Upload or update your CV, resume, or biographical sketch.

Name	Version
Updated Robinson Biosketch June 2018.docx(0.01)	0.01

Conflict of Interest Detail: Required for all roles except Administrative Staff

Current Disclosure Status in M-Inform: *This study team member has indicated in M-inform that they do not have any outside interests to disclose.*

D1 Do you or your family members have an outside activity, relationship, or interest with a non-UM entity, where the non-UM entity:

- Provides financial or non-financial support for this project;
- Supplies a product used in this project (e.g., an app, device, compound, drug, software, survey, evaluation) either for free or at a cost (e.g., purchased);
- Holds an option or license to intellectual property used in this project (e.g., a device, compound, drug, software, survey, evaluation, code, data, schematics, algorithms) that you or your family member developed;
- Will perform work on this project (e.g., subcontract, service agreement, unfunded agreement); or
- Has a financial stake in the outcome of this research?

No

D2 If "Yes" to the question above, provide the name of the outside entity or entities and a brief description of the interest/relationship(s):

View: VIEW000072_customAttributes._attribute186_Study Team Detail
Section: 01. General Study Information

Study Team Detail

1.4 Team Member:

Natalie Colabianchi

Preferred email: colabian@umich.edu
Business phone 734-647-3543
Business address: School of Kinesiology School of Kinesiology Bldg #1058 48109-1048

1.5 Function with respect to project:

Co-Investigator

1.6 Allow this person to EDIT the application, including any supporting documents/stipulations requested during the review process:

Yes

1.7 Include this person on all correspondences regarding this application: (Note: This will include all committee correspondence, decision outcomes, renewal notices, and adverse event submissions.)

Yes

Credentials: Required for PI, Co-Is and Faculty Advisors

Upload or update your CV, resume, or biographical sketch.

Name	Version
CV(0.07)	0.07

Conflict of Interest Detail: Required for all roles except Administrative Staff

Current Disclosure Status in M-Inform: *This study team member has disclosed outside interest(s) or relationship(s) in M-Inform.*

D1 Do you or your family members **have an** outside activity, relationship, or interest **with a** non-UM entity**, where the non-UM entity:**

- Provides financial or non-financial support for this project;
- Supplies a product used in this project (e.g., an app, device, compound, drug, software, survey, evaluation) either for free or at a cost (e.g., purchased);
- Holds an option or license to intellectual property used in this project (e.g., a device, compound, drug, software, survey, evaluation, code, data, schematics, algorithms) that you or your family member developed;
- Will perform work on this project (e.g., subcontract, service agreement, unfunded agreement); or
- Has a financial stake in the outcome of this research?

No

D2 If "Yes" to the question above, provide the name of the outside entity or entities and a brief description of the interest/relationship(s):

View: VIEW000072_customAttributes._attribute186_Study Team Detail
Section: 01. General Study Information

Study Team Detail

1.4 Team Member:

Alison Miller

Preferred email: alimill@umich.edu
Business phone 734-615-7459
Business address: Hlth Behavior/Hlth Education 3718 SPH I 48109-2029

1.5 Function with respect to project:

Co-Investigator

1.6 Allow this person to EDIT the application, including any supporting documents/stipulations requested during the review process:

Yes

1.7 Include this person on all correspondences regarding this application: (Note: This will include all committee correspondence, decision outcomes, renewal notices, and adverse event submissions.)

Yes

Credentials: Required for PI, Co-Is and Faculty Advisors

Upload or update your CV, resume, or biographical sketch.

Name	Version
CV-AMiller Sept_2021_ForIRB.docx(0.01)	0.01
Miller CV(0.04)	0.04

Conflict of Interest Detail: Required for all roles except Administrative Staff

Current Disclosure Status in M-Inform: *This study team member has disclosed outside interest(s) or relationship(s) in M-Inform.*

D1 Do you or your family members **have an** outside activity, relationship, or interest **with a** non-UM entity**, where the non-UM entity:**

- Provides financial or non-financial support for this project;
- Supplies a product used in this project (e.g., an app, device, compound, drug, software, survey, evaluation) either for free or at a cost (e.g., purchased);
- Holds an option or license to intellectual property used in this project (e.g., a device, compound, drug, software, survey, evaluation, code, data, schematics, algorithms) that you or your family member developed;
- Will perform work on this project (e.g., subcontract, service agreement, unfunded agreement); or
- Has a financial stake in the outcome of this research?

No

D2 If "Yes" to the question above, provide the name of the outside entity or entities and a brief description of the interest/relationship(s):

View: VIEW000072_customAttributes._attribute186_Study Team Detail
Section: 01. General Study Information

Study Team Detail

1.4 Team Member:

Dale Ulrich

Preferred email: ulrichd@umich.edu
Business phone
Business address: School of Kinesiology Bldg #4190 48109-1048

1.5 Function with respect to project:

Co-Investigator

1.6 Allow this person to EDIT the application, including any supporting documents/stipulations requested during the review process:

Yes

1.7 Include this person on all correspondences regarding this application: (Note: This will include all committee correspondence, decision outcomes, renewal notices, and adverse event submissions.)

Yes

Credentials: Required for PI, Co-Is and Faculty Advisors

Upload or update your CV, resume, or biographical sketch.

Name	Version
DUlrichCV_October 2018.docx(0.01)	0.01

Conflict of Interest Detail: Required for all roles except Administrative Staff

Current Disclosure Status in M-Inform: *This study team member has not yet disclosed in M-Inform.*

D1 Do you or your family members **have an** outside activity, relationship, or interest **with a** non-UM entity**, where the non-UM entity:**

- Provides financial or non-financial support for this project;
- Supplies a product used in this project (e.g., an app, device, compound, drug, software, survey, evaluation) either for free or at a cost (e.g., purchased);
- Holds an option or license to intellectual property used in this project (e.g., a device, compound, drug, software, survey, evaluation, code, data, schematics, algorithms) that you or your family member developed;
- Will perform work on this project (e.g., subcontract, service agreement, unfunded agreement); or
- Has a financial stake in the outcome of this research?

No

D2 If “Yes” to the question above, provide the name of the outside entity or entities and a brief description of the interest/relationship(s):

View: VIEW000072_customAttributes._attribute186_Study Team Detail
Section: 01. General Study Information

Study Team Detail

1.4 Team Member:

David Stodden

Preferred email: stodden@mailbox.sc.edu
Business phone 803-777-9882
Business address: University of South Carolina Office 225, 1530 Wheat Street 29208

1.5 Function with respect to project:

Other

1.6 Allow this person to EDIT the application, including any supporting documents/stipulations requested during the review process:

No

1.7 Include this person on all correspondences regarding this application: (Note: This will include all committee correspondence, decision outcomes, renewal notices, and adverse event submissions.)

Yes

Credentials: Required for PI, Co-Is and Faculty Advisors

Upload or update your CV, resume, or biographical sketch.

Name	Version
Stodden CV(0.01)	0.01

Financial Interest and Relationship Screening Questions for Study Team Members Not Affiliated with the University of Michigan: Required for all roles except Administrative Staff

Below, you be asked several questions intended to identify Financial Interests and relationships that may be relevant to **THIS RESEARCH**.

If you are an investigator on this project who is working on behalf of a company/organization **directly** involved in this project (i.e., funding the research, contracted to perform a portion of the research, providing the drug/device/app being studied), your salary from that company/organization **is not** considered a Financial Interest (note, ownership of the company would be).

In relation to **THIS RESEARCH**, over the past 12 months, do you, your spouse, domestic partner, or dependent children have or anticipate having any of the following:

F1. Any *activities* or *relationships* with an entity (excluding entities directly involved in the project), whether paid or unpaid, where that entity's financial interests could be affected by this research? Examples include service on a board of directors, service on a scientific advisory board, consultant, officer, manager, or partner.

No

F2. An *Equity Interest* in any publicly traded or privately-owned entity whose financial interests could be affected by this research, including but not limited to shares of stock or stock options? DO NOT include equity held in a mutual, pension, or investment fund over which you have no control with regard to investment decisions.

No

F3. An investorship or ownership interest in any *Intellectual Property (IP)* that is being tested, evaluated, developed in, or its commercial value will be affected by this research? This

includes IP that is the subject of a copyright, issued patent, or a patent application (regardless of whether it has been licensed or optioned). If relevant to this research, you should also consider companies that compete commercially with the research sponsor or the manufacturer of the study drug, device, or other investigational item if you know that the competitor's Financial Interests would reasonably appear to be affected by this research.

No

F4. Any payments over $5,000 (USD) received for the past 12 months (apart from any payments from the University of Michigan), including salary, honoraria, fees, or other forms of compensation or anything of value, from any entity (excluding entities directly involved in the project) that has a financial interest in this research?

No

F5. If any of the above is answered "yes", you must complete this form **and upload the completed form below.**

View: VIEW000072_customAttributes._attribute186_Study Team Detail
Section: 01. General Study Information

Study Team Detail

1.4 Team Member:

Bryan Terlizzi

Preferred email: terlizzb@email.sc.edu
Business phone
Business address: 1300 Wheat Street 29208

1.5 Function with respect to project:

Other
Assist with data collection

1.6 Allow this person to EDIT the application, including any supporting documents/stipulations requested during the review process:

No

1.7 Include this person on all correspondences regarding this application: (Note: This will include all committee correspondence, decision outcomes, renewal notices, and adverse event submissions.)

No

Credentials: Required for PI, Co-Is and Faculty Advisors

Upload or update your CV, resume, or biographical sketch.

Name	Version
Bryan Terlizzi- Resume(0.01)	0.01

Financial Interest and Relationship Screening Questions for Study Team Members Not Affiliated with the University of Michigan: Required for all roles except Administrative Staff

Below, you be asked several questions intended to identify Financial Interests and relationships that may be relevant to **THIS RESEARCH**.

If you are an investigator on this project who is working on behalf of a company/organization **directly** involved in this project (i.e., funding the research, contracted to perform a portion of the research, providing the drug/device/app being studied), your salary from that company/organization **is not** considered a Financial Interest (note, ownership of the company would be).

In relation to **THIS RESEARCH**, over the past 12 months, do you, your spouse, domestic partner, or dependent children have or anticipate having any of the following:

F1. Any *activities* or *relationships* with an entity (excluding entities directly involved in the project), whether paid or unpaid, where that entity's financial interests could be affected by this research? Examples include service on a board of directors, service on a scientific advisory board, consultant, officer, manager, or partner.

No

F2. An *Equity Interest* in any publicly traded or privately-owned entity whose financial interests could be affected by this research, including but not limited to shares of stock or stock options? DO NOT include equity held in a mutual, pension, or investment fund over which you have no control with regard to investment decisions.

No

F3. An investorship or ownership interest in any *Intellectual Property (IP)* that is being tested,

evaluated, developed in, or its commercial value will be affected by this research? This includes IP that is the subject of a copyright, issued patent, or a patent application (regardless of whether it has been licensed or optioned). If relevant to this research, you should also consider companies that compete commercially with the research sponsor or the manufacturer of the study drug, device, or other investigational item if you know that the competitor's Financial Interests would reasonably appear to be affected by this research.

No

F4. Any payments over $5,000 (USD) received for the past 12 months (apart from any payments from the University of Michigan), including salary, honoraria, fees, or other forms of compensation or anything of value, from any entity (excluding entities directly involved in the project) that has a financial interest in this research?

No

F5. If any of the above is answered "yes", you must complete this form **and upload the completed form below.**

View: VIEW000072_customAttributes._attribute186_Study Team Detail
Section: 01. General Study Information

Study Team Detail

1.4 Team Member:

Elizabeth Webster

Preferred email:	ewebster@augusta.edu
Business phone	7067218305
Business address:	1120 15th Street 30912

1.5 Function with respect to project:

Other
Executed agreement with Augusta University for Elizabeth Webster's analysis of identifiable video. Dr. Elizabeth Kip Webster is an Assistant Professor with the Institute of Public and Preventive Health at Augusta University

1.6 Allow this person to EDIT the application, including any supporting documents/stipulations requested during the review process:

No

1.7 Include this person on all correspondences regarding this application: (Note: This will include all committee correspondence, decision outcomes, renewal notices, and adverse event submissions.)

No

Credentials: Required for PI, Co-Is and Faculty Advisors

Upload or update your CV, resume, or biographical sketch.

Name	Version
There are no items to display	

Financial Interest and Relationship Screening Questions for Study Team Members Not Affiliated with the University of Michigan: Required for all roles except Administrative Staff

Below, you be asked several questions intended to identify Financial Interests and relationships that may be relevant to **THIS RESEARCH**.

If you are an investigator on this project who is working on behalf of a company/organization **directly** involved in this project (i.e., funding the research, contracted to perform a portion of the research, providing the drug/device/app being studied), your salary from that company/organization **is not** considered a Financial Interest (note, ownership of the company would be).

In relation to **THIS RESEARCH**, over the past 12 months, do you, your spouse, domestic partner, or dependent children have or anticipate having any of the following:

F1. Any *activities* or *relationships* with an entity (excluding entities directly involved in the project), whether paid or unpaid, where that entity's financial interests could be affected by this research? Examples include service on a board of directors, service on a scientific advisory board, consultant, officer, manager, or partner.

No

F2. An *Equity Interest* in any publicly traded or privately-owned entity whose financial interests could be affected by this research, including but not limited to shares of stock or stock options? DO NOT include equity held in a mutual, pension, or investment fund over which you have no control with regard to investment decisions.

No

F3. An investorship or ownership interest in any *Intellectual Property (IP)* that is being tested,

evaluated, developed in, or its commercial value will be affected by this research? This includes IP that is the subject of a copyright, issued patent, or a patent application (regardless of whether it has been licensed or optioned). If relevant to this research, you should also consider companies that compete commercially with the research sponsor or the manufacturer of the study drug, device, or other investigational item if you know that the competitor's Financial Interests would reasonably appear to be affected by this research.

No

F4. Any payments over $5,000 (USD) received for the past 12 months (apart from any payments from the University of Michigan), including salary, honoraria, fees, or other forms of compensation or anything of value, from any entity (excluding entities directly involved in the project) that has a financial interest in this research?

No

F5. If any of the above is answered "yes", you must complete this form **and upload the completed form below.**

View: VIEW000072_customAttributes._attribute186_Study Team Detail
Section: 01. General Study Information

Study Team Detail

1.4 Team Member:

Lu Wang

Preferred email:	luwang@umich.edu
Business phone	734-647-6935
Business address:	Biostatistics 4132 SPH II 48109-2029

1.5 Function with respect to project:

Biostatistician

1.6 Allow this person to EDIT the application, including any supporting documents/stipulations requested during the review process:

Yes

1.7 Include this person on all correspondences regarding this application: (Note: This will include all committee correspondence, decision outcomes, renewal notices, and adverse event submissions.)

Yes

Credentials: Required for PI, Co-Is and Faculty Advisors

Upload or update your CV, resume, or biographical sketch.

Name	Version
CV_Lu Wang(0.06)	0.06

Conflict of Interest Detail: Required for all roles except Administrative Staff

Current Disclosure Status in M-Inform: *This study team member has indicated in M-inform that they do not have any outside interests to disclose.*

D1 Do you or your family members **have an** outside activity, relationship, or interest **with a** non-UM entity**, where the non-UM entity:**

- Provides financial or non-financial support for this project;
- Supplies a product used in this project (e.g., an app, device, compound, drug, software, survey, evaluation) either for free or at a cost (e.g., purchased);
- Holds an option or license to intellectual property used in this project (e.g., a device, compound, drug, software, survey, evaluation, code, data, schematics, algorithms) that you or your family member developed;
- Will perform work on this project (e.g., subcontract, service agreement, unfunded agreement); or
- Has a financial stake in the outcome of this research?

No

D2 If “Yes” to the question above, provide the name of the outside entity or entities and a brief description of the interest/relationship(s):

View: VIEW000072_customAttributes._attribute186_Study Team Detail
Section: 01. General Study Information

Study Team Detail

1.4 Team Member:

Cathy Antonakos

Preferred email: cathya@umich.edu
Business phone 734-763-2812
Business address: School of Kinesiology School of Kinesiology Bldg #1055 48109-1048

1.5 Function with respect to project:

Research Staff

1.6 Allow this person to EDIT the application, including any supporting documents/stipulations requested during the review process:

No

1.7 Include this person on all correspondences regarding this application: (Note: This will include all committee correspondence, decision outcomes, renewal notices, and adverse event submissions.)

No

Credentials: Required for PI, Co-Is and Faculty Advisors

Upload or update your CV, resume, or biographical sketch.

Name	Version
clavita.pdf(0.01)	0.01

Conflict of Interest Detail: Required for all roles except Administrative Staff

Current Disclosure Status in M-Inform: *This study team member has disclosed outside interest(s) or relationship(s) in M-Inform.*

D1 Do you or your family members **have an** outside activity, relationship, or interest **with a** non-UM entity**, where the non-UM entity:**

- Provides financial or non-financial support for this project;
- Supplies a product used in this project (e.g., an app, device, compound, drug, software, survey, evaluation) either for free or at a cost (e.g., purchased);
- Holds an option or license to intellectual property used in this project (e.g., a device, compound, drug, software, survey, evaluation, code, data, schematics, algorithms) that you or your family member developed;
- Will perform work on this project (e.g., subcontract, service agreement, unfunded agreement); or
- Has a financial stake in the outcome of this research?

No

D2 If “Yes” to the question above, provide the name of the outside entity or entities and a brief description of the interest/relationship(s):

View: VIEW000072_customAttributes._attribute186_Study Team Detail
Section: 01. General Study Information

Study Team Detail

1.4 Team Member:

Katherine Chinn

Preferred email: katchinn@umich.edu
Business phone
Business address: School of Kinesiology 401 Washtenaw Avenue 1271 CCRB 48109-2014

1.5 Function with respect to project:

Research Staff

1.6 Allow this person to EDIT the application, including any supporting documents/stipulations requested during the review process:

No

1.7 Include this person on all correspondences regarding this application: (Note: This will include all committee correspondence, decision outcomes, renewal notices, and adverse event submissions.)

Yes

Credentials: Required for PI, Co-Is and Faculty Advisors

Upload or update your CV, resume, or biographical sketch.

Name	Version
There are no items to display	

Conflict of Interest Detail: Required for all roles except Administrative Staff

Current Disclosure Status in M-Inform: *This study team member has not yet disclosed in M-Inform.*

D1 Do you or your family members have an outside activity, relationship, or interest with a non-UM entity, where the non-UM entity:

- Provides financial or non-financial support for this project;
- Supplies a product used in this project (e.g., an app, device, compound, drug, software, survey, evaluation) either for free or at a cost (e.g., purchased);
- Holds an option or license to intellectual property used in this project (e.g., a device, compound, drug, software, survey, evaluation, code, data, schematics, algorithms) that you or your family member developed;
- Will perform work on this project (e.g., subcontract, service agreement, unfunded agreement); or
- Has a financial stake in the outcome of this research?

No

D2 If "Yes" to the question above, provide the name of the outside entity or entities and a brief description of the interest/relationship(s):

View: VIEW000072_customAttributes._attribute186_Study Team Detail
Section: 01. General Study Information

Study Team Detail

1.4 Team Member:

Kara Palmer

Preferred email:
Business phone 734-647-0284
Business address: School of Kinesiology School of Kinesiology Bldg #4130 48109-1048

1.5 Function with respect to project:

Research Staff

1.6 Allow this person to EDIT the application, including any supporting documents/stipulations requested during the review process:

No

1.7 Include this person on all correspondences regarding this application: (Note: This will include all committee correspondence, decision outcomes, renewal notices, and adverse event submissions.)

Yes

Credentials: Required for PI, Co-Is and Faculty Advisors

Upload or update your CV, resume, or biographical sketch.

Name	Version
Palmer_CV_9.18.18.pdf(0.01)	0.01

Conflict of Interest Detail: Required for all roles except Administrative Staff

Current Disclosure Status in M-Inform: *This study team member has indicated in M-inform that they do not have any outside interests to disclose.*

D1 Do you or your family members **have an** outside activity, relationship, or interest **with a** non-UM entity**, where the non-UM entity:**

- Provides financial or non-financial support for this project;
- Supplies a product used in this project (e.g., an app, device, compound, drug, software, survey, evaluation) either for free or at a cost (e.g., purchased);
- Holds an option or license to intellectual property used in this project (e.g., a device, compound, drug, software, survey, evaluation, code, data, schematics, algorithms) that you or your family member developed;
- Will perform work on this project (e.g., subcontract, service agreement, unfunded agreement); or
- Has a financial stake in the outcome of this research?

No

D2 If “Yes” to the question above, provide the name of the outside entity or entities and a brief description of the interest/relationship(s):

View: VIEW000072_customAttributes._attribute186_Study Team Detail
Section: 01. General Study Information

Study Team Detail

1.4 Team Member:

Katherine Scott-Andrews

Preferred email: katieqa@umich.edu
Business phone
Business address: School of Kinesiology School of Kinesiology Bldg #1000 48109-1048

1.5 Function with respect to project:

Research Staff

1.6 Allow this person to EDIT the application, including any supporting documents/stipulations requested during the review process:

No

1.7 Include this person on all correspondences regarding this application: (Note: This will include all committee correspondence, decision outcomes, renewal notices, and adverse event submissions.)

No

Credentials: Required for PI, Co-Is and Faculty Advisors

Upload or update your CV, resume, or biographical sketch.

Name	Version
Scott-Andrews CV(0.02)	0.02

Conflict of Interest Detail: Required for all roles except Administrative Staff

Current Disclosure Status in M-Inform: *This study team member has not yet disclosed in M-Inform.*

D1 Do you or your family members **have an** outside activity, relationship, or interest **with a** non-UM entity**, where the non-UM entity:**

- Provides financial or non-financial support for this project;
- Supplies a product used in this project (e.g., an app, device, compound, drug, software, survey, evaluation) either for free or at a cost (e.g., purchased);
- Holds an option or license to intellectual property used in this project (e.g., a device, compound, drug, software, survey, evaluation, code, data, schematics, algorithms) that you or your family member developed;
- Will perform work on this project (e.g., subcontract, service agreement, unfunded agreement); or
- Has a financial stake in the outcome of this research?

No

D2 If “Yes” to the question above, provide the name of the outside entity or entities and a brief description of the interest/relationship(s):

View: VIEW000072_customAttributes._attribute186_Study Team Detail
Section: 01. General Study Information

Study Team Detail

1.4 Team Member:

Matthew Stewart

Preferred email:	stewm@umich.edu
Business phone	734-764-1343
Business address:	School of Kinesiology School of Kinesiology Bldg #1000 48109-1048

1.5 Function with respect to project:

Research Staff

1.6 Allow this person to EDIT the application, including any supporting documents/stipulations requested during the review process:

No

1.7 Include this person on all correspondences regarding this application: (Note: This will include all committee correspondence, decision outcomes, renewal notices, and adverse event submissions.)

No

Credentials: Required for PI, Co-Is and Faculty Advisors

Upload or update your CV, resume, or biographical sketch.

Name	Version
Matt Stewart CV(0.01)	0.01

Conflict of Interest Detail: Required for all roles except Administrative Staff

Current Disclosure Status in M-Inform: *This study team member has indicated in M-inform that they do not have any outside interests to disclose.*

D1 Do you or your family members **have an** outside activity, relationship, or interest **with a** non-UM entity**, where the non-UM entity:**

- Provides financial or non-financial support for this project;
- Supplies a product used in this project (e.g., an app, device, compound, drug, software, survey, evaluation) either for free or at a cost (e.g., purchased);
- Holds an option or license to intellectual property used in this project (e.g., a device, compound, drug, software, survey, evaluation, code, data, schematics, algorithms) that you or your family member developed;
- Will perform work on this project (e.g., subcontract, service agreement, unfunded agreement); or
- Has a financial stake in the outcome of this research?

No

D2 If "Yes" to the question above, provide the name of the outside entity or entities and a brief description of the interest/relationship(s):

View: VIEW000072_customAttributes._attribute186_Study Team Detail
Section: 01. General Study Information

Study Team Detail

1.4 Team Member:

Carissa Wengrovius

Preferred email: wengrov@umich.edu
Business phone
Business address: 48109

1.5 Function with respect to project:

Research Staff

1.6 Allow this person to EDIT the application, including any supporting documents/stipulations requested during the review process:

No

1.7 Include this person on all correspondences regarding this application: (Note: This will include all committee correspondence, decision outcomes, renewal notices, and adverse event submissions.)

No

Credentials: Required for PI, Co-Is and Faculty Advisors

Upload or update your CV, resume, or biographical sketch.

Name	Version
C.Wengrovius_CV_5.20.docx(0.02)	0.02

Conflict of Interest Detail: Required for all roles except Administrative Staff

Current Disclosure Status in M-Inform: *This study team member has indicated in M-inform that they do not have any outside interests to disclose.*

D1 Do you or your family members **have an** outside activity, relationship, or interest **with a** non-UM entity**, where the non-UM entity:**

- Provides financial or non-financial support for this project;
- Supplies a product used in this project (e.g., an app, device, compound, drug, software, survey, evaluation) either for free or at a cost (e.g., purchased);
- Holds an option or license to intellectual property used in this project (e.g., a device, compound, drug, software, survey, evaluation, code, data, schematics, algorithms) that you or your family member developed;
- Will perform work on this project (e.g., subcontract, service agreement, unfunded agreement); or
- Has a financial stake in the outcome of this research?

No

D2 If "Yes" to the question above, provide the name of the outside entity or entities and a brief description of the interest/relationship(s):

View: VIEW000072_customAttributes._attribute186_Study Team Detail
Section: 01. General Study Information

Study Team Detail

1.4 Team Member:

Indica Sur

Preferred email: isur@umich.edu
Business phone
Business address: School of Kinesiology 1271C CCRB, 401 Washtenaw Avenue 48109-2013

1.5 Function with respect to project:

Administrative Staff

1.6 Allow this person to EDIT the application, including any supporting documents/stipulations requested during the review process:

Yes

1.7 Include this person on all correspondences regarding this application: (Note: This will include all committee correspondence, decision outcomes, renewal notices, and adverse event submissions.)

Yes

Credentials: Required for PI, Co-Is and Faculty Advisors

Upload or update your CV, resume, or biographical sketch.

Name	Version
There are no items to display	

Conflict of Interest Detail: Required for all roles except Administrative Staff

Current Disclosure Status in M-Inform: *This study team member has not yet disclosed in M-Inform.*

D1 Do you or your family members **have an** outside activity, relationship, or interest **with a** non-UM entity**, where the non-UM entity:**

- Provides financial or non-financial support for this project;
- Supplies a product used in this project (e.g., an app, device, compound, drug, software, survey, evaluation) either for free or at a cost (e.g., purchased);
- Holds an option or license to intellectual property used in this project (e.g., a device, compound, drug, software, survey, evaluation, code, data, schematics, algorithms) that you or your family member developed;
- Will perform work on this project (e.g., subcontract, service agreement, unfunded agreement); or
- Has a financial stake in the outcome of this research?

D2 If "Yes" to the question above, provide the name of the outside entity or entities and a brief description of the interest/relationship(s):

01-1. Application Type

1-1.1* Select the appropriate application type.

	Application Type	Description
☑	Human Subjects research **involving interaction or intervention** *(formerly Standard, non-exempt research project - or - Exempt)*	Studies that involve either or both of the following:<br>• Interaction, including communication or interpersonal contact between investigator and subject<br>• Intervention, including both physical procedures by which information or biospecimens are gathered (e.g., venipuncture) and manipulations of the subject or subject's environment that are performed for research purposes<br><br>Interaction/Intervention studies may also have a "secondary research" component.

Does the research involve any of the following:

a. more than minimal risk to participants?
b. use of drugs or medical devices?
c. target prisoners as research subjects?
d. collection of biospecimens from subjects (including blood, saliva, cheek swabs)?

● **Yes** ○ No

☐	**Secondary research** uses of private information or biospecimens	"Secondary research" are studies that involve ONLY re-using private information and/or biospecimens that are collected for some other "primary" or "initial" activity, such as other earlier research studies, a biorepository holding specimens obtained with "broad consent," clinical care, or educational records. Includes Exemption 4 and "not regulated" projects.<br><br>**Do NOT use this application type for:**<br>• Studies that **also** have an interaction/intervention component, such as primary collection of information or biospecimens for the purposes of the study. (Choose instead "Human subjects research involving **interaction or intervention**.")<br>• Projects involving secondary use of information/biospecimens for **only non-research purposes**, such as QA/QI, case studies on one or two individuals, or use in a class to teach research methods. (Choose instead "Activities **not regulated** as human subjects research.")
☐	Activities **Not Regulated** as human subjects research	Not all activities that involve people, their data, or specimens are covered by the regulations governing human subjects research (45 CFR 46 or 21 CFR 50/56).<br><br>**IRB review is required** for the following activities **ONLY** to assess compliance with **HIPAA** or other regulations or institutional policies:<br>• Research on existing data or specimens that have been coded before the researcher receives them, but identifiers still exist.<br>• Research Involving Deceased Individuals Only<br>• Pre-review of Clinical Data Sets Preparatory to Research<br>• Standard Public Health Surveillance or Prevention Activities<br><br>**IRB review is not required for the following activities**, but researchers may wish complete this brief application to generate a determination letter for funding or publication purposes, or to

		request IRB review to confirm the "Not Regulated" determination:<br>• Case Studies<br>• Class Activities<br>• Journalism/Documentary Activities<br>• Oral History<br>• Quality Assurance and Quality Improvement Activities<br>• Research on Organizations<br>• Research using Publicly Available Data Sets
☐	Projects **lacking immediate plans for involvement of human subjects**, their data, and/or their specimens	Activities such as training grants, program projects, center grants, or multi-phase studies not involving human subjects until later years. Before release of funding, some agencies may require IRB acknowledgement of the future use of human subjects.<br><br>These projects are sometimes referred to as "umbrella projects" or "dry applications."
☐	**Single-patient Expanded Access Drug or Biologic (Emergency Use or Non-Emergency/Compassionate Use)**	Use of an investigational drug or biologic, outside of a clinical trial, under a single-patient IND issued by the FDA for a patient faced with a serious or life-threatening disease or condition.<br>• Contact the IRB Chair-on-Call as soon as possible once the decision to use the investigational drug or biologic is made.<br>• Submission for IRB review and approval is required, prior to use if feasible. **If this was an emergency use, submit no later than five days after use of the investigational agent.**<br>• This includes both one-time use and continuing therapy.
☐	**Single-patient Expanded Access Device Use (Emergency Use or Non-Emergency/Compassionate Use)**	Use of an investigational device, outside of a clinical trial, when this is the only option available for a patient faced with a serious or life-threatening disease or condition.<br>• Contact the IRB Chair-on-Call as soon as possible once the decision to use the investigational device is made.<br>• Submission for IRB review and approval is required, prior to device use if feasible. **If this was an emergency use, submit no later than five days after use of the investigational device.**<br>• This includes both one-time use and continuing therapy.
☐	**Humanitarian Use Device** (HUD) under a HDE	Non-research, on-label use of an HUD under a Humanitarian Device Exemption (HDE)
☐	Requesting Review by a **Non-UM IRB**	Use ONLY to request deferral of IRB oversight for UM activities to a non-UM IRB or when UM is a performance site in a multisite research project where UM is the lead site.
☐	**Multi-site Research** where U-M is a Coordinating Center and/or IRB of Record	Do not use Multi-site Research application type when U-M is **only** a performance site - select Standard application type.<br><br>Select when U-M is any of the following:<br>• Data Coordinating Center;<br>• Clinical Coordinating Center; or<br>• IRB of Record for non-U-M sites (for U-M to be IRB of Record you must contact your IRB for prior acknowledgement).<br><br>When U-M is **also** a performance site, a separate application is required for local site considerations.<br>Refer to special requirements at the IRB website.

View: 01-2. Standard Study Information
Section: 01. General Study Information

01-2. Standard Study Information

1-2.1* Who initiated this study?

Investigator

1-2.2* Are you or any students working on this project being paid from a federally funded training grant?

○ Yes ● **No**

1-2.3 This study is currently associated with the following department. To associate this research with a different department, click Select. If the department has defaulted to "student", click select to specify the department through which this application is being submitted.

School of Kinesiology

1-2.5* Is the study related to cancer, cancer risk, or cancer care delivery?

○ Yes ● **No**

1-2.7* Has the scientific merit of this study already been peer reviewed (i.e., reviewed by one or more recognized authorities on the subject)?

● **Yes** ○ No

1-2.7.1* List the peer-review organization(s).

Peer Review Organization
External sponsor review process (e.g. study section)

1-2.8* Is this a clinical trial?

● **Yes** ○ No

1-2.8.1* Please select the trial phase

	Trial Phase	Description
○	Phase 0	Exploratory study to determine whether agent behaves in humans as pre-clinical testing indicated
○	Phase I	Evaluate safety, dose range, and identify side effects in healthy volunteers or in patients with the disease of interest
○	Phase I/II	Safety, dosage levels and efficacy of new treatment; device pilot study
○	Phase II	Evaluate safety and efficacy of selected dosage levels and duration of treatment
○	Phase II/III	Larger study to confirm safety and efficacy of selected dose(s) and duration of treatment
○	Phase III	Larger, controlled trial to confirm efficacy compared to a standard treatment regimen or to no treatment; device pivotal study
○	Phase IV	Post-market study to provide additional information on risks, benefits, and optimal use
●	**Other**	

If other, please specify:
Exploratory study to determine the effects of a movement program on health-related outcomes in healthy volunteers.

View: 02. Sponsor/Support Information
Section: 02. Sponsor/Support Information

02. Sponsor/Support Information

The following sections request details about the current or pending sponsorship/support of this study. Consider all of the choices below and complete the appropriate sections.
*** Note: At least one of the following sections must be answered. Multiple forms of funding or support must be added one at a time.**

2.1 Please select all Proposal Approval Forms (PAFs), Awards (AWDs), and/or Unfunded Agreements (UFAs) associated with this study.

☐ **Click here to indicate that a PAF(s) has not been initiated.**

Related PAFs:

ID	Title	PI	Direct Sponsor	Prime Sponsor	State	Has SUBKs?	Related Awards
16-PAF01826	A PATH (Promoting Activity and Trajectories of Health) for Children	Leah Robinson	Health and Human Services, Department of-National Institutes of Health		Awarded	yes	AWD003046 (9/1/2016 - 6/30/2022)

Related AWDs:

Award ID	Title	PI	Direct Sponsor	Prime Sponsor	State	Has SUBKs?	Project Period	Awarded PAFs
AWD003046	A PATH (Promoting Activity and Trajectories of Health) for Children	Leah Robinson	Health and Human Services, Department of-National Institutes of Health		Active	yes	9/1/2016 to 6/30/2022	17-PAF02394, 16-PAF01826

Related UFAs:

UFA ID	Title	PI	State	Category	Start Date	End Date

There are no items to display

Note: As of 6/10/2018, details of the External Sponsor/Support have been replaced by linking to submissions in the eResearch Proposal Management system.

Click here to view the historical external sponsor/support details.

2.2 Internal UM Sponsor(s)/Support: [Including department or PI discretionary funding]

Type	Department Sponsor	Support Type

There are no items to display

2.3 Check here if the proposed study does not require external or internal sponsorship or support:

☐

2.4* Is there any other financial or non-financial sponsorship or support not covered in the sections above?

○ Yes ● **No**

View: 03. UM Study Functions
Section: 03. Performance Sites

03. UM Study Functions

3.1* Indicate all functions that will be performed at University of Michigan locations.

Select all that apply:

Recruitment (including screening)
Interaction (e.g., information gathering, survey, interview, focus groups, etc.)
Intervention (e.g., use of drug or device, medical procedures, educational intervention, group intervention, social/psychological intervention etc.)
Observation of behavior (direct or indirect)
Primary or secondary analysis (data/specimen)
Storage (data and/or specimen): Responsible for the management, security and transfer of study data and/or specimens.

If other, please specify.

View: 03-1. Performance Sites
Section: 03. Performance Sites

03-1. Performance Sites

3-1.1* Performance Sites:

Location	Country	"Engaged" in the research?	Performance Site Type	Site Function
Augusta University	USA	yes		Analysis
University of Michigan	USA	yes		Intervention,Storage,Interaction,Analysis,Observation,Recruitment
University of South Carolina	USA	yes		Storage,Analysis
Ypsilanti Community Schools (Ford Early Learning Center, Perry Early Learning Center, and Beatty Early Learning Center)	USA	no		Intervention,Interaction,Observation,Recruitment

View: Performance Site Detail
Section: 03. Performance Sites

Performance Site Detail

3-1.2* Location or Institution:

Augusta University

3-1.3 Address:

City
State
Country* USA

3-1.4* Function of this location with respect to this study:

Select all that apply:

Primary or secondary analysis (data/specimen)

If other, please specify:

3-1.5* Will this site be "engaged" in the conduct of the research?

(●) **Yes** ( ) No

3-1.6 If known, provide the Federalwide Assurance (FWA) number for this location.

3-1.7 If applicable, indicate what organization, agency or government office has reviewed this research and provided its approval (e.g., IRB, ethics committee, school district office, prison official, nursing home administrator).

3-1.8 Upload any location site approval documentation here:

Name	Version
There are no items to display	

View: Performance Site Detail
Section: 03. Performance Sites

Performance Site Detail

3-1.2* Location or Institution:

University of Michigan

3-1.3 Address:

City
State
Country* USA

3-1.4* Function of this location with respect to this study:

Select all that apply:

Recruitment (including screening)
Interaction (e.g., information gathering, survey, interview, focus groups, etc.)
Intervention (e.g., use of drug or device, medical procedures, educational intervention, group intervention, social/psychological intervention etc.)
Observation of behavior (direct or indirect)
Primary or secondary analysis (data/specimen)
Storage (data and/or specimen): Responsible for the management, security and transfer of study data and/or specimens.

If other, please specify:

3-1.5* Will this site be "engaged" in the conduct of the research?

● **Yes** ○ No

3-1.6 If known, provide the Federalwide Assurance (FWA) number for this location.

FWA00004969

3-1.7 If applicable, indicate what organization, agency or government office has reviewed this research and provided its approval (e.g., IRB, ethics committee, school district office, prison official, nursing home administrator).

3-1.8 Upload any location site approval documentation here:

Name	Version
There are no items to display	

View: Performance Site Detail
Section: 03. Performance Sites

Performance Site Detail

3-1.2* Location or Institution:

University of South Carolina

3-1.3 Address:

City	Columbia
State	SC
Country*	USA

3-1.4* Function of this location with respect to this study:

Select all that apply:

Primary or secondary analysis (data/specimen)

Storage (data and/or specimen): Responsible for the management, security and transfer of study data and/or specimens.

If other, please specify:

3-1.5* Will this site be "engaged" in the conduct of the research?

● **Yes** ○ No

3-1.6 If known, provide the Federalwide Assurance (FWA) number for this location.

3-1.7 If applicable, indicate what organization, agency or government office has reviewed this research and provided its approval (e.g., IRB, ethics committee, school district office, prison official, nursing home administrator).

USC has ceded to UM oversight

3-1.8 Upload any location site approval documentation here:

Name	Version
There are no items to display	

View: Performance Site Detail
Section: 03. Performance Sites

Performance Site Detail

3-1.2* Location or Institution:

Ypsilanti Community Schools (Ford Early Learning Center, Perry Early Learning Center, and Beatty Early Learning Center)

3-1.3 Address:

City YPSILANTI
State MI
Country* USA

3-1.4* Function of this location with respect to this study:

Select all that apply:

Recruitment (including screening)

Interaction (e.g., information gathering, survey, interview, focus groups, etc.)

Intervention (e.g., use of drug or device, medical procedures, educational intervention, group intervention, social/psychological intervention etc.)

Observation of behavior (direct or indirect)

If other, please specify:

3-1.5* Will this site be "engaged" in the conduct of the research?

○ Yes ● **No**

3-1.6 If known, provide the Federalwide Assurance (FWA) number for this location.

3-1.7 If applicable, indicate what organization, agency or government office has reviewed this research and provided its approval (e.g., IRB, ethics committee, school district office, prison official, nursing home administrator).

UM HSBS, National Institutes of Health Study Section, YCS School System

3-1.8 Upload any location site approval documentation here:

Name	Version
SKonicaHSta18083112130.pdf(0.01)	0.01
UM ROBINSON IRB LETTER - YCS.pdf(0.01)	0.01

05. Research Design

5.1* Is there a stand-alone scientific protocol document and/or research plan associated with this application?

● **Yes** ○ No

5.1.1* Click ADD to attach the document(s) electronically.

Name	Version
8.2.17 IRB Research Plan PATH+SOBC.docx(0.01)	0.01
Amendment 9.16.19 IRB Research Plan PATH+SOBC.docx(0.01)	0.01

5.1.2* Indicate the section where each of the following are covered in the attached protocol:

**Objective**	Please see page 1-2
**Specific Aim/Hypothesis**	Please see page 1 -2
**Background Information**	Please see pages 2 - 6
**Methodology**	Please see pages 6 - 18
**Statistical Design**	Please see page 18 - 20

5.1.3* Study team Experience: Briefly outline the experience and competence of the study team to pursue the proposed study.

Please see page 6.

Additional staff and support for the project includes: Kara K. Palmer, MEd, and Katherine Andrews are PhD students in the School of Kinesiology, and Katherine Chinn, is a Research Technician. Indica Sur and Marcia Wallin provide administrative support. These individuals will assist with various aspects on this project that includes recruitment, consenting, data collection and data processing/entry. Additionally, Andrews and Palmer have training in motor skill development in pediatric populations. Palmer and Andrews will implement the CHAMP intervention.

Consultant Dr. Kip Webster (Augusta University) has expertise in motor skill analysis and will assist with coding the TGMD motor skill assessments. Dr. Cathy Antonakos (University of Michigan) is a research area specialist for the UM School of Kinesiology and will be assisting with physical activity data analysis.

Ame00084926
Matthew Stewart is currently a Kinesiology Ph.D. student whose research is focused on environment and policy. Matthew earned his Masters from Iowa State University where he worked in a lab that focused on accelerometry. He has specialized experience with ActiLife and Actigraph data analysis.

Ame00092961
Bryan Terlizzi is a PhD student in the University of South Carolina Department of Physical Education and will be assisting with data collection for the project. Bryan has a Master's of Science in Health, Exercise, and Sport Science from The Citadel (2017). He has served as part of a research team on an NIH funded study analyzing qualitative and quantitative movement data in underserved children, as well as several other short-term practical experiences with young children in the classroom setting.

Ame00094488
Dr. Kip Webster's affiliation was updated to Assistant Professor at Augusta University.

5.2* Will the involvement of ANY subjects in this study be limited to analysis of their existing data or specimens?

○ Yes ● **No**

5.3* Will the study involve recruitment and/or participation of subjects in order to produce new data (e.g., surveys, interaction, intervention)? *[Require sections 8-1 and 11-3]*

● **Yes** ○ No

5.4* List the inclusion and exclusion criteria for this study population and/or data set. (If covered in attached protocol, indicate section)

As noted in the Methods sections (pages 5 - 13)

Inclusion/Exclusion Criteria. Preschoolers who are ≥ 3.5 years are eligible to participate. Any preschooler with a specified developmental and/or physical disability (noted from school records) will receive the intervention is part of a CHAMP class, but data will not be used for research purposes.

5.5 Identify any racial, ethnic, or gender group(s) that will be specifically excluded from

participation in this research study and provide a compelling justification for such exclusion:

N/A; no racial, ethnic, or gender group(s) will be specifically excluded from participation in this research study.

5.6* Indicate the age range (in years) of the subject population in this study.

**Minimum Age:**	3.5
**Maximum Age:**	5If no upper limit, enter "999"

View: 06. Benefits and Risks
Section: 06. Benefits and Risks

06. Benefits and Risks

6.1 * Describe the potential benefits of this research to society.

There are no direct benefits to the child for participating in this study. However, motor skills are important to the overall growth and development of children. Previous evidence support that CHAMP is effective in promoting motor skill in preschool- and school-age children. These findings could help support and develop early childhood education programs.

6.2 * Will results of the research be communicated back to the subjects?

(●) **Yes** ( ) No

6.2.1 * Explain the plan and process.

Parents/guardian of participants will be given a report of children's findings as relates to the motor competence, physical activity, and perceived competence along with body composition measures. These findings will be mailed to parents via postal services once the data has been analyzed at the end of year one and then following the post assessments.

6.3 * Describe any direct risks to the public or community, which could result from this research?

N/A. There is little to no direct risk to the public or community that will result from the research. Children will be engaging planned movement and physical activity that in common in physical education programs. The program will be under the supervision of a research who possesses a PhD with over 10 years of research and teaching experience in these settings.

6.4 * Does this project involve study arms that have differing levels of benefit or risks to subjects?

( ) Yes (●) **No**

6.5 * Benefits and Risks:

Click "Add" to begin entering the benefit and risk level detail information associated with this study.

	Name	Risk Level	Direct Benefit
View	HUM00133319	No more than minimal risk	no

View: Benefits and Risks Detail
Section: 06. Benefits and Risks

Benefits and Risk Level Detail

If a study involves multiple arms or phases that pose different levels of risk or direct benefits to subjects, then create an entry for each arm or phase using the "OK and Add Another" option at the bottom of this page. Only one entry is necessary if the risk level and the direct benefit to subjects is the same for the entire project, even if the study involves multiple arms or phases.

6.5.1 * Name of Arm (experimental group, study wave, etc.)

HUM00133319

6.5.2 * Description of Arm (experimental group, study wave, etc.)

6.6 * Are there potential direct benefits of this research to the subjects?

○ Yes ● **No**

6.7 * Provide a description of the foreseeable risks to subjects. For studies involving multiple arms or phases, enter the risks for this arm or phase only.
Provide a description of the foreseeable risks to the subjects.
For EACH identified risk, include:

- **Likelihood of the risk,**
- **Seriousness to the subject; and**
- **What measures will be taken to minimize the risk (for example, study design includes the substitution of procedures already being performed on the subject for diagnostic or treatment purposes, or in a study of Post-Traumatic Stress Disorder, the investigator takes steps to identify, manage, or refer as appropriate, subjects for whom the study may evoke very difficult emotions)**

If possible, please use the following categories to assess the likelihood:

- **"Common" (i.e., approximate incidence > 25%)**
- **"Likely" (i.e., approximate incidence of 10-25%)**
- **"Infrequent" (i.e., approximate incidence of 1-10%)**
- **"Rare" (i.e., approximate incidence < 1%):**

For both CHAMP and control arms, risk in the study rare (i.e., infrequent; approximate incidence of 1-10%). This risk includes physical injury (e.g., falling down, being hit with a ball, etc) when engaging in movement and physical activity. A proper students: teacher ratio and student:space will be maintained to ensure the safety of the children.

The researchers have taken steps to minimize the risks of this study. In past studies, parents expressed concerned about the children wearing the physical activity monitors on the ankle during winter – it was difficult as it relates to getting dressed/undressed (e.g., as it relates to wearing tights and boots). For data collection, children will wear the physical activity monitors on their wrist like a watch. There is a risk that participants could lose or break the actigraph devices. If this occurs, the participants or parent/guardian will not be held financial liable for the device, but attempts will be made to find the device if it is lost. Participants will not be given a second device for lost or non-returned devices. The has been approved in a previous protocol under the PI at UoM.

Another risk of the study is breach of confidentiality as it relates to the video data that is required to code motor skill competence. This data will be stored on a secured computer and only coded by researchers and permanently deleted after coding is complete. Although the imagine of a child face might be captured, the focus of the video is on the mechanics of the body (i.e., movement of the arms, legs, and trunk).

6.8 * What is the level of risk of harm to the subjects, resulting from this arm of the research? For studies involving multiple arms or phases, enter the level of risk for this arm or phase only.

	Risk Level	Description
●	No more than minimal risk	**A risk is minimal where the probability and magnitude of harm or discomfort anticipated in the proposed research are not greater, in and of themselves, than those ordinarily encountered in daily life or during the performance of routine physical or psychological examinations or tests. For example, the risk of drawing a small amount of blood from a healthy individual for research purposes is no greater than the risk of doing so as part of a routine physical examination. (Note: The definition of minimal risk for research involving prisoners differs somewhat from that given for non-institutionalized adults.) Refer to the Risk Grid for more information.**
○	Minor increase over minimal risk	While this risk category may be used to classify research involving adult subject populations, it must be considered in the evaluation of risk in research involving children as defined in 45 CFR 46 sections 404-407*** Risks are more severe than those defined as “No more than minimal risk” and less severe than those described as “Moderate” on the Risk Grid.
○	Moderate risk	Refer to the Risk Grid for more information.

Risk Level	Description
◯ High risk	Requires scrutiny in regards to the likelihood of direct benefits, and whether or not benefits clearly outweigh risks. Refer to the Risk Grid for more information.

6.9 * Discuss why the risks to the subjects are reasonable in relation to the anticipated benefits.

The risk noted above of no more than minimal risk. The researchers will take all steps to to minimize the risks to participants in this study. It is not unusual for children to experience falls, bumps, and bruises in movement and physical activity settings. But the benefits of participation in physical play outweigh the risks.

Understanding physical activity behaviors in young children are critical. Steps have been made to make wearing the actigraph monitors devices more favorable by both the parents and child. For data collection, children will wear the physical activity monitors on their wrist like a watch.

07. Special Considerations

7.1* Does this study involve human tissue or biological specimens (use, collection, or secondary analysis) (e.g. blood, urine, bone marrow, skin, etc.)? [Require Section 18]

○ Yes ● **No**

7.2* Does this study involve the secondary analysis **of a** pre-existing data set**, including data associated with any specimens identified in response to question 7.1? [Require Section 24]**

○ Yes ● **No**

7.3* Will the research involve the access, collection, use, maintenance, or disclosure of protected health information (PHI)? PHI is:

- **information about a subject's past, present, or future physical or mental health, the provision of healthcare to a subject, or payment for the provision of healthcare to a subject; AND**
- **maintained by a HIPAA-covered entity (e.g. healthcare provider, healthcare plan, or healthcare clearinghouse).**

[Require Section 25]

○ Yes ● **No**

View: 07-1. Special Considerations - Continued
Section: 07. Special Considerations

07-1. Special Considerations - Continued

7-1.1* Will subjects receive payment or other incentives for their participation in the study? [Require Section 13]

(●) **Yes** ( ) No

7-1.2* Will subjects undergo healthcare-related treatments or procedures (standard of care and/or research) as part of the study? [Require Section 14]

( ) Yes (●) **No**

7-1.3* Does this study involve the deception **or concealment of subjects? [Require Section 27]**

( ) Yes (●) **No**

7-1.4* Excluding routine email correspondence, does this study involve the use of the Internet or email as an integral part of the research design or will sensitive information be transmitted by e-mail? [Require Section 28]

( ) Yes (●) **No**

7-1.5* Will the study collect data using surveys, interviews, or focus groups? [Require Section 29]

(●) **Yes** ( ) No

7-1.6* Does this study require subjects to listen to an audio recording or view images? [Require Section 31]

(●) **Yes** ( ) No

7-1.7* Will any drugs, biologics, radiopharmaceuticals, nutritional (e.g., herbal or alternative medication) supplements or other material be administered, implanted, or applied to the subjects as the object of the study? [Require Section 15]

( ) Yes (●) **No**

7-1.8* Will the study involve a placebo (drug, device, procedure, intervention, surgery, etc.) control group? [Require Section 17]

(●) **Yes** ( ) No

7-1.8.1* Is the placebo for a drug? [Require Section 15]

( ) Yes (●) **No**

7-1.9* Will the study involve human embryonic stem cells (hESCs) or induced pluripotent stem cells? [Require Section 19]

( ) Yes (●) **No**

7-1.10* Will the study have a Data and Safety Monitoring Plan (DSMP)? [Require Section 32]

( ) Yes (●) **No**

View: 07-2. Special Consideration - Continued
Section: 07. Special Considerations

7-2. Special Consideration - Continued

**7-2.1* Will any devices be used, administered, implanted, or applied to the subjects, or will human specimens be used to test in vitro diagnostic devices?
[IRBMED Applications Require Section 16]**

● **Yes** ○ No

7-2.1.1* Describe all devices that are the OBJECT of the study, or ARE RELEVANT to the study. If this study is designed to test the safety or efficacy of any of these devices, then this project is FDA-regulated and must be reviewed by IRBMED.

This study measures
1. physical activity through the use of actigraph
2. use tablets to measures two other measures perceived motor competence and self-regulations.
All of the measures are relevant to the study and approved research tools / measures. Please note that these measures have been approved in previous IRB protocols and are published in research studies. This study is not designed to test the safety or efficacy of any of these devices.

7-2.2* Is the research testing or utilizing a health-related mobile software application that is:

- Designed for a handheld (e.g., smartphone) or wearable mobile device (e.g., exercise tracking), or
- Tailored to a mobile platform (i.e., a handheld commercial or off-the-shelf computing platform, with or without wireless connectivity) but executed (run) from a server

and the mobile software application/platform performs any of the following:

- Uses a built-in feature of a device such as light, vibration, or camera to perform a medical device function.
- Connects or links to an existing device to control its operation, function, or energy source.
- Uses patient-specific data from a connected device including a sensor or electrode to monitor, manipulate, calculate, or analyze information.
- Conveys diagnostic information, or provides education materials or encouragement.
- Performs calculations, conversions, measurements or interpretations.

○ Yes ● **No**

**7-2.3* Will the subjects be exposed to any ionizing radiation during the course of this study?
[Require Section 21]**

○ Yes ● **No**

7-2.4* Will any organs, tissues, or cells from humans (including fetal tissue) or animals be administered to the subjects for the purposes of this study? [Require Section 22]

○ Yes ● **No**

7-2.5* Does this study involve a gene transfer intervention or an intervention based on recombinant DNA technology? [Require Section 23]

○ Yes ● **No**

View: 08. Subject Participation
Section: 08. Subject Detail

08. Subject Participation

8.1* Please indicate the number of subjects to be enrolled from ALL study locations to achieve the goal of the study:

300

8.2* Enter the estimated number of subjects to be enrolled at each University of Michigan site:

Location or Institution	Total
**Ypsilanti Community Schools (Ford Early Learning Center, Perry Early Learning Center, and Beatty Early Learning Center)**	
Adults	0
Children	300
**University of Michigan**	
Adults	0
Children	0
**Augusta University**	
Adults	
Children	
**University of South Carolina**	
Adults	0
Children	0
**Total from all University of Michigan sites:**	**300**

View: 08-1. Subject Recruitment
Section: 08. Subject Detail

08-1. Subject Recruitment

8-1.1* At what point in the study are you planning on beginning the recruitment of subjects?

0-2 years after approval

8-1.2* Indicate which of the following established subject pools, if any, will be used for recruitment.

Select all that apply:

Children at their preschool, elementary, middle, or high school location

Provide Related UM IRB Project Number or Subject Pool Description:

8-1.3* Describe the manner in which potential study subjects will be recruited. List how, when, who will recruit and where they will be recruited. Include any provisions to protect or maintain subject privacy.

Upon receiving IRB approval, the consenting process will occur through multiple methods (see below Informed Consenting Methods for specific details). Parent/guardian will be given a one-time $5.00 cash incentive once the IRB is returned. Additionally, fully completed Family Questionnaires will be entered into a drawing to win one (1) of eight (8) $50.00 Gift Cards to Wal-Mart.

Recruiting and Informed Consenting Methods

Parents/guardians who do not consent to participate in the project after the mass distribution and/or Welcome Night methods will be contacted through the following methods. Then no more attempts will be made.

1. Mass Distribution. Each parent/guardian will receive an informational letter from the preschool/early learning center principal supporting the project and notifying the parents they are not obligated to participate in the study. This will contain the informed consent letter from the PI regarding the project.
a. Letters will be mailed through USPS before the first day of the academic year with a self-addressed returned enveloped.

2. Welcome Night. The PI and member of the investigative team will present an overview of the program, go over the assessment, and IRB Consent Form with the parents on the Welcome Night (2 days before the 1st day of School). The team with also have some fun games, and movement activities set up for Family Fun Night.

Before the start of school,
3a. Home visits. The Family Support Staff Personnel will contact families and request for a research staff that has completed the PEERRS and GCP training (PI-Robinson, Research Staff – Palmer and/or Chinn) to attend the required Home Visit with the Classroom Teacher or Center Family Support Staff Personnel that is Sept 5th. The Research Staff will explain the study 1:1 with the parent/guardian, go over the inform consent letter, answer any questions, and assist the parent/guardian with the Family Questionnaire.

3b. Preschool Registration/Sign Up Day. The PI and members of the investigative team will be present at the Registration/Sign-Up Day for the parent to address any questions and to discuss the project. Informed consents and Family Questionnaire packets will be available to parents.

Once school begins (To children who have not consented)
4a. Classroom teachers distribute letters. Letters/informed consent packets will be distributed by classroom teachers in a sealed enveloped on the and returned in a sealed envelope.

4b. Drop-off and Pick-up times. The PI and/or members of the investigative team will be present give an overview of the study and go over the IRB Consent Form during drop-off and pick-up times in the morning and afternoon at the center.

Parents who agree to participate in the project will receive a copy of the consent form for their personal records. Parents will receive a copy of their child's results an overview of the study's findings and how their child's participation contributed to advancing research that focuses on health-related concerns in pediatric populations. A parent and/or child has the right to withdraw from the study at any time. Participation is voluntary and will in no way influence their relationship with the preschool centers, School of Kinesiology, nor the University of Michigan. All children who have received parental consent to participate in the study must also provide verbal assent. The script for the verbal assent is:

"Good Morning (Student Name). Your Mommy/Daddy told me it is okay for you to be in this project. We will play together during school in some games. I will also see how big you are, how well you move, and show you some pictures/videos and ask you to tell me "which one moves most like you." We will see how much you play by wearing this small device on your wrist (show the child the device). We will also play some games with either other and on a computer screen.

Do you want to play with me? Yes or No.

No, parental re-consent, only child re-assent. Only, will re-consent with parents if changes are made to the study.

Children are re-assessed during grades K, 1, and 2 following the intervention to examine the long-term effects of the CHAMP program. The re-consenting process will not occur with parents but they will receive reminder and a letter about upcoming assessments for the PATH study. Parents will also receive their child's assessment reports.
We will re-assent children to participant in follow-up assessment (attached a new re-assent script). Since this is a 2-year cohort study. We will be in each site on a regular basis and quite familiar with the families and children. Re-consent with parents will occur there are changes made to the study.

8-1.3.1 If applicable, how will prospective subjects' healthcare providers (e.g., physician, dentist, etc.) be involved in the recruitment and/or be notified of their individual patients' participation in the study?

N.A

8-1.4* Explain how the recruitment strategy is equitable and represents the population required for the study. If the information is covered in the attached protocol, please indicate section.

Upon receiving IRB approval, the consenting process will occur through multiple methods (see below Informed Consenting Methods for specific details). Parent/guardian will be given a one-time $5.00 cash incentive once the IRB is returned. Additionally, fully completed Family Questionnaires will be entered into a drawing to win one (1) of eight (8) $50.00 Gift Cards to Wal-Mart.

Recruiting and Informed Consenting Methods

Parents/guardians who do not consent to participate in the project after the mass distribution and/or Welcome Night methods will be contacted through the following methods. Then no more attempts will be made.

1. Mass Distribution. Each parent/guardian will receive an informational letter from the preschool/early learning center principal supporting the project and notifying the parents they are not obligated to participate in the study. This will contain the informed consent letter from the PI regarding the project.
a. Letters will be mailed through USPS before the first day of the academic year with a self-addressed returned enveloped.

2. Welcome Night. The PI and member of the investigative team will present an overview of the program, go over the assessment, and IRB Consent Form with the parents on the Welcome Night (2 days before the 1st day of School). The team with also have some fun games, and movement activities set up for Family Fun Night.

Before the start of school,
3a. Home visits. The Family Support Staff Personnel will contact families and request for a research staff that has completed the PEERRS and GCP training (PI-Robinson, Research Staff – Palmer and/or Chinn) to attend the required Home Visit with the Classroom Teacher or Center Family Support Staff Personnel that is Sept 5th. The Research Staff will explain the study 1:1 with the parent/guardian, go over the inform consent letter, answer any questions, and assist the parent/guardian with the Family Questionnaire.

3b. Preschool Registration/Sign Up Day. The PI and members of the investigative team will be present at the Registration/Sign-Up Day for the parent to address any questions and to discuss the project. Informed consents and Family Questionnaire packets will be available to parents.

Once school begins (To children who have not consented)
4a. Classroom teachers distribute letters. Letters/informed consent packets will be distributed by classroom teachers in a sealed enveloped on the and returned in a sealed envelope.

4b. Drop-off and Pick-up times. The PI and/or members of the investigative team will be present give an overview of the study and go over the IRB Consent Form during drop-off and pick-up times in the morning and afternoon at the center.

Parents who agree to participate in the project will receive a copy of the consent form for their personal records. Parents will receive a copy of their child’s results an overview of the study’s findings and how their child’s participation contributed to advancing research that focuses on health-related concerns in pediatric populations. A parent and/or child has the right to withdraw from the study at any time. Participation is voluntary and will in no way influence their relationship with the preschool centers, School of Kinesiology, nor the University of Michigan. All children who have received parental consent to participate in the study must also provide verbal assent. The script for the verbal assent is:

“Good Morning (Student Name). Your Mommy/Daddy told me it is okay for you to be in this project. We will play together during school in some games. I will also see how big you are, how well you move, and show you some pictures/videos and ask you to tell me “which one moves most like you.” We will see how much you play by wearing this small device on your wrist (show the child the device). We will also play some games with either other and on a computer screen.

Do you want to play with me? Yes or No.

No, parental re-consent, only child re-assent. Only, will re-consent with parents if changes are made to the study.

Children are re-assessed during grades K, 1, and 2 following the intervention to examine the long-term effects of the CHAMP program. The re-consenting process will not occur with parents but they will receive reminder and a letter about upcoming assessments for the PATH study. Parents will also receive their child’s assessment reports.
We will re-assent children to participant in follow-up assessment (attached a new re-assent script). Since this is a 2-year cohort study. We will be in each site on a regular basis and quite familiar with the families and children. Re-consent with parents will occur there are changes made to the study.

8-1.5* Does the recruitment strategy involve contacting individuals multiple times in an effort to secure their initial enrollment into the study?

● **Yes** ○ No

8-1.5.1* Describe how frequently and in what manner individuals will be contacted. If the information is covered in the attached protocol, please indicate section.

Upon receiving IRB approval, the consenting process will occur through multiple methods (see below Informed Consenting Methods for specific details). Parent/guardian will be given a one-time $5.00 cash incentive once the IRB is returned. Additionally, fully completed Family Questionnaires will be entered into a drawing to win one (1) of eight (8) $50.00 Gift Cards to Wal-Mart.

Recruiting and Informed Consenting Methods

Parents/guardians who do not consent to participate in the project after the mass distribution and/or

Welcome Night methods will be contacted through the following methods. Then no more attempts will be made.

1. Mass Distribution. Each parent/guardian will receive an informational letter from the preschool/early learning center principal supporting the project and notifying the parents they are not obligated to participate in the study. This will contain the informed consent letter from the PI regarding the project.
a. Letters will be mailed through USPS before the first day of the academic year with a self-addressed returned enveloped.

2. Welcome Night. The PI and member of the investigative team will present an overview of the program, go over the assessment, and IRB Consent Form with the parents on the Welcome Night (2 days before the 1st day of School). The team with also have some fun games, and movement activities set up for Family Fun Night.

Before the start of school,
3a. Home visits. The Family Support Staff Personnel will contact families and request for a research staff that has completed the PEERRS and GCP training (PI-Robinson, Research Staff – Palmer and/or Chinn) to attend the required Home Visit with the Classroom Teacher or Center Family Support Staff Personnel that is Sept 5th. The Research Staff will explain the study 1:1 with the parent/guardian, go over the inform consent letter, answer any questions, and assist the parent/guardian with the Family Questionnaire.

3b. Preschool Registration/Sign Up Day. The PI and members of the investigative team will be present at the Registration/Sign-Up Day for the parent to address any questions and to discuss the project. Informed consents and Family Questionnaire packets will be available to parents.

Once school begins (To children who have not consented)
4a. Classroom teachers distribute letters. Letters/informed consent packets will be distributed by classroom teachers in a sealed enveloped on the and returned in a sealed envelope.

4b. Drop-off and Pick-up times. The PI and/or members of the investigative team will be present give an overview of the study and go over the IRB Consent Form during drop-off and pick-up times in the morning and afternoon at the center.

Parents who agree to participate in the project will receive a copy of the consent form for their personal records. Parents will receive a copy of their child's results an overview of the study's findings and how their child's participation contributed to advancing research that focuses on health-related concerns in pediatric populations. A parent and/or child has the right to withdraw from the study at any time. Participation is voluntary and will in no way influence their relationship with the preschool centers, School of Kinesiology, nor the University of Michigan. All children who have received parental consent to participate in the study must also provide verbal assent. The script for the verbal assent is:

"Good Morning (Student Name). Your Mommy/Daddy told me it is okay for you to be in this project. We will play together during school in some games. I will also see how big you are, how well you move, and show you some pictures/videos and ask you to tell me "which one moves most like you." We will see how much you play by wearing this small device on your wrist (show the child the device). We will also play some games with either other and on a computer screen.

Do you want to play with me? Yes or No.

No, parental re-consent, only child re-assent. Only, will re-consent with parents if changes are made to the study.

Children are re-assessed during grades K, 1, and 2 following the intervention to examine the long-term effects of the CHAMP program. The re-consenting process will not occur with parents but they will receive reminder and a letter about upcoming assessments for the PATH study. Parents will also receive their child's assessment reports.
We will re-assent children to participant in follow-up assessment (attached a new re-assent script). Since this is a 2-year cohort study. We will be in each site on a regular basis and quite familiar with the families and children. Re-consent with parents will occur there are changes made to the study.

8-1.6* Indicate which methods will be used for recruitment?

Check all that apply:

Face-to-face contact (e.g. during a health care visit or an interview at a home address, etc.)

Postal mail

Letter/flyer distributed through intermediary organization (e.g. schools, churches, clubs, etc.)

Other

If other please specify:

Face-to-face interactions at preschool events/site, letters/flyers distributed through preschool

8-1.7 How will any email, address, and/or telephone lists be obtained?

During recruitment, in informed consent document.

8-1.8* What materials will be used for recruitment? The IRB must approve all recruitment materials.

See Help for important information regarding the requirements for recruitment materials

Check all that apply:

Flyers

Oral scripts

Check all that apply:

Letters/postal mail

If other please specify:

These will be obtained indirectly from the school. We will complete the mailing (i.e., stuffing of the envelopes and application of labels) and pay for the postal services. The mailing will come directly from the school until the parent provides us that information in the consent process.

If Web pages will be used, provide the Web address (URL) for the location where the pages will be posted (also upload the content of the pages below):

Upload recruitment materials here:

See Help for more information about working with documents (e.g. uploading, downloading, and editing).

Name	Version
7.25.18_PATH_Child_RE-Assent_Script_CLEANED.docx(0.01)	0.01
7.25.18_PATH_FORD_Letter_to_Parents_CLEANED.docx(0.01)	0.01
7.25.18_PATH_PERRY_Letter_to_Parents_CLEANED.docx(0.01)	0.01
7.30.18_SCRIPT_CLEANED_(1).docx(0.01)	0.01
9.6.18_PATH_BEATTY_Letter_to_Parents_CLEANED.docx(0.01)	0.01
parent-reminder_year 1_NO UNDERLINE.jpeg(0.01)	0.01
parent-reminder_year 2_NO UNDERLINE.jpeg(0.01)	0.01

☐ **Check here if any of the materials are not available electronically.**

Note: Study Teams are encouraged to scan and upload documents. See Help for a list of sites with scanning facilities

View: 09. Survey Populations
Section: 09. Subject Populations

09. Survey Populations

9.1* Is the study limited to a survey of either:

- The general adult population (aged 18 or older); or
- A subgroup of the general population which does not specifically target:

- Pregnant women and/or fetuses
- Lactating women
- Women of child-bearing potential
- Prisoners
- Cognitively impaired adults
- College students
- Economically or educationally disadvantaged persons
- Patients of the study team
- Employees, students or trainees of the study team
- Family members of the study team

where the survey is the sole interaction with the subject and does not pose more than minimal risk?

○ Yes ● **No**

View: 09-1. Subject Populations
Section: 09. Subject Populations

09-1. Subject Populations

9-1.1* Is the research designed to include or allow the following populations?

Select all that apply

- [x] **Normal, healthy subjects**
- [ ] **Adults** *age 18 and older*
- [ ] **Minors able to consent** *to treatments or procedures involved in the research, under the applicable law of the jurisdiction in which the research will be conducted (e.g. emancipated minors or minors seeking treatment for certain conditions.)*
- [x] **Children and/or Viable Neonates** *(i.e. persons who have not yet reached the legal age for consent to treatments or procedures involved in the research, under the applicable law of the jurisdiction in which the research will be conducted) [Require Sections 33 and 41]*
- [ ] **Neonates of uncertain viability and/or nonviable neonates** (do not check this box if the research is solely retrospective. For retrospective research regarding neonates of uncertain viability, check the box for 'Children'. See Help for additional information.) [Require Section 34]
- [ ] **Individuals and/or products involving human in vitro fertilization**
- [ ] **Pregnant women and/or fetuses** [Require Sections 35 and 41]
- [ ] **Lactating women** [Require Section 36]
- [ ] **Women of child-bearing potential** [Require Section 37]
- [ ] **Prisoners** (If the research includes a study population that is likely to become incarcerated during the conduct of the research, also select this category) [Require Section 38 and 41]
- [ ] **Cognitively impaired adults** [Require Sections 39 and 41]
- [ ] **College students** [Require Sections 40 and 41]
- [ ] **Economically or educationally disadvantaged persons** [Require Section 41]
- [ ] **Patients of the study team** [Require Section 41]
- [ ] **Employees, students or trainees of the study team** [Require Section 41]
- [ ] **Family members of the study team** [Require Section 41]
- [ ] **Unknown, unspecified population**

View: 10. Informed Assent - Children
Section: 10. Informed Consent

10. Informed Assent - Children

10.2* What types of informed assent for children and parental consent/permission will be obtained?
NOTE "Parent" or "Parental" below refers to parent or guardian. See Help **for important instructions on selecting the appropriate category or categories.**

Children
With signature:

- [ ] Written document

Without signature (waiver of documentation):

- [ ] Written document
- [ ] Oral assent script

Waiver of assent:

- [ ] Request for waiver of informed assent

Other:

- [ ] Pre-existing assent covers this activity

Parents
With signature:

- [x] **Comprehensive written**

Without signature (waiver of documentation):

- [ ] Comprehensive written
- [ ] Comprehensive oral consent/permission script

Waiver of parental consent/permission:

- [ ] Request for waiver of parental consent/permission (Note: no longer required for screening/recruitment)

Other:

- [ ] Short form permission, comprehensive oral script, and witness
- [ ] Request to use substitute mechanism for parental permission where research is designed for conditions or for a subject population for which parental or guardian permission is not a reasonable requirement to protect the subjects
- [ ] Request for IRB to appoint an advocate for children who are wards of the state or any other agency, institution or entity – required for studies related to the children's status as wards that are approved under 46.406 or 46.407 (see section 33)
- [ ] Pre-existing consent/permission covers this activity

10.2.2* Describe the process to seek and obtain informed assent for children and parental consent/permission (e.g., setting, timing, personnel involved, arrangements for answering subject questions before and after the consent is signed).

Study enrollment will begin after receiving human subjects approval from the Institutional Review Board for Research Involving Human Subjects at the PI's University (University of Michigan) and obtaining parental consent/child assent. All children enrolled in the 2 preschools who meet the inclusion/exclusion criteria (e.g., Inclusion/Exclusion Criteria. Preschoolers who are ≥ 3.5 years are eligible to participate. Any preschooler with a specified developmental and/or physical disability (noted from school records) will receive the intervention if part of a CHAMP class, but data will not be used for research purposes) will have the opportunity to participate in this study.

INFORMED CONSENT AND/OR ASSENT.
All students enrolled in the Head Start Centers will have the opportunity to participate in this research study. Upon receiving IRB approval (see page 9 – 10 of the protocol):

Informed Consenting Methods
Upon receiving IRB approval, the consenting process will occur through multiple methods (see below Informed Consenting Methods for specific details). Parent/guardian will be given a one-time $5.00 cash incentive once the IRB is returned. Additionally, fully completed Family Questionnaires will be entered into a drawing to win one (1) of eight (8) $50.00 Gift Cards to Wal-Mart.

Recruiting and Informed Consenting Methods

Parents/guardians who do not consent to participate in the project after the mass distribution and/or Welcome Night methods will be contacted through the following methods. Then no more attempts will be made.

1. Mass Distribution. Each parent/guardian will receive an informational letter from the preschool/early learning center principal supporting the project and notifying the parents they are not obligated to participate in the study. This will contain the informed consent letter from the PI regarding the project.
a. Letters will be mailed through USPS before the first day of the academic year with a self-addressed returned enveloped.

2. Welcome Night. The PI and member of the investigative team will present an overview of the program, go over the assessment, and IRB Consent Form with the parents on the Welcome Night (2 days before the 1st day of School). The team with also have some fun games, and movement activities set up for Family Fun Night.

Before the start of school,
3a. Home visits. The Family Support Staff Personnel will contact families and request for a research staff that has completed the PEERRS and GCP training (PI-Robinson, Research Staff – Palmer and/or Chinn) to attend the required Home Visit with the Classroom Teacher or Center Family Support Staff Personnel that is Sept 5th. The Research Staff will explain the study 1:1 with the parent/guardian, go over the inform consent letter, answer any questions, and assist the parent/guardian with the Family Questionnaire.

3b. Preschool Registration/Sign Up Day. The PI and members of the investigative team will be present at the Registration/Sign-Up Day for the parent to address any questions and to discuss the project. Informed consents and Family Questionnaire packets will be available to parents.

Once school begins (To children who have not consented)
4a. Classroom teachers distribute letters. Letters/informed consent packets will be distributed by classroom teachers in a sealed enveloped on the and returned in a sealed envelope.

4b. Drop-off and Pick-up times. The PI and/or members of the investigative team will be present give an overview of the study and go over the IRB Consent Form during drop-off and pick-up times in the morning and afternoon at the center.

Once the signed informed consent is obtained, the entire informed consent document, including the executed signature page, will be photocopied. The copy will be sealed in an envelope and sent home with the students in their weekly folder materials, to go home to their parents. Parents will keep this copy for their records. The original document will be retained by the study team on university-secured password protected computers and kept in a locked file cabinet located in a key code access laboratory (Child Movement, Activity, and Developmental Health Lab) in Central Campus Recreation Building 1271C.

10.2.3* What criteria will be used to determine whether or not a child's assent to participate will be obtained, whether that assent will be oral or written, and whether documentation of the child's assent (e.g., signature on the assent form) will be obtained? If documentation of child's assent is to be waived, provide a justification.

Yes, due to the age of the participants we are requesting a waiver of documentation as it relates to child's assent. The researcher will record in the documents a yes or no as it relates to the child's assent.

Child Assent Script
"Good Morning/Afternoon (Student Name). How are you today? Your Mom or Dad said it is ok for you to be in the study but you also have to yes (ok) to participate. For this project, we will measure how much you move and how much you play by wearing that small red device (researcher will show the child the device). It shows us how much your move and play. We will play together with balls, bats, and do a lot of other things like run, jump, and skip so I can see how you move. Finally, I will show you some pictures and you will tell me which picture moves most like you."

Do you want to do these tasks and be in this study? If so, say Yes. If not, say No.

10.2.4* Are any of the following changes expected in the status of child subjects during the study?

Check all that apply:

Significant increase in cognitive capacity (i.e. gets older or regains consciousness)

10.2.4.1 If applicable, describe the plan to re-assent or obtain consent from the subject if any of the changes occur.

No, parental reconsent, only child reassent. Only, will re-consent with parents if changes are made to the study.

View: 10-1. Informed Consent
Section: 10. Informed Consent

10-1. Informed Consent

10-1.1* All documents related to consent, assent, permission, and or debriefing documents, including oral scripts must be uploaded here. If you are requesting a waiver of documentation of informed consent, upload a copy of any written materials to be provided to participants, and provide a written description of any information to be provided orally.

Name	Version
7.25.18_COHORT_1_PATH_SOBC_R01_ICD_CLEANED.docx(0.02)	0.02
7.25.18_PATH_Child_Assent_Script_CLEANED.docx(0.01)	0.01
7.25.18_PATH_Child_RE-Assent_Script_CLEANED.docx(0.01)	0.01
7.30.18_SCRIPT_CLEANED_(1).docx(0.01)	0.01
9.6.18_COHORT 2 PATH R01 Informed Consent_CLEANED.docx(0.03)	0.03
CLEAN 8.7.2017 PATH + SOBC - R01 Informed Consent.docx(0.01)	0.01

10-1.2* Will the subjects be audiotaped, videotaped, or photographed (identifiable images of subject) during the research?

● **Yes** ○ No

10-1.3* Is there a substantial likelihood that the research will be conducted among a non-English-speaking population?

○ Yes ● **No**

10-1.4* Indicate which anticipated costs could be the full or partial responsibility of the subject.

Check all that apply:

No anticipated costs

If other, please specify:

10-1.5* Is the study designed to collect identifiable information from primary research subjects about other individuals, including family members?

○ Yes ● **No**

10-1.6* At the conclusion of this study, will specimens and/or data be retained for future research use?

● **Yes** ○ No

10-1.7* Does the informed consent document explicitly notify subjects that their data and/or specimens will be stored for future research?

● **Yes** ○ No

10-1.8* Are subjects required to agree to retention of their data and/or specimens as a condition of participating in the research?

● **Yes** ○ No

10-1.8.1* Provide a justification for this requirement. If the information is included in the attached protocol, please indicate section.

This study examines the sustained effect of the intervention of time. Therefore, there is a need to retain the data to examine these changes over time.

View: 11. Confidentiality/Security/Privacy
Section: 11. Confidentiality, Security and Privacy

11. Confidentiality/Security/Privacy

11.1* Will the study team access any data that is linked to a subject's identity by name or other identifier or code? [Require Section 11-1]

● **Yes** ○ No

11.2* Explain how the subjects' privacy will be protected.

All measures and assenting process will be obtained and kept private. All measures will be assessed on an individual basis except for motor skill competence and body composition measurements (height, waist, and weight measures) will take place in the large open space. Body composition measures will be collected in small groups. When completing the body composition measures all children will be fully clothed only extra clothing (i.e., heavy coats) and shoes will be removed. If any child expresses any concern in taking any body composition assessment in front of a peer he/she will be allow to take that measurement privately. Actual measurements (i.e., numbers) will be kept private since these are young children the measurement number will have little meaning/value to the child. The measurement will be recorded and will not be publicized to other participants. Measurements will be logged directly on the data record sheet and not directly conveyed to the preschooler. Later, specific information will be given to the parent through the report.

11.3* How will the study team protect research records, data, and/or specimens against inappropriate use or disclosure, or malicious or accidental loss or destruction in order to protect the confidentiality of subject data?

Select all that apply:
Locked office
Locked cabinet or storage unit
Restricted access
Restrictions on copying study-related materials
Access rights terminated when authorized users leave the project or unit
Secure laptop
Individual ID plus password protection
Security software (firewall, anti-virus, anti-intrusion) is installed and regularly updated on all servers, workstations, laptops, and other devices used in the project
Safe disposition/destruction of data or devices, as appropriate (e.g., shredding paper documents, destroying disks or thumb drives, secure erasure of electronic media)

If other please specify:

11.4* Does either statement apply to this research:
Research has NIH, CDC, or FDA funding, or other federal funding from an agency that automatically issues a Certificate of Confidentiality as part of the terms of the award:

The study will include identifiable sensitive information, identifiable biospecimens, individual human-level genomic data/biospecimens, or any information about an individual for which there is at least a very small risk, as determined by current scientific practices or statistical methods, that some combination of the information, a request for the information, and other available data sources could be used to deduce the identity of an individual.

or

Research does NOT have NIH, CDC, or FDA funding, or other federal funding from an agency that automatically issues a Certificate of Confidentiality as part of the terms of the award:

The study will include identifiable, sensitive information or identifiable biospecimens that, if revealed, might place the subjects at risk for personal safety, criminal or civil liability, or damage to their financial standing, employability, insurability, or reputation.

[Require Section 11-2]

○ Yes ● **No**

11.5* Will data be provided to a repository as part of a data sharing agreement?

○ Yes ● **No**

11.6* What will happen to the data and/or any specimens at the conclusion of this study?

Select all that apply:

Select all that apply:

Retain for future research use - requires Section 11-4

View: 11-1. Identifiable Data
Section: 11. Confidentiality, Security and Privacy

11-1. Identifiable Data

Completion of this section is required based on the response provided to question 11.1.

11-1.1* Indicate how subjects are identified in the research records.

Select all that apply:

Coded or Indirect Identifiers - data record includes a link to direct identifiers (e.g., name, initials, phone number, SSN, or medical record number linked to data record but stored separately)

No Identifiers (De-identified, Anonymous, or Anonymized) - stored data record is stripped of all identifiers

11-1.2* Explain the necessity for collecting or maintaining data linked to subjects' identities. If the information is covered in the attached protocol, please indicate section.

We are working with young children and it will be difficult to refer to them as an ID number. Therefore, their first names will be used in the data collection process along with ID number on the data recording sheet. For example, researcher will be communicating with the child and use the child's first name in the data collection process on the data record/score sheet.

In terms of the video recording, (i.e., digital file) will be coded and stored on a secured and protected server and will be deleted after the coding has been completed. The recording will capture the child's total body movement and focus on the movement that the trunk, arms, legs, and feet and for the HTKS it will have to capture the total body of the child. The researcher will then link that data for the ID number for data entry/storage. The data record/score sheet will be shredded.

We use a small 8.5 X 11.75 legal pad with one (1) ID number written on each page. As the children performs the assessments that are videotaped. We will flashed the ID number in front of the camera. This will later be used in the lab for data coding purposes.

Preschoolers will complete the motor competence assessment in small groups (i.e., 4 – 6 individuals) and tasks 20-25 minutes to complete and the HTKS will take a max of 20 minutes.
The is needed to keep the files on the UM server in case there is an error or discrepancy.

The PI will be the only individual who has assess the the participant/subject full name and their identification number. This will be stored on a hard copy file and lock in a file cabinet.

11-1.3* How long will the identifiers be retained?

The master list linking participant to the study number will be kept for 5 years after the duration of the grant and then will be destroyed through shredding of the hard copy paper file. Data kept will only be de-identified and anonymous and retained for future research purposes.

11-1.4* Will individually identifiable sensitive data be accessed, collected, used, maintained, or disclosed in the study?

○ Yes ● **No**

11-3. End of Subject Participation

11-3.1* What specific criteria will be used to prematurely end a particular subject's participation in the study (If covered in attached protocol or informed consent, indicate specific location).

When completing a test/task, a child may be having a difficult time following directions/instructions. If this occurs, we will test the child on the following day. In the past, the research team has had not had a problem with the removal of children from the study by researchers since participants are typically developing children.

Participants who refuse to participate (i.e., complete assessments after multiple attempts) will be withdrawn from the study.

11-3.2* If a participant withdraws from the research, what is the plan to use, disclose, store, or destroy the participant's data and/or specimen?

Their data will still be used for the project.

View: 11-4. Retention of Data and/or Specimens Detail
Section: 11. Confidentiality, Security and Privacy

11-4. Retention of Data and/or Specimens Detail

Retention may be for future research by the investigator and/or the creation of a bank or repository.

Completion of this section is required based on the response provided to question 11.6.

11-4.1* What is the intent or purpose of retaining the data and/or specimens?

This project investigates the immediate and long-term (3 year; grades K, 1, and 2) effects of a motor skills intervention - the Child Health and Motor Program (CHAMP) on motor competence, perceived motor competence, and physical activity in preschool-age. Specifically, the project aims to: 1) examine the immediate (post-intervention) effect of CHAMP (compared to control participants) on motor competence, perceived motor competence, and physical activity, and self-regulation in preschool-age children; 2) assess the sustainable effect of CHAMP (compared to control participants) on motor competence, perceived motor competence, and physical activity across middle childhood; and 3) examine and compare the immediate and long-term mediating effects of perceived motor competence on the relationship between motor competence and physical activity in preschool-age children. Therefore, data needs to be kept for the analyses.

11-4.2* Where will you store the data and/or specimens?

UM and Other Institutions

If Other Institutions, please specify:
Data will only be reviewed by the research team and stored on university-secured password protected computers at the University of Michigan and University of South Carolina. The research team at the University of South Carolina will review limited motor skills data for only for COHORT 2 (i.e., kicking and throwing velocity, running speed, hopping and jumping distance, and successful catching attempts) on university-secured password protected computers.

11-4.3* Describe the arrangements for the storage conditions, management, and security of the data and/or specimens. Include the following as applicable:

- ***Personnel access to data and/or specimens***
- ***Whether identifiers will be removed and the key to any code destroyed***
- ***For coded data and/or specimens, indicate who holds key to the code and where it is stored in relation to the data and/or specimens***
- ***Storage plan***
- ***Plan to protect privacy in transfer to other collaborators.***

Storage and future use of data for Cohort 1 and 2 at the University of Michigan: Data will be stored in key code access laboratory (Child Movement, Activity, and Developmental Health Lab) in CCRB 1271C and will be retained for 5 years after the conclusion of the study. Hard copy of data will be shredded (CCRB 2027). The PI will have access to the data. As previously noted, identifiable information (PI-Robinson has sole access) will be destroyed after 5 years after the conclusion of the study and ID numbers will solely be used.

Data in the laboratory will be kept in a locked filing cabinet or in a password-protected server. The identity of participants is necessary to link baseline and follow-up data. Any master sheets containing ID and identifying information will be accessible to Dr. Robinson. The master lists linking participants to study number will be destroyed after 5 years the conclusion of the study and data will only contain a numerical identification will remain on file for future research purposes.

For Cohort 2 limited motor skills data (i.e., kicking and throwing velocity, running speed, hopping and jumping distance, and successful catching attempts) that will be reviewed at the University of South Carolina, the data will be transferred to an encrypted hard drive and transported by USC investigator to USC and stored on a on university-secured password-protected computers. Data will be retained for 5 years after the conclusion of the study.

13. Subject Payments Or Other Incentives

Completion of this section is required based on the response provided to question 7-1.1 or 7-3.3.

13.1* Indicate all payments or other incentives provided to subjects for their participation in this study:

Select all that apply:

Cash

HSIP Issued Gift Card

Gift certificates (e.g. Target, Toys R Us, etc.)

Other

If other, please specify:

13.2* If the subject is a child (under the age of majority), are any of the payments or incentives intended for the parent/guardian of the child?

Yes

13.3* Estimate the maximum total payment (including cash, checks, gift cards, and other cash-equivalent incentives) that an individual subject could receive for participating in this research in a single calendar year.

$26-$100

13.3.1* Please indicate what information you will be collecting from subjects in order to distribute their incentive or compensation.

Select all that apply:

Name

Address

Email

13.4* Describe the frequency of the payments or incentives. If applicable, list any healthcare procedure(s) that will be provided to subjects at no charge.

-A total of 5 (pre, post, and 3 follow-up measures) at $10.00 each, total $50.00 to participants.
- Upon returning the IRB form and to compensate the parent/guardian for their time, parent/guardian will receive a one-time $5.00 cash incentive.
-Completed Family Questionnaires will be entered in a drawing to win one (1) of eight (8) $50.00 gift cards to Wal-Mart (i.e., 4 for each school). Two (2), $50 gift card drawings will occur each day (i.e., one (1) for each preschool) and will occur over four (4) consecutive days. ID numbers of completed surveys will enter four discrete pools on each day of the drawing (e.g. Subjects 1-37 on Day One for each site, Subjects 38 - 75 on Day Two for each site,), and Subjects 76-113 on Day Three for each site, Subjects 114-150 on Day 4 for each site). Parents will receive a yearly report regarding their child's assessment findings. Early withdrawal from this study will forfeit future compensation.

13.5* What is the justification for offering these payments or incentives?

An incentive for the children to return the physical activity monitoring device.
- To compensate parents for their time.
- To motivate parents to complete and return the questionnaire.

13.6* What is the plan to compensate subjects withdrawing from the research prior to completing the entire study.

Early withdrawal from this study will forfeit future compensation.

17. Placebo

Completion of this section is required based on the response provided to question 7-1.8 or 7-3.6.

17.1* Briefly describe the placebo (drug, device, procedure, intervention, surgery, etc.) control arm used in the study.

The control condition is the standard of care (outdoor/free play recess) for preschool programs. The control condition will be the preschools' typical activity programs (i.e., outdoor/free play recess) and will be implemented according to the existing procedures within the preschool center. The centers' outdoor program consists of outdoor free-play activity on a large playground area with a variety of play structures (swings, slides, ladders) that promote gross movement and activity in preschoolers. For the control condition, there will be no planned instruction nor activities provided by the classroom teachers.

17.2* Provide a justification for use of the placebo, including the length of subject participation in the placebo arm.

The control condition is the standard of care (outdoor/free play recess) for preschool programs. Outdoor/free play recess is the normal movement program that is provided in preschool settings where there is no planned instruction nor activities provided by the classroom teachers.

17.3* Describe any commonly used diagnostic/treatment approach(es) that will be withheld from subjects assigned to the placebo arm of this study.

N/A, no diagnostic/treatment approach(es) are being withheld from the subjects.

17.4* Is study enrollment limited to individuals for whom the commonly used diagnostic/treatment approaches are known to be ineffective or intolerable?

○ Yes ● **No**

17.5* Summarize the risk to subjects in the placebo arm who receive no active treatment for their disease or condition.

The risk associated with subjects who are not a part of the active treatment (i.e., CHAMP) group may include a decline in the motor skills, low participation in physical activity, and low perception of perceived motor skills. It is unclear as it relates to the effect on children's self-regulation, but we hypothesized that children in the active treatment will experience positive outcomes on this measure. We hope that the findings will support the need for early movement programs in preschool education programs.

17.6* How will the condition or disease of subjects in the placebo arm of this study be monitored, compared to the monitoring associated with standard care for this disease/condition?

They will be monitored the same as the treatment conditioned (assessed at all of the assessments time points). Note, this study is not examining any diseased conditions. Outcome measures are motor skills, physical activity, perceived motor skills, and self-regulation.

17.7* What criteria will be used to determine that the participation of a subject, who may be receiving a placebo treatment, should be discontinued due to his/her worsening disease or condition?

This study is not examining any diseased conditions. Outcome measures are motor skills, physical activity, perceived motor skills, and self-regulation.

29. Survey Research

Completion of this section is required based on the response provided to question 7-1.5.

29.1* **Provide a list of all surveys and interviews used in the study:**

Name	# of Questions	Duration	Sensitive?	Disturbing?
Demographics	3	5 min	no	no
Dimensional Change Card Sort Task	12	10 min	no	no
Emotional Regulation Checklist	24	20 per class	no	no
FAMILY QUESTIONNAIRE - ABOUT YOUR CHILD AND YOUR FAMILY	40	30 min	no	no
Head-Toes-Knees-Shoulder Task (HTKS Task; Behavioral Inhibition)	10	10 min	no	no
Measurement Tools for Height and Weight (Body Composition)	5	12-15 minutes	no	no
Motor Competence (product measure)	5	20-25	no	no
Movement Environment Rating Scale (MOVERS)	90	60 min per center	no	no
Observed Self-Regulation	10	10 min per child	no	no
Perceived Motor Competence	18	10 min	no	no
Play Space Assessment	11	20	no	no
POEMS Assessment Tool	56	25	no	no
Test of Gross Motor Development (Motor Competence- process measures)	12	20-25 min	no	no
Working Memory (Mr. Ant)	8	10 min	no	no

29.13* **Will the research involve the use of focus groups?**

○ Yes ● **No**

29.14* **Is any of the material disturbing?**

○ Yes ● **No**

View: VIEW000619_customAttributes._attribute234.customAttributes._attribute8_Survey Detail
Section: 29. Survey Research

Survey Detail

29.2* Survey or interview name:

Demographics

29.3* Is the design or development of this survey instrument dependent on receipt of funding or hiring of personnel?

○ Yes ● **No**

29.4* In what manner will the survey or interview be conducted (e.g., in-person, Internet, mail, telephone, etc.)? *Special Note: For electronic surveys, the eResearch ID number must be included in the informed consent document (uploaded in section 10-1) or other material that serves as the informed consent.*

Child's date of birth (i.e., month and year will be used for indentifers, but full birthdate is needed for accurate calculation of measures - body composition, process motor competence measured with the TGMD, and physical activity), sex, and race/ethnicity will be obtain (collected) from the parent/guardian through self-report on during the IRB consenting process.

29.5* What is the predicted response rate?

100 %

29.6* What is the total number of questions?

3

29.7* What is the anticipated cumulative amount of time required for each subject?

5 min

29.8* What is the total number of interviews/data collection interactions with an individual subject?

1

29.9* Does the survey or interview contain questions of a sensitive nature (e.g., mental illness, sexual abuse, illicit drug use, etc.)?

○ Yes ● **No**

29.10* Is the survey or interview likely to produce psychological discomfort or negative feelings in the subjects?

○ Yes ● **No**

29.11* Has the survey instrument been validated or used in standard practice?

● **Yes** ○ No

29.11.1* If yes, describe the origin of the instrument.

Standard practice in pediatric research. Attached are a copy of the measurement tools.

29.12* Upload the survey instrument here.

Name	Version
7.4.17 CLEAN Amendment PATH + SOBC - R01 Informed Consent.docx(0.01)	0.01

View: VIEW000619_customAttributes._attribute234.customAttributes._attribute8_Survey Detail
Section: 29. Survey Research

Survey Detail

29.2* Survey or interview name:

Dimensional Change Card Sort Task

29.3* Is the design or development of this survey instrument dependent on receipt of funding or hiring of personnel?

○ Yes ● **No**

29.4* In what manner will the survey or interview be conducted (e.g., in-person, Internet, mail, telephone, etc.)? *Special Note: For electronic surveys, the eResearch ID number must be included in the informed consent document (uploaded in section 10-1) or other material that serves as the informed consent.*

Electronic assessment administered by research staff to participant.

29.5* What is the predicted response rate?

100 %

29.6* What is the total number of questions?

12

29.7* What is the anticipated cumulative amount of time required for each subject?

10 min

29.8* What is the total number of interviews/data collection interactions with an individual subject?

2

29.9* Does the survey or interview contain questions of a sensitive nature (e.g., mental illness, sexual abuse, illicit drug use, etc.)?

○ Yes ● **No**

29.10* Is the survey or interview likely to produce psychological discomfort or negative feelings in the subjects?

○ Yes ● **No**

29.11* Has the survey instrument been validated or used in standard practice?

● **Yes** ○ No

29.11.1* If yes, describe the origin of the instrument.

The Dimensional Change Card Sort (DCCS) Task. This is an iPad based assessment and will measure of cognitive flexibility, requiring children to learn and remember a rule and apply it one way, and then apply a new rule as instructions change. Children match pictures that vary by two dimensions, shape (rabbit vs. boat) and color (brown vs. white). Children complete trials with one set of instructions, then another, and are encouraged to go as fast as they can without making mistakes. 0 = did not pass color sort (Less than 5/6 items correct on color sort), 1 = pass of color sort, fail on shape sort (At least 5/6 items correct on color sort but less than 5/6 items correct on shape sort), 2 = pass shape sort, fail advance trials (At least 5/6 items correct on both color and shape sort but less than 9/12 items correct on advance/border sort), 3 = pass advanced trails (At least 5/6 items correct on both color and shape sort and at least 9/12 items correct on advance/border sort.)
Time Frame: Measured at baseline (Month 0), post-intervention (month 9).

29.12* Upload the survey instrument here.

Name	Version
DCCS Task.pdf(0.01)	0.01

View: VIEW000619_customAttributes._attribute234.customAttributes._attribute8_Survey Detail
Section: 29. Survey Research

Survey Detail

29.2* **Survey or interview name:**

Emotional Regulation Checklist

29.3* **Is the design or development of this survey instrument dependent on receipt of funding or hiring of personnel?**

○ Yes ● **No**

29.4* **In what manner will the survey or interview be conducted (e.g., in-person, Internet, mail, telephone, etc.)?** ***Special Note: For electronic surveys, the eResearch ID number must be included in the informed consent document (uploaded in section 10-1) or other material that serves as the informed consent.***

Classroom teachers will complete the Emotion Regulation Checklist

29.5* **What is the predicted response rate?**

100 %

29.6* **What is the total number of questions?**

24

29.7* **What is the anticipated cumulative amount of time required for each subject?**

20 per class

29.8* **What is the total number of interviews/data collection interactions with an individual subject?**

2

29.9* **Does the survey or interview contain questions of a sensitive nature (e.g., mental illness, sexual abuse, illicit drug use, etc.)?**

○ Yes ● **No**

29.10* **Is the survey or interview likely to produce psychological discomfort or negative feelings in the subjects?**

○ Yes ● **No**

29.11* **Has the survey instrument been validated or used in standard practice?**

● **Yes** ○ No

29.11.1* **If yes, describe the origin of the instrument.**

Teachers will report on child emotion regulation, another key aspect of SR at this age, using the 24-item Emotion Regulation Checklist (ERC), which generates Emotion Regulation and Negative Lability subscales. Co-I Miller uses this measure frequently in her work with Head Start preschoolers and finds good reliability in similar samples (α=.76-87).
Teacher-Reported. Teachers will report on child emotion regulation, another key aspect of SR at this age, using the 24-item Emotion Regulation Checklist (ERC), which generates Emotion Regulation and Negative Lability subscales. It is a 24 item, four-point Likert scale 1 = Never to 4 = Almost Always). The mean is taken and a higher score indicates better emotional regulation (i.e., self-regulation behaviors). Lead (head) teachers from each classroom will receive $50.00 in the form of a gift card at baseline and post-intervention for completing this assessment on their children. (Previously approved in PROTOCOL from Co-I Miller).
Time Frame: Baseline (Month 0), post-intervention (month 9).

29.12* **Upload the survey instrument here.**

Name	Version
ERC_CMAH_Lab_1 inch.doc(0.01)	0.01

View: VIEW000619_customAttributes._attribute234.customAttributes._attribute8_Survey Detail
Section: 29. Survey Research

Survey Detail

29.2* Survey or interview name:

FAMILY QUESTIONNAIRE - ABOUT YOUR CHILD AND YOUR FAMILY

29.3* Is the design or development of this survey instrument dependent on receipt of funding or hiring of personnel?

○ Yes ● **No**

29.4* In what manner will the survey or interview be conducted (e.g., in-person, Internet, mail, telephone, etc.)? *Special Note: For electronic surveys, the eResearch ID number must be included in the informed consent document (uploaded in section 10-1) or other material that serves as the informed consent.*

Parents will complete the paper-based questionnaire during the consenting process. It is A descriptive questionnaire that will provide additional information on the family structure, early learning center/preschool school services, physical activity habits, sleeping habits, and screen time habits.

29.5* What is the predicted response rate?

100 %

29.6* What is the total number of questions?

40

29.7* What is the anticipated cumulative amount of time required for each subject?

30 min

29.8* What is the total number of interviews/data collection interactions with an individual subject?

1

29.9* Does the survey or interview contain questions of a sensitive nature (e.g., mental illness, sexual abuse, illicit drug use, etc.)?

○ Yes ● **No**

29.10* Is the survey or interview likely to produce psychological discomfort or negative feelings in the subjects?

○ Yes ● **No**

29.11* Has the survey instrument been validated or used in standard practice?

○ Yes ● **No**

29.12* Upload the survey instrument here.

Name	Version
Revised LER Family Survey.docx(0.01)	0.01

View: VIEW000619_customAttributes._attribute234.customAttributes._attribute8_Survey Detail
Section: 29. Survey Research

Survey Detail

29.2* Survey or interview name:

Head-Toes-Knees-Shoulder Task (HTKS Task; Behavioral Inhibition)

29.3* Is the design or development of this survey instrument dependent on receipt of funding or hiring of personnel?

○ Yes ● **No**

29.4* In what manner will the survey or interview be conducted (e.g., in-person, Internet, mail, telephone, etc.)? *Special Note: For electronic surveys, the eResearch ID number must be included in the informed consent document (uploaded in section 10-1) or other material that serves as the informed consent.*

Behavioral assessment administered by research staff

29.5* What is the predicted response rate?

100 %

29.6* What is the total number of questions?

10

29.7* What is the anticipated cumulative amount of time required for each subject?

10 min

29.8* What is the total number of interviews/data collection interactions with an individual subject?

2

29.9* Does the survey or interview contain questions of a sensitive nature (e.g., mental illness, sexual abuse, illicit drug use, etc.)?

○ Yes ● **No**

29.10* Is the survey or interview likely to produce psychological discomfort or negative feelings in the subjects?

○ Yes ● **No**

29.11* Has the survey instrument been validated or used in standard practice?

● **Yes** ○ No

29.11.1* If yes, describe the origin of the instrument.

The HTKS Task is a direct observational assessment of behavioral regulation and takes 10 minutes to complete (Ponitz et al., 200, Cameron et al., 2007). It reflects a child's ability to remember commands and to behaviorally inhibit a pre-posttest response in favor of a less-dominant response. Children are asked to play a game in which they were instructed to do the opposite of what the experimenter states. For example, the experimenter instructs them to touch their head (or their toes), and instead of following the command, children will be directed to do the opposite and touch their toes (or their head). Children are given four practice tests and the instructions are repeated up to three times during the practice tests. After the practice tests are administered, the testing portion of the task is given. There are a total of 10 items in random order, with possible item scores of 0, 1, or 2 for each item. Higher scores indicated higher levels of behavioral regulation. A 0 is incorrect, 1 is a self-correct (defined as any motion toward the incorrect response but where the child then stopped and responded correctly), and 2 points are given if a child gave the correct response without hesitation or a prior movement to the incorrect response. Scores across trials are summed to reflect self-regulation. The task has begins with 6 practice items and between the first and second set of items there are 5 more practice trials. The score range is 0-40; higher score equals better behavioral inhibition. (Procedural manual)
Time Frame: Measured at baseline (Month 0), post-intervention (month 9)

29.12* Upload the survey instrument here.

Name	Version
HTKS Guidelines_21Oct10.pdf(0.01)	0.01
HTKS_Form A Extended_12Jan11_CMAH lab_1 inch.docx(0.01)	0.01
HTKS_Form B Extended_12Jan11_CMAH lab_1 inch.docx(0.01)	0.01

View: VIEW000619_customAttributes._attribute234.customAttributes._attribute8_Survey Detail
Section: 29. Survey Research

Survey Detail

29.2* Survey or interview name:

Measurement Tools for Height and Weight (Body Composition)

29.3* Is the design or development of this survey instrument dependent on receipt of funding or hiring of personnel?

○ Yes ● **No**

29.4* In what manner will the survey or interview be conducted (e.g., in-person, Internet, mail, telephone, etc.)? *Special Note: For electronic surveys, the eResearch ID number must be included in the informed consent document (uploaded in section 10-1) or other material that serves as the informed consent.*

CMAH Lab Research Staff will complete Height and Weight measurement on the participants.

29.5* What is the predicted response rate?

100 %

29.6* What is the total number of questions?

5

29.7* What is the anticipated cumulative amount of time required for each subject?

12-15 minutes

29.8* What is the total number of interviews/data collection interactions with an individual subject?

5

29.9* Does the survey or interview contain questions of a sensitive nature (e.g., mental illness, sexual abuse, illicit drug use, etc.)?

○ Yes ● **No**

29.10* Is the survey or interview likely to produce psychological discomfort or negative feelings in the subjects?

○ Yes ● **No**

29.11* Has the survey instrument been validated or used in standard practice?

● **Yes** ○ No

29.11.1* If yes, describe the origin of the instrument.

Body Composition will be calculated using standing height will be recorded on a SECA 769 to the nearest 0.1 cm. Weight (mass) will be rounded to the nearest 0.1 kg using a digital scale. Researchers will assist preschoolers in removing their shoes and socks. Students would stand still on the appropriate scale to complete the assessment. Waist circumference will be assessed with a non-elastic plastic tape measure. The tape measure will be placed around your child's waist in a standing position. Two measures will be taken and the average of the two will be used for data analyses. Preschoolers will complete the body composition assessments in small groups of 4 – 6 and should take 12 – 15 minutes to remove/put on shoes/socks and take the assessment for each group. The assessment will be completed in a designated classroom/area at the child care center. This measure will be collected first as height and weight information along with birthdate and sex are needed for the calibration of the physical activity monitoring devices.

29.12* Upload the survey instrument here.

Name	Version
Measurement Tools for Height and Weight.docx(0.01)	0.01

View: VIEW000619_customAttributes._attribute234.customAttributes._attribute8_Survey Detail
Section: 29. Survey Research

Survey Detail

29.2* **Survey or interview name:**

Motor Competence (product measure)

29.3* **Is the design or development of this survey instrument dependent on receipt of funding or hiring of personnel?**

○ Yes ● **No**

29.4* **In what manner will the survey or interview be conducted (e.g., in-person, Internet, mail, telephone, etc.)?** ***Special Note: For electronic surveys, the eResearch ID number must be included in the informed consent document (uploaded in section 10-1) or other material that serves as the informed consent.***

Product scores will be used to assess motor skill development/competence alongside with the Test of Gross Motor Development (TGMD). Specifically, kick and throw velocity, catching percentage, hop and jump distance and running speed will be measured while the children are completing the TGMD assessments. These product scores have been previously used by the Co-I Stodden.

1. Ball velocities will be measured with a radar gun. A tennis ball 5cm and a playground ball (18cm diameter) will be thrown and kicked, respectively, with maximum effort from 20 feet for 5 trials.
2. Jump distance (cm) will be measured for the standing long jump.51 The average speed (throwing, kicking) and distance (jumping) of the best 3 out of 5 trials will be used for data collection.
3.Children will attempt to catch a tennis ball 5 times with a research staff tossing the ball with a standardized procedure according to age. The number of catches out of 5 will be used.
4. The average hop stride length (from heel to heel) for 4 hop cycles of each leg will be calculated by digitizing stride length using motion capture (Dartfish, Inc.).
4.Running speed will be calculated from four consecutive stride cycles using motion capture (Dartfish, Inc.). Hopping, running and jumping data will be normalized to a percent of standing height.
Product scores will be analyzed from videotape through video motion capture processing with Dartfish software.

29.5* **What is the predicted response rate?**

100 %

29.6* **What is the total number of questions?**

5

29.7* **What is the anticipated cumulative amount of time required for each subject?**

20-25

29.8* **What is the total number of interviews/data collection interactions with an individual subject?**

6

29.9* **Does the survey or interview contain questions of a sensitive nature (e.g., mental illness, sexual abuse, illicit drug use, etc.)?**

○ Yes ● **No**

29.10* **Is the survey or interview likely to produce psychological discomfort or negative feelings in the subjects?**

○ Yes ● **No**

29.11* **Has the survey instrument been validated or used in standard practice?**

● **Yes** ○ No

29.11.1* **If yes, describe the origin of the instrument.**

Product scores will be used to assess motor skill development/competence alongside with the Test of Gross Motor Development (TGMD). Specifically, kick and throw velocity, catching percentage, hop and jump distance and running speed will be measured while the children are completing the TGMD assessments. These product scores have been previously used by the Co-I Stodden.

1. Ball velocities will be measured with a radar gun. A tennis ball 5cm and a playground ball (18cm diameter) will be thrown and kicked, respectively, with maximum effort from 20 feet for 5 trials.
2. Jump distance (cm) will be measured for the standing long jump.51 The average speed (throwing, kicking) and distance (jumping) of the best 3 out of 5 trials will be used for data collection.
3.Children will attempt to catch a tennis ball 5 times with a research staff tossing the ball with a standardized procedure according to age. The number of catches out of 5 will be used.
4. The average hop stride length (from heel to heel) for 4 hop cycles of each leg will be calculated by digitizing stride length using motion capture (Dartfish, Inc.).
4.Running speed will be calculated from four consecutive stride cycles using motion capture (Dartfish, Inc.). Hopping, running and jumping data will be normalized to a percent of standing height.
Product scores will be analyzed from videotape through video motion capture processing with Dartfish software.

29.12* **Upload the survey instrument here.**

Name	Version

Name	Version
Motor Competence - Product Scores.docx(0.01)	0.01

View: VIEW000619_customAttributes._attribute234.customAttributes._attribute8_Survey Detail
Section: 29. Survey Research

Survey Detail

29.2* Survey or interview name:

Movement Environment Rating Scale (MOVERS)

29.3* Is the design or development of this survey instrument dependent on receipt of funding or hiring of personnel?

○ Yes ● **No**

29.4* In what manner will the survey or interview be conducted (e.g., in-person, Internet, mail, telephone, etc.)? *Special Note: For electronic surveys, the eResearch ID number must be included in the informed consent document (uploaded in section 10-1) or other material that serves as the informed consent.*

CMAH Lab Research Staff will complete this environmental assessment.

29.5* What is the predicted response rate?

100 %

29.6* What is the total number of questions?

90

29.7* What is the anticipated cumulative amount of time required for each subject?

60 min per center

29.8* What is the total number of interviews/data collection interactions with an individual subject?

2

29.9* Does the survey or interview contain questions of a sensitive nature (e.g., mental illness, sexual abuse, illicit drug use, etc.)?

○ Yes ● **No**

29.10* Is the survey or interview likely to produce psychological discomfort or negative feelings in the subjects?

○ Yes ● **No**

29.11* Has the survey instrument been validated or used in standard practice?

● **Yes** ○ No

29.11.1* If yes, describe the origin of the instrument.

Movement Environment Rating Scale (MOVERS; Archer and Siraj, 2017). MOVERS is a method of measuring the quality of movement environment where the young children are encouraged to move and be physically active. It applies the methodology used in the ECERS-E and SSTEW rating scales, making it easy for educators already familiar with these well-established scales to adopt. This descriptive scale is a series of yes/no questions and 7-point Likert question for each four subscale (curriculum environment, resources for physical development, pedagogy for physical development, support for physical activity and critical thinking. The assessment will be measured by trained, research assistants from the Child Movement, Activity, and Developmental Health Lab.

Time Frame: Baseline (Month 0) and follow-up assessment at the end of year 2.

29.12* Upload the survey instrument here.

Name	Version
MOVERS Environment Rating Scale (2017).pdf(0.01)	0.01

View: VIEW000619_customAttributes._attribute234.customAttributes._attribute8_Survey Detail
Section: 29. Survey Research

Survey Detail

29.2* **Survey or interview name:**

Observed Self-Regulation

29.3* **Is the design or development of this survey instrument dependent on receipt of funding or hiring of personnel?**

○ Yes ● **No**

29.4* **In what manner will the survey or interview be conducted (e.g., in-person, Internet, mail, telephone, etc.)?** ***Special Note: For electronic surveys, the eResearch ID number must be included in the informed consent document (uploaded in section 10-1) or other material that serves as the informed consent.***

Co-I Miller will code and analyze the HTKS and Child Assessor Report (i.e., Child Behavior Observation) data. (Previously approved in PROTOCOL from Co-I Miller).

29.5* **What is the predicted response rate?**

100 %

29.6* **What is the total number of questions?**

10

29.7* **What is the anticipated cumulative amount of time required for each subject?**

10 min per child

29.8* **What is the total number of interviews/data collection interactions with an individual subject?**

2

29.9* **Does the survey or interview contain questions of a sensitive nature (e.g., mental illness, sexual abuse, illicit drug use, etc.)?**

○ Yes ● **No**

29.10* **Is the survey or interview likely to produce psychological discomfort or negative feelings in the subjects?**

○ Yes ● **No**

29.11* **Has the survey instrument been validated or used in standard practice?**

● **Yes** ○ No

29.11.1* **If yes, describe the origin of the instrument.**

We will also evaluate child ability to stay on-task during HTKS as an aspect of SR. Incorrect responses and false starts/self-corrections are recorded in the HTKS task, with fewer such responses indicating greater SR. Child compliance and engagement will also be rated using the Child Assessor Report which has been used in prior SR trials. Co-I Miller has decades of experience coding such behaviors from video analyses and will train independent observers to code these behaviors (criterion of Cohen's Kappa>.70 or ICC>.80). Observed self-regulation will also evaluate the child ability to stay on-task during HTKS as an aspect of self-regulation. Child compliance and engagement will also be rated using the Child Assessor Report (i.e., Child Behavior Observation) which has been used in prior SR trials. This 10-item questionnaire is scored on a 0-3 scale. The mean is taken and a higher score indicates better-observed self-regulation behaviors and coded my trained research. Co-I Miller will code and analyze the HTKS and Child Assessor Report (i.e., Child Behavior Observation) data. (Previously approved in PROTOCOL from Co-I Miller).

29.12* **Upload the survey instrument here.**

Name	Version
Child Behavior Assessment_CMAH Lab_1 inch.docx(0.01)	0.01

View: VIEW000619_customAttributes._attribute234.customAttributes._attribute8_Survey Detail
Section: 29. Survey Research

Survey Detail

29.2* Survey or interview name:

Perceived Motor Competence

29.3* Is the design or development of this survey instrument dependent on receipt of funding or hiring of personnel?

○ Yes ● **No**

29.4* In what manner will the survey or interview be conducted (e.g., in-person, Internet, mail, telephone, etc.)? *Special Note: For electronic surveys, the eResearch ID number must be included in the informed consent document (uploaded in section 10-1) or other material that serves as the informed consent.*

Perceived motor competence will be assessed with the Harter and Pike Pictorial Scale of Perceived Motor Competence and Social Acceptance (PSPCSA, 1984) and the Perceived Fundamental Motor Skill Competence Scale (Robinson & Palmer 2015).
i. The PSPCSA will be used to measure the child's global perceived physical competence. The PSPCSA physical competence subscale consists of six items that are presented in pictures and each child will select a picture that is more like them. The six items included are swinging, climbing, tying shoe laces, skipping, running, and hopping. Reliability for internal consistency of the individual items ranged from .65 to .89, with a reliability of .86 for the combined subscale measure. The reliability of the total scale is .89. (Instrument is attached)
ii. The Perceived Fundamental Motor Skill Competence Scale (PFMSCS) is a video-based assessment that allows the child to see a perform executing the entire motor skill in action rather than a static picture (Robinson & Palmer, in press). The scale is the identical layout and item structure to the PSPCSA but align with the 12 fundamental motor skills of the TGMD. Reliability for internal consistency ranged from .69 to .84, with a reliability of .84 for the combined subscale measure. (Instrument is not provided as it is an video-based assessment)

For both assessments, children will (1) select the picture/video that is most like him or herself. One picture/video depicts a child who is competent/skilled and the other shows a child who is not competent/skilled; (2) focus on the designated pictures/videos and indicate whether he or she is just a "little bit" or "a lot" like that child. Separate picture/video for girls and boys will be used in accordance with the manual procedures. The range of scores for each item on the subscale is 1 (low competence) to 4 (high competence). Both assessments are established tools within the preschool population and standardized test protocols will be used. Additionally, PI-Robinson has a vast amount of experience with the implementation of the tool in preschool populations. Preschoolers will complete this assessment individually (1 on 1) with a researcher in a seated, quiet area. This assessment takes less than 10 minutes to complete.

29.5* What is the predicted response rate?

100 %

29.6* What is the total number of questions?

18

29.7* What is the anticipated cumulative amount of time required for each subject?

10 min

29.8* What is the total number of interviews/data collection interactions with an individual subject?

6

29.9* Does the survey or interview contain questions of a sensitive nature (e.g., mental illness, sexual abuse, illicit drug use, etc.)?

○ Yes ● **No**

29.10* Is the survey or interview likely to produce psychological discomfort or negative feelings in the subjects?

○ Yes ● **No**

29.11* Has the survey instrument been validated or used in standard practice?

● **Yes** ○ No

29.11.1* If yes, describe the origin of the instrument.

Assessments are established tools within the preschool population and standardized test protocols will be used. Perceived motor competence will be assessed with the Harter and Pike Pictorial Scale of Perceived Motor Competence and Social Acceptance (PSPCSA, 1984) and the Perceived Fundamental Motor Skill Competence Scale (Robinson & Palmer 2015).
i. The PSPCSA will be used to measure the child's global perceived physical competence. The PSPCSA physical competence subscale consists of six items that are presented in pictures and each child will select a picture that is more like them. The six items included are swinging, climbing, tying shoe laces, skipping, running, and hopping. Reliability for internal consistency of the individual items ranged from .65 to .89, with a reliability of .86 for the combined subscale measure. The reliability of the total scale is .89. (Instrument is attached)
ii. The Perceived Fundamental Motor Skill Competence Scale (PFMSCS) is a video-based assessment that allows the child to see a perform executing the entire motor skill in action rather than a static picture (Robinson & Palmer, in press). The scale is the identical layout and item structure to the PSPCSA but align with the 12 fundamental motor skills of the TGMD. Reliability for internal

consistency ranged from .69 to .84, with a reliability of .84 for the combined subscale measure. (Instrument is not provided as it is an video-based assessment)

For both assessments, children will (1) select the picture/video that is most like him or herself. One picture/video depicts a child who is competent/skilled and the other shows a child who is not competent/skilled; (2) focus on the designated pictures/videos and indicate whether he or she is just a “little bit” or “a lot” like that child. Separate picture/video for girls and boys will be used in accordance with the manual procedures. The range of scores for each item on the subscale is 1 (low competence) to 4 (high competence). Both assessments are established tools within the preschool population and standardized test protocols will be used. Additionally, PI-Robinson has a vast amount of experience with the implementation of the tool in preschool populations. Preschoolers will complete this assessment individually (1 on 1) with a researcher in a seated, quiet area. This assessment takes less than 10 minutes to complete.

29.12* Upload the survey instrument here.

Name	Version
Pictorial Plates Girls Preschool-Kindergarten - Physical Competence Subscale.pdf(0.01)	0.01
REVISED DSPMC Assessment.pptx(0.01)	0.01

View: VIEW000619_customAttributes._attribute234.customAttributes._attribute8_Survey Detail
Section: 29. Survey Research

Survey Detail

29.2* **Survey or interview name:**

Play Space Assessment

29.3* **Is the design or development of this survey instrument dependent on receipt of funding or hiring of personnel?**

○ Yes ● **No**

29.4* **In what manner will the survey or interview be conducted (e.g., in-person, Internet, mail, telephone, etc.)?** ***Special Note: For electronic surveys, the eResearch ID number must be included in the informed consent document (uploaded in section 10-1) or other material that serves as the informed consent.***

The research will evaluate the physical environment where the children engage in movement and physical activity.

29.5* **What is the predicted response rate?**

100 %

29.6* **What is the total number of questions?**

11

29.7* **What is the anticipated cumulative amount of time required for each subject?**

20

29.8* **What is the total number of interviews/data collection interactions with an individual subject?**

0

29.9* **Does the survey or interview contain questions of a sensitive nature (e.g., mental illness, sexual abuse, illicit drug use, etc.)?**

○ Yes ● **No**

29.10* **Is the survey or interview likely to produce psychological discomfort or negative feelings in the subjects?**

○ Yes ● **No**

29.11* **Has the survey instrument been validated or used in standard practice?**

● **Yes** ○ No

29.11.1* **If yes, describe the origin of the instrument.**

-The Head Start Play Space Assessment (Casey, 2007) is designed to assess Health Start in assessing the quality of outdoor play spaces for children ages 3 – 5. The tool help to identify the strengths and needs of an existing play space. This tool is best completed during a walk-through of a play space. For each of the eleven (11) categories in the left-hand column, consider the extent to which a play space meets the given criteria. Score the play space using the rating scale below found in the center column. Circle the number that best reflects the present state of the play space. Use the right hand column to make additional comments, such as strengths, areas for improvement, high or low priority, ideas, etc. This descriptive scale examines a series of yes/no questions and 5-point Likert question for each subscale.

29.12* **Upload the survey instrument here.**

Name	Version
Play-Space-Assessment-preschool.pdf(0.01)	0.01

View: VIEW000619_customAttributes._attribute234.customAttributes._attribute8_Survey Detail
Section: 29. Survey Research

Survey Detail

29.2* Survey or interview name:

POEMS Assessment Tool

29.3* Is the design or development of this survey instrument dependent on receipt of funding or hiring of personnel?

○ Yes ● **No**

29.4* In what manner will the survey or interview be conducted (e.g., in-person, Internet, mail, telephone, etc.)? *Special Note: For electronic surveys, the eResearch ID number must be included in the informed consent document (uploaded in section 10-1) or other material that serves as the informed consent.*

Assesses the physical environment when the children engage in movement and physical activity.

29.5* What is the predicted response rate?

100 %

29.6* What is the total number of questions?

56

29.7* What is the anticipated cumulative amount of time required for each subject?

25

29.8* What is the total number of interviews/data collection interactions with an individual subject?

2

29.9* Does the survey or interview contain questions of a sensitive nature (e.g., mental illness, sexual abuse, illicit drug use, etc.)?

○ Yes ● **No**

29.10* Is the survey or interview likely to produce psychological discomfort or negative feelings in the subjects?

○ Yes ● **No**

29.11* Has the survey instrument been validated or used in standard practice?

● **Yes** ○ No

29.11.1* If yes, describe the origin of the instrument.

-POEMS Assessment Tool (DeBord, 2007) scale that contains 56 items grouped in five domains: the "Physical Environment" takes everything from air quality to ground surface drainage into account. "Interactions" looks at how well the outdoor space facilitates a child's interactions with peers, adults, objects, and natural materials. "Play and Learning Settings" are specifically designed play settings (much like indoor centers) with storytelling circles, elevated work spaces, moveable objects, and other suggested items. The "Program" section views the outdoor space as an extension of the classroom in all curriculum areas while "Teacher/Caregiver Role" focuses on how teachers can enhance outdoor experiences for children. This descriptive scale examines a series of yes/no questions and 5-point Likert question for each subscale. The assessment will be measured by trained, research assistants from the Child Movement, Activity, and Developmental Health Lab.

29.12* Upload the survey instrument here.

Name	Version
POEMS_scanned.pdf(0.01)	0.01

View: VIEW000619_customAttributes._attribute234.customAttributes._attribute8_Survey Detail
Section: 29. Survey Research

Survey Detail

29.2* Survey or interview name:

Test of Gross Motor Development (Motor Competence- process measures)

29.3* Is the design or development of this survey instrument dependent on receipt of funding or hiring of personnel?

○ Yes ● **No**

29.4* In what manner will the survey or interview be conducted (e.g., in-person, Internet, mail, telephone, etc.)? *Special Note: For electronic surveys, the eResearch ID number must be included in the informed consent document (uploaded in section 10-1) or other material that serves as the informed consent.*

Assessment administered by research staff. Test of Gross Motor Development (Ulrich, 2000) will be used to assess motor skill development/competence. The TGMD is a measure of fundamental motor skill competence in children ages 3 to 10 years and consists of 6 locomotor (run, jump, gallop, slide, hop, and skip) and 6 object control skills (throw, catch, dribble, underhand throw, kick, one-handed forearm strike). This assessment will be completed in a large indoor space (i.e., gymnasium or designated indoor activity space) that is large enough for the children to run (i.e., ~35 feet) and project objects (i.e., balls) with their hands and feet. Children will be asked to perform each skill 3 times in accordance with the testing procedures. For this assessments, children's performance of the motor skills will be video recorded with digital video cameras by trained researchers and scored/coded in the Child Movement, Activity, and Developmental Health Lab. The video recording (i.e., digital file) will be coded and stored on a secured and protected server and will be deleted after the coding has been completed. The recording will capture the child's total body movement and focus on the movement that the trunk, arms, legs, and feet. Preschoolers will complete the motor competence assessment in small groups (i.e., 4 – 6 individuals) and tasks 20-25 minutes to complete. The TGMD is an instrument that has been developed by one of the Co-I of the grant, Dr. Dale Ulrich.

29.5* What is the predicted response rate?

100 %

29.6* What is the total number of questions?

12

29.7* What is the anticipated cumulative amount of time required for each subject?

20-25 min

29.8* What is the total number of interviews/data collection interactions with an individual subject?

6

29.9* Does the survey or interview contain questions of a sensitive nature (e.g., mental illness, sexual abuse, illicit drug use, etc.)?

○ Yes ● **No**

29.10* Is the survey or interview likely to produce psychological discomfort or negative feelings in the subjects?

○ Yes ● **No**

29.11* Has the survey instrument been validated or used in standard practice?

● **Yes** ○ No

29.11.1* If yes, describe the origin of the instrument.

The TGMD is a established tool to measure of fundamental motor skill competence in children ages 3 to 10 years. The TGMD was developed by my colleague and Co-I on the project, Dr. Dale Ulrich. Test of Gross Motor Development (Ulrich, 2000) will be used to assess motor skill development/competence. The TGMD is a measure of fundamental motor skill competence in children ages 3 to 10 years and consists of 6 locomotor (run, jump, gallop, slide, hop, and skip) and 6 object control skills (throw, catch, dribble, underhand throw, kick, one-handed forearm strike). This assessment will be completed in a large indoor space (i.e., gymnasium or designated indoor activity space) that is large enough for the children to run (i.e., ~35 feet) and project objects (i.e., balls) with their hands and feet. Children will be asked to perform each skill 3 times in accordance with the testing procedures. For this assessments, children's performance of the motor skills will be video recorded with digital video cameras by trained researchers and scored/coded in the Child Movement, Activity, and Developmental Health Lab. The video recording (i.e., digital file) will be coded and stored on a secured and protected server and will be deleted after the coding has been completed. The recording will capture the child's total body movement and focus on the movement that the trunk, arms, legs, and feet. . Preschoolers will complete the motor competence assessment in small groups (i.e., 4 – 6 individuals) and tasks 20-25 minutes to complete.

29.12* Upload the survey instrument here.

Name	Version
TGMD.docx(0.01)	0.01

View: VIEW000619_customAttributes._attribute234.customAttributes._attribute8_Survey Detail
Section: 29. Survey Research

Survey Detail

29.2* **Survey or interview name:**

Working Memory (Mr. Ant)

29.3* **Is the design or development of this survey instrument dependent on receipt of funding or hiring of personnel?**

○ Yes ● **No**

29.4* **In what manner will the survey or interview be conducted (e.g., in-person, Internet, mail, telephone, etc.)?** ***Special Note: For electronic surveys, the eResearch ID number must be included in the informed consent document (uploaded in section 10-1) or other material that serves as the informed consent.***

Electronic assessment administered by research staff to participant.

29.5* **What is the predicted response rate?**

100 %

29.6* **What is the total number of questions?**

8

29.7* **What is the anticipated cumulative amount of time required for each subject?**

10 min

29.8* **What is the total number of interviews/data collection interactions with an individual subject?**

2

29.9* **Does the survey or interview contain questions of a sensitive nature (e.g., mental illness, sexual abuse, illicit drug use, etc.)?**

○ Yes ● **No**

29.10* **Is the survey or interview likely to produce psychological discomfort or negative feelings in the subjects?**

○ Yes ● **No**

29.11* **Has the survey instrument been validated or used in standard practice?**

● **Yes** ○ No

29.11.1* **If yes, describe the origin of the instrument.**

Electronic assessment administered by research staff to participant.

29.12* **Upload the survey instrument here.**

Name	Version
Mr ANT.pptx(0.01)	0.01

View: 31. Watching/Listening to Audiovisual Materials
Section: 31. Watching/Listening to Audiovisual Materials

31. Watching/Listening to Audiovisual Materials

Completion of this section is required based on the response provided to question 7-1.6.

31.1* Please upload copies of all audio-visual materials used in the research.

Name	Version
DCCS Task.pdf(0.01)	0.01
Mr ANT.pptx(0.01)	0.01
REVISED DSPMC Assessment.pptx(0.01)	0.01

☐ **Check here to indicate that the material is not available electronically.**

31.2* Are any of the materials likely to produce psychological discomfort or negative feelings in the subjects?

○ Yes ● **No**

View: 33. Children - Interaction/Intervention Studies
Section: 33. Children

33. Children - Interaction/Intervention Studies

Completion of this section is required based on the response provided to questions in Section 6 and 9-1.1.

33.1* For each research activity conducted with children, indicate the member(s) of the study team that will conduct the activity and briefly describe their expertise with children.

The PI-Robinson will have direct access to the children during the course of the study. Leah E. Robinson, Ph.D. is an Associate Professor in the School of Kinesiology and Director of the Child Movement, Activity, and Developmental Health Lab. She has academic and research training in exercise science, human motor behavior, and child development. Her research takes a developmental approach to motor skill acquisition, physical activity, and physical health and development in pediatric populations and she has worked in school-based settings since 2004. Dr. Robinson is a Fellow in the American College of Sports Medicine and a research faculty within the University of Michigan a) Center of Human Growth and Development and b) Momentum Center.

The remaining members that will have direct and ongoing interaction with the children will be post-doctoral fellows and PhD students recruited to work specifically on this project. They will be added to the protocol once they have been selected.

The remaining members of the team (Colabianchi, Ulrich, & Wang) will have little interaction with the children and their expertise can be seen in biosketches.

AME00074157: Research Staff added:
On this amendment, PhD students, Andrews and Wengrovius, were added along with Sur, the CMAH Lab Coordinator. Sur will be assisting with the administrative aspects of the project. Four other members were added as research staff from the Miller Lab and will assist with data collection of the self-regulation measures: Leonard, Sparrentak, Bartholemew, & Chaudhry.

33.2* Describe the adequacy of the research facilities to accommodate children participating in this study.

The study will take place in children's preschool center. Facilities will be an open gymnasium space for the movement program and classroom when testing is being completed.

33.3* Permitted Categories of Research: The federal policy and regulations governing human subject protections specify that research involving children must fall into one of the following permitted categories. Check all categories of permitted research that apply to this study. The information provided here must be consistent with the information in Section 6.

Regulatory Category	Criteria
The research does not involve greater than minimal risk [45 CFR 46.404].	

33.3.1* Provide a justification for how the study complies with the selected requirement.

The study will take place in children's preschool center. Facilities will be an open gymnasium space for the movement program and classroom when testing is being completed.

33.4* Does the study require the involvement of children with any physical or mental incapacities?

○ Yes ● **No**

View: 41. Subjects Vulnerable to Coercion
Section: 41. Subjects Vulnerable to Coercion

41. Subjects Vulnerable to Coercion

Completion of this section is required based on the response provided to question 9-1.1 or 9-2.1.

The following subject populations, vulnerable to coercion or undue influence, have been identified for inclusion in the study.
Children

41.1* What is the justification for the inclusion of these subject populations?

The study focuses on the effect of a preschool motor skill intervention on motor skill development, physical activity, and perceived competence. A secondary outcome is to examine its effects on self-regulation. The target population is children.

41.2* Describe the additional safeguards that have been included in this study to protect the rights and welfare of these subjects.

We are using established and well-validated tools for the population. Additionally, the PI and research team have expertise in the area of pediatrics (i.e., pediatric exercise science, adapted physical education, child development).

View: 44. Additional Supporting Documents
Section: 44 Additional Supporting Documents

44. Additional Supporting Documents

44.1 Please upload any additional supporting documents related to your study that have not already been uploaded. Examples include, but are not limited to, data collection sheets, newsletters, subject brochures, and instructional brochures.

Name	Version
7.24.2017 Revisions to IRB.docx(0.01)	0.01
7.25.18_PATH_Child_Assent_Script_TRACKED.docx(0.01)	0.01
7.25.18_PATH_Child_RE-Assent_Script_TRACKED.docx(0.01)	0.01
7.25.18_PATH_FORD_Letter_to_Parents_TRACKED.docx(0.01)	0.01
7.25.18_PATH_PERRY_Letter_to_Parents_TRACKED.docx(0.01)	0.01
7.30.18_SCRIPT_TRACKED.docx(0.01)	0.01
IRB Reviewer Revisions HUM00133319 - 8.21.17.docx(0.01)	0.01
Pennell_certifications.pdf(0.01)	0.01
Robinson_HUM00133319_Final Report 6-20-18.pdf(0.01)	0.01
Stodden CITI Report3827164 SHARE feb 5 2017.pdf(0.01)	0.01
Stodden GCP Certificates July 2017 Stodden.pdf(0.01)	0.01
Track change IRB Research Plan PATH+SOBC 7.2017 (1).docx(0.01)	0.01
Track Changes 7.27.17 PATH + SOBC - R01 Informed Consent (1).docx(0.01)	0.01

View: 45. End Of Application
Section: 45. End of Application

45. End of Application

The form was successfully submitted. Click 'Exit' or 'Finish' to leave the form.
